# Novel, in-natural-infection subdominant HIV-1 CD8^+^ T-cell epitopes revealed in human recipients of conserved-region T-cell vaccines

**DOI:** 10.1371/journal.pone.0176418

**Published:** 2017-04-27

**Authors:** Nicola Borthwick, Zhansong Lin, Tomohiro Akahoshi, Anuska Llano, Sandra Silva-Arrieta, Tina Ahmed, Lucy Dorrell, Christian Brander, Hayato Murakoshi, Masafumi Takiguchi, Tomáš Hanke

**Affiliations:** 1 The Jenner Institute, University of Oxford, Oxford, United Kingdom; 2 Center for AIDS Research, Kumamoto University, Kumamoto, Japan; 3 HIVACAT, Irsicaixa AIDS Research Institute, Autonomous University of Barcelona, Badalona, Spain; 4 NDM Research Building, Nuffield Department of Medicine, University of Oxford, Oxford, United Kingdom; 5 Oxford NIHR Biomedical Research Centre, University of Oxford, Oxford, United Kingdom; 6 HIV Unit, Hospital de la Vall d’Hebrón, Barcelona, Spain; 7 Institució Catalana de Recerca I Estudis Avançats (ICREA), Barcelona, Spain; 8 International Research Center for Medical Sciences, Kumamoto University, Kumamoto, Japan; George Washington University, UNITED STATES

## Abstract

**Background:**

Fine definition of targeted CD8^+^ T-cell epitopes and their human leucocyte antigen (HLA) class I restriction informs iterative improvements of HIV-1 T-cell vaccine designs and may predict early vaccine success or failure. Here, lymphocytes from volunteers, who had received candidate HIVconsv vaccines expressing conserved sub-protein regions of HIV-1, were used to define the optimum-length target epitopes and their HLA restriction. In HIV-1-positive patients, CD8^+^ T-cell responses predominantly recognize immunodominant, but hypervariable and therefore less protective epitopes. The less variable, more protective epitopes in conserved regions are typically subdominant. Therefore, induction of strong responses to conserved regions by vaccination provides an opportunity to discover novel important epitopes.

**Methods:**

Cryopreserved lymphocytes from vaccine recipients were expanded by stimulation with 15-mer responder peptides for 10 days to establish short term-cell-line (STCL) effector cells. These were subjected to intracellular cytokine staining using serially truncated peptides and peptide-pulsed 721.221 cells expressing individual HLA class I alleles to define minimal epitope length and HLA restriction by stimulation of IFN-γ and TNF-α production and surface expression of CD107a.

**Results:**

Using lymphocyte samples of 12 vaccine recipients, we defined 14 previously unreported optimal CD8^+^ T-cell HIV-1 epitopes and their four-digit HLA allele restriction (6 HLA-A, 7 HLA-B and 1 HLA-C alleles). Further 13 novel targets with incomplete information were revealed.

**Conclusions:**

The high rate of discovery of novel CD8^+^ T-cell effector epitopes warrants further epitope mining in recipients of the conserved-region vaccines in other populations and informs development of HIV-1/AIDS vaccines.

**Trial registration:**

ClinicalTrials.gov NCT01151319

## Introduction

The protective role of CD8^+^ T cells against HIV-1 infection has been implicated by combined data from genome-wide association studies, viral sequence polymorphisms and replicative fitness analyses, and longitudinal maps of epitope escape [1–3]. Therefore, induction of effective CD8^+^ T cells by vaccines will likely be needed to complement induction of binding or broadly neutralizing antibodies for prevention of HIV-1 infection as well as assist HIV-1 cure.

Evidence is emerging that the CD8^+^ T-cell specificity, cognitive breadth and human lymphocyte antigen (HLA) restriction are crucial determinants of the T-cell response protective efficacy [1–3]. It follows that careful definition of optimal epitopes can critically inform T-cell vaccine design and increase the power of early prediction of vaccine success or failure. For this reason since 1998, the Los Alamos National Laboratory HIV Sequence Database (LANL-HSD; www.hiv.lanl.gov) has been collecting and posting now well over 300 of the best-defined, fine-mapped epitopes in the HIV-1 proteome restricted by over 80 HLA class I alleles as the ‘A list’ of HIV-1 CD8^+^ T-cell epitopes, while the less defined specific T-cell targets are gathered as the ‘B list’ [4].

One of the major challenges in vaccine development is the enormous capacity of HIV-1 for adaptation and diversification [5, 6]. This is because over the course of natural infection, immune responses through the actions of antibodies, CTL and perhaps innate responses drive rapid HIV-1 evolution known as immune escape [7–9]. Under this strong selective pressure, HIV-1 evolved to have immunodominant epitopes in the most variable regions of its proteins as decoys, while the more functionally/structurally conserved, harder-to-change and therefore more protective determinants remain subdominant [10, 11]. As a result, many of the potentially protective epitopes are left underutilized and/or completely ignored by the immune system. During natural infection, the initially strong CD8^+^ T-cell responses are thought to be swayed towards rapidly changing immunodominant epitopes and by the time the more protective subdominant epitopes may be targeted, damage to the immune system is already irreparable [3, 11, 12].

Harnessing the protective potential of and (re)focusing CD8^+^ T-cells on the HIV-1 conserved regions (HIVconsv) by active immunization is the central theorem of our T-cell vaccine strategy [11]. An additional advantage of this approach is that the conserved protein segments are common to most M group HIV-1 variants, and thus if successful, the vaccines could provide broad cross-clade protection. We constructed two generations of conserved-region vaccines HIVconsv and tHIVconsvX [13, 14], and showed by elution studies that the vaccine epitopes were presented by HLA class I molecules on the surface of vaccine- and HIV-1-infected cells [15, 16]. We also demonstrated in human volunteers that naturally subdominant regions when taken out of the context of the full-length viral proteins and delivered by a potent adenovirus prime-poxvirus boost regimens induced robust CD8^+^ T–cell responses capable of broad HIV-1 inhibition *in vitro* [17–19]. In the work presented here, we defined the optimal epitopes and their restricting HLA alleles induced by the first generation HIVconsv vaccines in healthy HIV-1-negative volunteers. Many of these epitopes have not been described before and a number of them are candidates for the Los Alamos HIV immunology database ‘A list’ [4].

## Materials and methods

### HIV-CORE 002 trial

The PBMC were collected in trial HIV-CORE 002 [18, 20], which was approved by the National Research Ethics Service (NRES) Committee West London (Ref: 10/H0707/52) and the UK Medicines and Healthcare products Regulatory Agency (Ref: 21584/0271/001). The study was conducted according to the principles of the Declaration of Helsinki (2008) and complied with the International Conference on Harmonization Good Clinical Practice guidelines. All volunteers gave written informed consent before participation. PBMC samples were stored in the Oxford Vaccine Centre Biobank in compliance with the UK Human Tissue Act 2004 and with NRES approval (Ref: 10/H0504/25).

### HLA typing of volunteers

Molecular typing for HLA-A, HLA-B, HLA-C & HLA-DRB was performed by The DNA Sequencing MISEQ HLA Laboratory, Weatherall Institute of Molecular Medicine, The John Radcliffe, on EDTA blood samples taken from all volunteers prior to vaccination. The DNA was purified using an ArchivePure^TM^ DNA purification kit (5 PRIME GmbH) according to the manufacturer’s instructions and typing was performed using specific oligonucleotide probes to amplify specific HLA genes by SS-PCR.

### Prediction of epitopes and HLA restriction

Candidate peptides obtained from the ICS analysis together with volunteer’s HLA data were entered into the ELF algorithm (epitope location finder; www.hiv.lanl.gov) and compared to previously reported epitopes retrieved from the CTL databases. Potential epitopes were identified based on either anchor residues that matched motifs associated with the submitted HLA’s or using the Immune Epitope Database (IEDB: www.immuneepitope.org), which predicts epitopes based on the affinity of given HLA/peptide interactions. The same data were also run on HLArestrictor with NetMHCpan version 2.4 (www.cbs.dtu.dk).

### Peptides

All peptides were at least 90% pure (GenScriptHK, Hong Kong) and were reconstituted to 10–40 mg/ml in DMSO, diluted to working stock solutions of 4 mg/ml in PBS and used in mapping studies at final concentration of 1.5 μg/ml. For titration studies, peptide concentrations ranged from 0.1 to 1000 nM.

### Generation of short-term cell lines (STCL)

Cryopreserved PBMCs were thawed and cultures set up at 1–3 x 10^6^ cells/ml in culture medium containing 1.5 μg/ml 15-mer ‘parental’ peptide and 25 ng/ml IL-7 (R & D Systems). Cultures were supplemented with 100 IU/ml IL-2 (R&D Systems) on day 3, and IL-2 plus fresh culture medium on day 7. On day 10, the cells were washed three times with culture medium, re-suspended in 1 ml of medium and placed with loose lids at 37°C, 5% CO_2_ for 48 hours to rest prior to the ICS assay.

### Intracellular cytokine staining (ICS) assay for peptide mapping

STCL cultures were placed into 96-well plates in medium containing a peptide or control, anti-CD28 and anti-CD49d at 1 μg/ml, anti-CD107a PE-Cy-7, brefeldin A at 0.8 μg/ml and Golgi Stop (BD Biosciences), cultured at 37°C, 5% CO_2_ for 6 hours, placed at +4 ^0^C overnight and stained the following morning. Prior to fixation and permeabilization, cells were stained with a viability marker (LIVE/DEAD fixable Aqua; Invitrogen) and anti-CD8 FITC- and anti-CD4 PE-conjugated mAbs (BD Biosciences). Cells were permeabilized using Fix/Perm (BD Biosciences), stained by using anti-CD3 ECD- (Beckman Coulter), anti-IFN-γ V450- (BD Biosciences) and anti-TNF-α APC- (BD Biosciences) conjugated mAbs for 30 min on ice and fixed in 1% paraformaldehyde. The data were acquired on a LSR II flow cytometer (Becton-Dickinson) and analyzed using FlowJo software (Tree Star).

The following gating strategy was employed for data analysis. A: FSC-A vs FSC-H to gate out doublets. B: FSC vs SSC wide gate to exclude cell debris. C: CD3 vs LIVE/DEAD to gate viable CD3^+^ T-cells. D: CD4 vs CD8 to gate single-positive CD4 and CD8 T cells. E: IFN-γ and TNFα CD8^+^ subsets.

### Intracellular cytokine assay (ICS) for HLA restriction

721.221 Epstein-Barr virus-transformed B-cell lines transfected with a single HLA gene were maintained in RPMI 1640 medium supplemented with 20% FC, and 40 μg/ml puromycin. For peptide pulsing, 0.6 x 10^6^ 721.221 cells were pelleted in a conical test tube, incubated in 25 μl of 200-μg/ml peptide solution for 1 hour at 37°C, washed by centrifugation three times to remove excess peptide. Effector cells were STCL generated as described above and rested for 48 hours prior to use. Targets and effectors were mixed at 1:1 ratio, minimum 2 x 10^5^ of each, in medium supplemented with anti-CD28 and anti-CD49d (Becton-Dickinson) both at 1 μg/ml. Controls were unpulsed targets plus effectors and effectors with peptide only. After incubating for 1 hour at 37°C, 10 μg/ml brefeldin A (Becton-Dickinson) was added and the cells were incubated at 37°C for a further 4–5 hours, stored overnight at +4°C, stained on the following day and analyzed as described above for the ICS assay.

## Results

### The vaccines and subjects

**Fig 1:**
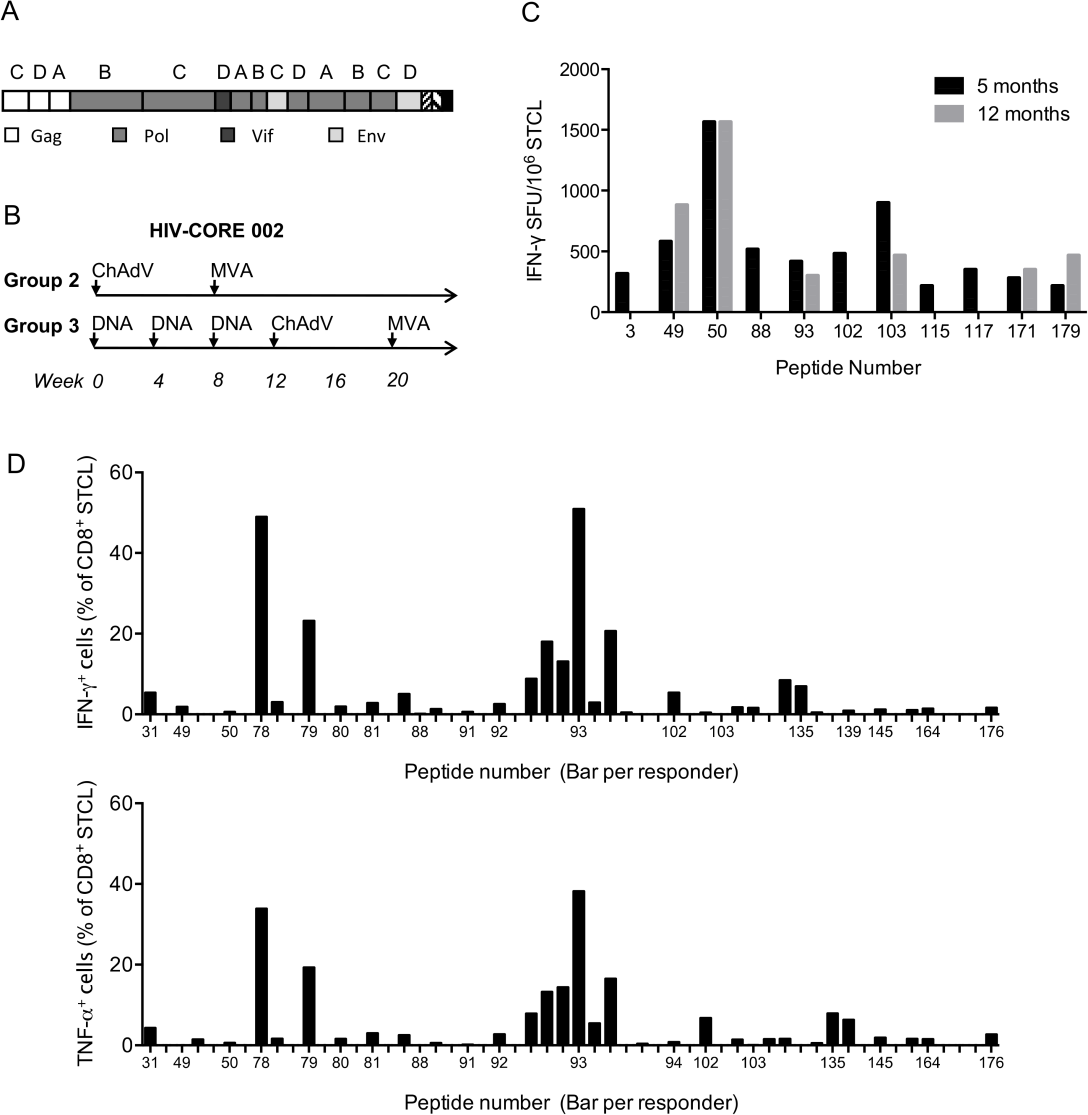
Conserved-region vaccine immunogen and the HIV-CORE 002 trial regimens. (A) The box.A schematic representation of the chimaeric design of the first generation conserved immunogen HIVconsv. Capital letters above HIVconsv regions indicate the clade of origin, from which the consensus amino sequence for that region was derived. Individual HIV-1 proteins of region origin are colour coded. (B) The two vaccine regimens of the HIV-CORE 002 trial [18] that the volunteers analyzed in this study received. ChAdV–recombinant non-replicating simian (chimpanzee) adenovirus 63 ChAdV63.HIVconsv; MVA—recombinant non-replicating poxvirus modified vaccinia virus Ankara MVA.HIVconsv; and DNA–‘naked’ plasmid pSG2.HIVconsv DNA. (C and D) Optimal epitope mapping was performed using thawed vaccine-recipients’ PBMCs, which were *in vitro* expanded with the parental 15-mer peptides for 10 days to establish STCL. (C) Frequencies of vaccine-elicited, HIV-1-specific, *in vitro* 15-mer peptide (x-axis)-expanded sequential PBMC samples of several volunteers from 5 (black) and 12 (grey) months after the last vaccine administration. Specific cells were enumerated in an IFN-γ ELISPOT assay. Panel (D) overviews the frequencies of peptide-specific CD8^+^ STCLs over the volunteers and peptides used. Frequencies of specific cells from all tested volunteers expanded by the same 15-mer peptide are shown next to each other above each peptide given on the x-axis. Top and bottom graphs show frequencies of CD8^+^ T cells producing IFN-γ and TNF-α, respectively. S2 Table for the list of peptides and responding volunteers.

The first generation HIVconsv immunogen is derived from 14 highly conserved regions of the HIV-1 proteome. It uses clade A, B, C and D consensus amino acid sequences and alternates the clade of origin for each adjacent region [13] (Fig 1A). The HIVconsv immunogen was delivered by three vaccine modalities: plasmid DNA, non-replicating simian (chimpanzee) adenovirus ChAdV-63 and non-replicating poxvirus modified vaccinia virus Ankara (MVA) in heterologous prime-boost regimens (Fig 1B). All PBMC samples used in this study were drawn from HIV-1-negative adult vaccine recipients in phase 1/2a trial HIV-CORE 002, which took place in Oxford, UK between March 2011 and April 2015 [18, 20]. All volunteers were tissue typed and each HLA-A and B allele was assigned an HLA supertype [21] (S1 Table).

### Definition of optimal CD8^+^ T-cell epitopes

A panel of 15-mer overlapping peptides (HC001-HC199) across the entire HIVconsv protein sequence was originally used to determine the breadth of HIVconsv responses in an IFN-γ ELISPOT assay [18]. Here, the PBMCs used for epitope mapping were collected between 10 weeks and 1 year after the last vaccine administration and were expanded *in vitro* using a 10-day peptide culture to establish an STCL. For selected responders and peptides, STCLs were generated from two different time points and the recognition patterns corresponded well, although individual peptide responses were with time and magnitude varied (Fig 1C and 1D). For all 12 studied individuals, optimal lengths of epitopes contained within the 15-mer peptides stimulating the up to 5 highest frequencies of HIVconsv-specific STCLs in each individual (S2 Table) were first predicted *in silico* based on the subjects’ HLAs (S3 Table). To define optimal peptides experimentally, progressively truncated peptides were used to stimulate 15-mer-expanded STCL in an ICS assay including antibodies to CD3, CD4 and CD8 markers (CD4^+^ T-cell determinants will be published separately). For most volunteers, a complete set of their HLA class I allele gene transfectants of lymphoblastoid 721.221 or C1R cells was generated to determine the restricting HLA class I allele. The results are summarized in Table 1 and their narrowing and HLA-restriction are discussed in the order of the ‘parental’ 15-mer peptide numbers and shown in S1–S19 Figs. Some parental peptides had additional lysine(s) (K or KK) added to the HIV-1-derived amino acid sequences for solubility.

**Table 1:** Mapped human CD8^+^ T-cell epitopes in the conserved regions of HIVconsv.

Expanding 15-mer peptide			Mapped epitope		Immune Epitope	Predicted HLA			Confirmed	New
Number	Sequence	VID	Name	Sequence	Database (HLA)	HLA	% rank	Aff (nM)	HLA	epitope
HC031	CTERQANFLGKIWPS	421	TL8	TERQANFL	B*40:02	B*40:02	1.5	129	ND	
			TK10	TERQANFLGK					ND	B
			CK11	CTERQANFLGK	not reported	A*11:01	0.8	55	ND	B
HC049 /HC050	**K**NFPISPIETVPVKLK /SPIETVPVKLKPGMD	406	IL8	IETVPVKL	B*40:01	B*40:01	1.0	89	ND	
						B*44:03	1.5	923	ND	
HC078	YFSVPLDEGFRKYTA	421	SK10	SVPLDEGFRK	A11	A*11:01	3.0	242	A*11:01	A
			VK9	VPLDEGFRK	not reported	NP	-	-	C*12:03	A
HC078 / HC079	YFSVPLDEGFRKYTA / PLDEGFRKYTAFTIP	417	VY10	VPLDEGFRKY	B*35:01	B*35:01	0.5	47	B*35:01	
HC080	GFRKYTAFTIPSINN	411	TI8	TAFTIPSI	A*02:01/B*51:01	B*51:01	0.8	NA	DNC	
						C*03:03	5.0	459	DNC	
			YI9	YTAFTIPSI	A*02:01	A*02:01	2.0	68	DNC	
						B*51:01	3.0	NA	DNC	
			KI10	KYTAFTIPSI	A2	C*07:01	3.0	NA	DNC	
HC081	YTAFTIPSINNETPG	415	TG11	TIPSINNETPG	not reported	NP	-	-	ND	B
			PG9	PSINNETPG	not reported	NP	-	-	DNC	B
			YI9	YTAFTIPSI	A*02:01	A*02:01	2.0	68	ND	
						C*07:02	4.0	NA	ND	
HC088	GSPAIFQSSMTKILE	409	SK11	SPAIFQSSMTK	B07	NP	-	-	B*07:02	A
			IL10	IFQSSMTKIL	Not reported	NP	-	-	ND	B
			SK11	SPAIFQSSMTK	A11	NP	-	-	A*11:01	A
		421	SM9	SPAIFQSSM	not reported	B*35:03	0.2	NA	DNC	B
			AK9	AIFQSSMTK	A*11:01	A*11:01	0.05	8.0	A*11:01	
HC091	ILEPFRAQNPEIVIY	410	FY11	FRAQNPEIVIY	not reported	NP	-	-	ND	B
			RY10	RAQNPEIVIY	not reported	A*30:02	1.5	114	ND	B
			AY9	AQNPEIVIY	A*30:02	A*30:02	1.0	77	ND	
HC092 / HC093	FRAQNPEIVIYQYMD**KK / K**NPEIVIYQYMDDLYV	410	NY9	NPEIVIYQY	B18	B*18:01	0.8	189	DNC	
			PM9	PEIVIYQYM	not reported	B*18:01	3.0	1138	B*18:01	A
			ED9	EIVIYQYMD	not reported	NP	-	-	B*18:01	A
HC093	**K**NPEIVIYQYMDDLYV	411	VV11	VIYQYMDDLYV	A*02:01	A*02:01	1.0	31	A*02:01	A
		415	IV10	IYQYMDDLYV	A*02:01	NP	-	-	A*02:01	
		416	YV9	YQYMDDLYV	A*02:01	A*02:01	0.4	10.	A*02:01	
		418	QV8	QYMDDLYV	not reported	NP	-	-	A*02:01	B
HC102	**K**QVDRMRIRTWKSLVK /	403	RK10	RIRTWKSLVK	not reported	A*30:01	0.03	3	A*30:01	A
			IK9	IRTWKSLVK	not reported	NP	-	-	A*30:01	A
HC103	MRIRTWKSLVKHHLT	418	IH10	IRTWKSLVKH	not reported	NP	-	-	B*27:05	A
HC135	KLVSQGIRKVLFLDG	416	KV10	KLVSQGIRKV	not reported	A*02:01	3.0	210	A*02:01	A
		418		KLVSQGIRKVLF	not reported	NP			ND	B
HC139	DKAQAKEIVASCDKC	404	AC11	AKEIVASCDKC	not reported	NP	-	-	ND	B
			AD9	AKEIVASCD	not reported	NP	-	-	ND	B
HC145	GQVDCSPGIWQLDCTH	404	GH9	GIWQLDCTH	not reported	NP	-	-	ND	B
HC164	VQMAVFIHNFKRKGGI	404, 410	IG9	IHNFKRKGG	not reported	NP	-	-	ND	B
HC176	VVPRRKAKIIRDYGK	413	VI10	VVPRRKAKII	not reported	NP	-	-	B*08:01	A
			RK11	RKAKIIRDYGK	not reported	NP	-	-	B*08:01	A
			KK8	KIIRDYGK	not reported	A*03:01	0.8	183	B*08:01	A

VID–volunteer identification number; **K** or **KK**–lysines added to the ‘parental’ 15-mer peptides for solubility; Immune Epitope Database (www.iedb.com)—T-cell epitope prediction for HLA binding according to HLArestrictor with NetMHCpan version 2.4: % rank–strong binding peptides <0.5, intermediate binding peptides 0.5–2.0 and >2.0 weak binding peptides (% ranks over 5.0 are not shown); Aff (nM)–affinity of peptide for HLA as approximate thermodynamic constant KD (high affinity <50 nM, intermediate affinity 50–500 nM and low affinity 5,000–500 nM); NP–not predicted; NA–no available; ND–not done; DNC–done, but no HLA-restriction confirmed. A–‘A-list’ candidate; B–‘B-list’ candidate.

### HC031 CTERQANFLGKIWPS (Gag)

Peptide HC031 was recognised by a single individual, subject with volunteer-identification (VID) number 421 (A*02:01, A*11:01, B*35:03, B*40:02, C*02:02, C*12:03), and was previously reported by us to mediate a high inhibitory activity in the *in vitro* HIV-1-inhibition assay [17]. Using truncated peptides in an ICS assay, the three equally strong peptide truncations were TERQANFL (TL8), TERQANFLGK (TK10) and CTERQANFLGK (CK11) (S1 Fig). TL8 is an ‘A-list’ epitope restricted through HLA-B*40:02, originally reported in a Hispanic HIV-1-infected woman not on combined antiretroviral treatment (cART) and also reported to elicit responses in 5 out of 20 HLA-B40-bearing, chronically infected people in China [22, 23].

### HC049 KNFPISPIETVPVKLK / HC050 SPIETVPVKLKPGMD (Pol)

Subject 406 (A*03:01, A*31:01, B*40:01, B*44:03, C*03:04, C*04:01) was reactive to these consecutive 15-mer peptides and mapping with truncated peptides identified the HLA-B*40:01-restricted, ‘A-list’ epitope IETVPVKL (IL8) [24] (S2 Fig). Five out of 20 chronically HIV-1-infected HLA-B40-positive people in China responded to IL8 as well, supporting the HLA-B*40:01 restriction in this individual [23].

### HC078 YFSVPLDEGFRKYTA (Pol)

Subject 421 (A*02:01, A*11:01, B*35:03, B*40:02, C*02:02, C*12:03), responded to two overlapping epitopes SVPLDEGFRK (SK10) and VPLDEGFRK (VK9) restricted by two respective alleles HLA-A*11:01 and HLA-C*12:03. While SK10 was previously reported to be presented by HLA-A11, VK9 was not predicted nor reported and could be weakly presented by HLA-A*11:01, too. Thus, both SK10 and VK9 epitopes are new ‘A-list’ candidates (S3 Fig). An HLA-A*11:01-restricted epitope SVPLDE**S**FRK was reported in HIV-1-exposed uninfected sex workers from Thailand [25]. The 10-mer sequence is also supported by data on length variants of eluted HLA-A*11:01-restricted epitopes [26].

### HC078 YFSVPLDEGFRKYTA / HC079 PLDEGFRKYTAFTIP (Pol)

Subject 417 (A*03:01, A*30:04, B*35:01, B*50:01, C*04:01, C*06:02) was reactive to these consecutive 15-mer peptides. Using truncated peptides initially suggested a strong response to PLDEGFRKY (PY9), while 8-mers LY8 and PK8 did not elicit any response. Subsequent studies using single HLA-transfected 721.221 cells showed that PY9 is restricted through HLA-B*35:01, however, the longer HLA-B*35:01-restricted VPLDEGKFRKY (VY10), which is already an ‘A-list’ peptide and is present in HC078, but not HC079, was the optimal epitope [27] (S4 Fig).

### HC080 GFRKYTAFTIPSINN (Pol)

The 8-mer TAFTIPSI (TI8) [28] was recognised by volunteer 411 (A*02:01, A*02:01, B*08:01, B*51:01, C*03:03, C*07:01) probably through HLA-B*51:01, although it is also a published HLA-A*02:01 epitope [29], which 411 also expresses. In titration experiments, volunteer 411 recognised equally well YTAFTIPSI (YI9) reported as restricted through A2 [30] and KYTAFTIPSI (KI10) predicted as restricted by HLA-C*07:01. It was not possible to confirm the HLA restriction in 411 due to high background responses to the unpulsed transfected antigen-presenting cell lines (S5 Fig). The epitope YTAFTIPSI was reported in vaccine recipients of the STEP study [30].

### HC081 YTAFTIPSINNETPG (Pol)

This peptide was recognised by volunteer 415 (A*02:01, A*03:01, B*07:02, B*44:02, C*07:02, C*07:02) through HLA-A*02:01. Mapping with truncated peptides and overlapping 9-mers indicated a weak YTAFTIPSI response; a C-terminal variant (YTAFTIPS**V**) was described previously. The strongest peptide TIPSINNETPG (TG11) is an unlikely epitope due to many undesired amino acids at the C-terminus [31]. In addition, the 9-mer peptide PSINNETPG (PG9) was recognized as well, but its restriction could not be confirmed due to lack of PBMCs (S6 Fig). While TG11 is not a published epitope, IPS**T**NNEPG was reported as a conserved HLA-B7-restricted Pol epitope [32].

### HC088 GSPAIFQSSMTKILE (Pol)

This peptide was recognised by volunteers 409 and 421. Initial epitope mapping in volunteer 409 (A*01:01, A*03:01, B*07:02, B*08:01, C*07:01, C*07:02) indicated, that SPAIFQSSMTK (SK11) was the reactive sequence. This longer peptide contains two overlapping ‘A-list’ epitopes SPAIFQSSM (SM9) restricted through HLA-B*07:02 [33] and AIFQSSMTK (AK9) restricted through HLA-A*03:01 [34]. 721.221 cells transfected with HLA-B*07:02 showed that the longer peptide SK11 was the only one recognised and confirmed its HLA-B*07:02 restriction. Additional epitope IFQSSMTKIL (IL10) was also indicated in 409, but its restriction was not determined (S7 Fig). Mapping studies using STCL from volunteer 421 (A*02:01, A*11:01, B*35:03, B*40:02, C*02:02, C*12:03) again indicated SK11 as the recognized epitope, which has been reported several times in association with a range of HLAs including HLA-B07 and HLA-A11 [35]. Here, we confirmed SK11 to be restricted by HLA-A*11:01. SK11 contains two 9-mer epitopes SM9 and AK9 with predicted presentation by HLA-B*35:03 and HLA-A*11:01, respectively, but restriction studies confirmed only AK9 to be restricted through HLA-A*11:01 as there was no response through HLA-B*35:03 with the predicted epitope SM9 (S8 Fig). AK9 is an ‘A-list’ epitope described to be presented by HLA-A*11:01 and was eluted from HLA class I of HIVconsv vaccines-infected Jurkat cells (A*03:01, A*03:01, B*07:02, B*35:03, C*04:01, and C*07:02) [36], suggesting it can be presented by HLA-A*03 as well [33, 37].

### HC091 ILEPFRAQNPEIVIY (Pol)

Volunteer 410 (A*30:02, A*30:02, B*18:01, B*57:03, C*07:01, C*18:01) recognised 15-mer peptide HC091 and initial mapping suggested FRAQNPEIVIY (FY11). This 11-mer contains three predicted overlapping epitopes AQNPEIVIY (AY9) and RAQNPEIVIY (RY10) both restricted through HLA-A*30:02, and FRAQNPEIVI (FI10) restricted through HLA-C*18:01. Titration between 0.1 nM and 1000 nM peptides on STCL showed similar affinities for FY11, RY10 and AY9, all stronger than HC091, while FI10 showed no stimulation (S9 Fig). We were unable to confirm the restriction of these three stimulatory peptides, but **K**QNPEIVIY and AY9 are reported to be HLA-A*30:02 restricted and the former is on the ‘A list’ [38].

### HC092 FRAQNPEIVIYQYMD(KK) /HC093 (K)NPEIVIYQYMDDLYV (Pol)

Volunteer 410 (A*30:02, A*30:02, B*18:01, B*57:03, C*07:01, C*18:01) recognised both of these overlapping peptides. Mapping with overlapping 9-mers suggested three epitopes NPEIVIYQY (NY9), PEIVIYQYM (PM9) and EIVIYQYMD (ED9). Restriction studies confirmed ED9 and PM9 both to be restricted through HLA-B*18:01 (S10 Fig) and are both ‘A-list’ candidates. Although we were unable to confirm NY9, it is an ‘A-list’ epitope reported as restricted through HLA-B18 [39].

### HC093 (K)NPEIVIYQYMDDLYV (Pol)

Volunteers 411, 415, 416 and 418 shared HLA-HLA-A*02:01 and their HC093 STCL responded to the VIYQYMDDLYV (VV11), IYQYMDDLYV (IV10), YQYMDDLYV (YV9) and QYMDDLYV (QV8) peptides. The HLA-A*02:01 restriction was confirmed by single allele expressing 721.221 cells. Using PBMCs of volunteer 416, STCL were expanded with all 4 peptides and cross-tested against all 4 peptides. This experiment indicated that in this volunteer, predominantly VV11- and YV9-specific CD8^+^ T-cell responses were elicited (S11 Fig). VV11 is a novel ‘A-list’ candidate. Volunteer 417 (A*03:01, A*30:04, B*35:01, B*50:01, C*04:01, C*06:02) recognized HC093, had no HLA-A*02:01 and the patterns suggested several overlapping epitopes (S12 Fig). These weren’t further investigated.

### HC102 (K)QVDRMRIRTWKSLVK (Vif)

Using truncated peptides of HC102, peptide MRIRTWKSLVK (MK11) was identified in volunteer 403 (A*01:01, A*30:01, B*13:02, B*39:01, C*06:02, C*07:01). This longer sequence contains two predicted CD8^+^ T-cell epitopes RIRTWKSLVK (RK10) restricted by HLA-A*30:01 and MRIRTWKSL (ML9) predicted to be restricted by HLA-B*39:01. Using HLA-A*30:01-transfected 721.221 cell line, RK10 reactivity was confirmed. This peptide is an ‘A-list’ epitope for HLA-A*03:01 [40, 41], but was not previously shown as restricted through HLA-A*30:01. There was also some activity with the peptide IRTWKSLVK (IK9) again through HLA-A*30:01 (S13 Fig); this epitope has not been previously reported. In addition, epitope RTWKSLVK (RK8) was not tested here, but has been eluted from HLA class I of HIVconsv vaccine-infected Jurkat cells and predicted to be HLA-A*03:01 restricted [36].

### HC103 MRIRTWKSLVKHHLT (Vif)

HC103 was recognised by volunteer 418 (A*02:01, A*24:02, B*07:02, B*27:05, C*01:02, C*07:02). Using truncated peptides, the epitope IRTWKSLVKH (IH10) was indicated as the optimal epitope. This was confirmed and its restriction determined as HLA-B*27:05 (S14 Fig). IH10 is a novel ‘A-list candidate.

### HC135 KLVSQGIRKVLFLDG (Pol)

Volunteers 416 (A*02:01, A*02:01, B*08:01, B*44:02, C*05:01, C*07:01) and 418 (A*02:01, A*24:02, B*07:02, B*27:05, C*01:02, C*07:02) initially responded to peptide HC135 and, using truncated peptides, KLVSQGIRKV (KV10) was suggested as the optimal epitope. This was confirmed in both volunteers and the restriction shown to be HLA-A*02:01. This is a novel ‘A-list” epitope. Also KLVSQGIRKVLF (KF12) was recognized strongly by HC135 STCL, but its restriction was not confirmed (S15 Fig).

### HC139 DKAQ-AKEIVASCDKC (Pol)

Volunteer 404 (A*68:01, A*68:01, B*44:02, B*51:01, C*07:04, C*14:02) responded to 15-mer HC139 with a region junction between amino acids 4/5 indicated by hyphen. Mapping with truncated peptides suggested peptides AKEIVASCDKC (AC11) and AKEIVASCD (AD9). The longer peptide contains two predicted epitopes EIVASCDK (EK8) and KEIVASCDKC (KC10) restricted by HLA-A*68:01 and HLA-B*44:02, respectively, but we were unable to confirm any HLA restriction in this individual possibly due to low frequencies of responding T cells (S16 Fig).

### HC145 GQVDCSPGIWQLDCTH (Pol)

Volunteer 404 (A*68:01, A*68:01, B*44:02, B*51:01, C*07:04, C*14:02) showed reactivity to peptide HC145. Overlapping 9-mers indicated GIWQLDCTH (GH9) as the optimal epitope, however, there are no predicted or reported epitopes in the sequence. The response to the 9-mer was confirmed, but not its HLA restriction (S17 Fig).

### HC164 VQMAVFIHNFKRKGGI (Pol)

Two volunteers 404 (A*68:01, A*68:01, B*44:02, B*51:01, C*07:04, C*14:02) and 410 (A*30:02, A*30:02, B*18:01, B*57:03, C*07:01, C*18:01) responded to 15-mer peptide HC164 and mapping with overlapping 9-mers showed both shared the epitope IHNFKRKGG (IG9). This is not a reported or predicted epitope. No HLA restrictions analyses were attempted (S18 Fig).

### HC176 VVPRRKAKIIRDYGK (Pol)

Volunteer 413 (A*01:01, A*03:01, B*08:01, B*44:02, C*05:01, C*07:01) responded to 15-mer HC176 and studies with truncated and overlapping 9-mers suggested epitopes VVPRRKAKII (VI10), RKAKIIRDDYGK (RK11), and KIIRDYGK (KK8). VI9 is a reported epitope in rhesus macaques [42], but not in humans. Also 8-mers VPRRKAKI (VI8) and VVPRRKAK (VK8) induced responses in HC176 STCL and these were restricted through HLA-B*08:01 (S19 Fig). KK8 was reported in HIV-1-positive Han Chinese, although the restriction was not determined [43].

## Discussion

Our T-cell vaccines against HIV-1 express immunogen HIVconsv and aim to focus CD8^+^ effector T cells on the functionally conserved, typically subdominant epitopes [44]. This approach has now been tested in 8 clinical trials in Europe and Africa (refs. [18, 19, 45] and unpublished) generating a wealth of cryopreserved PBMC samples with vaccine-elicited CD8^+^ T-cell responses. In the work presented here, these samples were utilized for definition of novel T-cell epitopes that have the potential to serve as important targets of an effective HIV-1-specific T-cell response. Overall, we identified in the HIV-1 proteome 14 previously unreported, and therefore considered novel, ‘A-list’ epitope candidates (6 HLA-A, 7 HLA-B and 1 HLA-C) and further 13 novel CD8^+^ T-cell targets that add to the epitope ‘B list’ (Table 1). These epitopes were derived mostly from HIV-1 Pol, but also Gag and Vif proteins. While discovery of novel epitopes is not unexpected given the subdominance of these epitopes in naturally-infected HIV-1 patients, the novelty of a small proportion of these could also come from the fact that our vaccines employ ‘artificial’ clade consensus amino acid sequences, which are processed into peptides that may not all be present in the natural HIV-1 isolates. Nevertheless, the vaccine-elicited CD8^+^ T cells *in vitro* proliferated and were plurifunctional producing IFN-γ and TNF-α and expressing cell surface CD107a, a degranulation marker, upon a specific peptide stimulus. The IFN-γ, TNF-α and CD107a responses concurred well. While it is reassuring that epitopes AIFQSSMTK (Pol) and RTWKSLVK (Vif) were eluted from HLA class I molecules on HIVconsv vaccine-infected human cells [36], the relevance of many of the other subdominant epitopes in terms of their presentation on HIV-1-infected cells is being currently studied in *in vitro* HIV-1 inhibition assays [17–19, 46] and in HIV-1-potisitive patients [47]. However, the latter will be likely less useful given the epitope subdominance in natural infection.

In conclusion, *in vivo* induction of CD8^+^ T-cell effectors, their antiviral activity and assessment of their role in HIV-1 control rely strongly on unequivocal definition of the targeted epitopes and their HLA restriction collected as the ‘A-list’ epitopes [4]. As more vaccine candidates enter clinical trials, immunogen selection and *in vitro* monitoring of vaccine success become critical for go/no go developmental decisions. Through identification of new conserved targets on HIV-1 proteins, the information gathered in the present study contributes to the carefully collected list of regions of HIV-1 vulnerability and will help inform iterative improvements towards truly effective T-cell vaccines and their target product profile definition.

## Supporting information

S1 Fig HC031 CTERQANFLGKIWPS (Gag)—Definition of CD8^+^ T-cell determinants. (A) The box. 15-mer peptide HC031 was recognized by volunteer 421 of the indicated HLA type, and the optimal peptides and determined HLA restriction are summarized. Cryopreserved lymphocytes from the vaccine recipient were expanded by stimulation with 'parental' 15-mer responder peptide for 10 days to establish STCL effector cells. These were subjected to ICS using serially truncated (B), and overlapping 9-mer (C) peptides. In (B), IFN-γ (green) and TNF-α (orange) production and surface expression of CD107a (pink) served as the read-out. Arrows next to an amino acid indicate the peptide-terminal amino acid residue required for efficient peptide recognition.
(PDF)

S2 Fig HC049 KNFPISPIETVPVKLK / HC050 SPIETVPVKLKPGMD (Pol)—Definition of CD8^+^ T-cell determinants. (A) The box. Overlapping 'parental' peptides HC049 and HC050 were recognized by volunteer 406 of the indicated HLA type. Optimal peptide and its HLA restriction are shown. (B) The subject’s cryopreserved lymphocytes were expanded by stimulation with the 'parental' responder peptide for 10 days to establish STCL effector cells, which were tested in ICS using serially truncated peptides, whereby IFN-γ (green) and TNF-α (orange) production and surface expression of CD107a (pink) were detected. Arrows with an amino acid on the horizontal-bar graphs indicate the peptide-terminal amino acid residue required for efficient peptide recognition.
(PDF)

S3 Fig HC0078 YFSVPLDEGFRKYTA (Pol)—Definition of CD8^+^ T-cell determinants. (A) The box. 15-mer peptide HC078 was recognized by volunteer 421 of the indicated HLA type. Optimal peptides and their HLA restriction are summarized. (B) Cryopreserved lymphocytes were expanded by stimulation with 'parental' 15-mer responder peptide for 10 days to establish STCL effector cells. These were subjected to ICS using serially truncated peptides monitoring IFN-γ (green) and TNF-α (orange) production and surface expression of CD107a (pink). Arrows next to an amino acid indicate the peptide-terminal amino acid residue required for efficient peptide recognition. (C) The same SCTLs were stimulated using overlapping 9-mer peptide-pulsed 721.221 cells transfected with either HLA-A*11:01 (left) or HLA-C*12:03 (right).
(PDF)

S4 Fig HC078 YFSVPLDEGFRKYTA / HC079 PLDEGFRKYTAFTIP (Pol)—Definition of CD8^+^ T-cell determinants. (A) The box. 15-mer peptides HC078 and HC079 were recognized by volunteer 417 of the indicated HLA type. Optimal peptide and its HLA restriction are shown. Cryopreserved lymphocytes were expanded by stimulation with 'parental' 15-mer responder peptide for 10 days to establish an STCL, which was subjected to ICS using serially truncated (B), and overlapping 9-mer or 8-mer (C) peptides. IFN-γ (green) and TNF-α (orange) production and surface expression of CD107a (pink) served as the read-out. Arrows next to an amino acid indicate the peptide-terminal amino acid residue required for efficient peptide recognition. (D) 721.221 cells expressing individual HLA class I alleles of VID 417 were utilized for determination of HLA restriction for peptides PLDEGFRKY. (E) Peptides PLDEGFRKY and VPLDEGFRKY were tested side-by-side on HLA-B*35:01-transfected 721.221 cells for optimal stimulation of HC078-expanded SCTL.
(PDF)

S5 Fig HC080 GFRKYTAFTIPSINN (Pol)—Definition of CD8^+^ T-cell determinants. (A) The box. 15-mer peptide HC080 was recognized by volunteer 411 of the indicated HLA type. Optimal peptides are listed below. Cryopreserved 411 lymphocytes were expanded by stimulation with 'parental' 15-mer peptide for 10 days to establish STCL effector cells. These were subjected to ICS using serially truncated (B), and overlapping 9-mer or 8-mer (C) peptides monitoring IFN-γ (green) and TNF-α (orange) production and surface expression of CD107a (pink). In (B), arrows next to an amino acid indicate the peptide-terminal amino acid residue required for efficient peptide recognition. (D) Three strong stimulatory peptides were subjected to titration against the HC080-expanded SCTL.
(PDF)

S6 Fig HC081 YTAFTIPSINNETPG (Pol)—Definition of CD8^+^ T-cell determinants. (A) The box. 15-mer peptide HC081 was recognized by volunteer 415 of the indicated HLA type. While restricting HLAs were not determined for the three stimulatory peptides, the HLA-A*02:01 restriction for the ‘parental’-derived peptide was confirmed. Volunteer’s lymphocytes were expanded by stimulation with peptide HC081 for 10 days to establish an STCL, which was subjected to ICS using serially truncated (B), and overlapping 9-mer (C) peptides. IFN-γ (green) and TNF-α (orange) production and surface expression of CD107a (pink) served as the read-out. Arrows next to an amino acid indicate the peptide-terminal amino acid residue required for efficient peptide recognition. (D) 721.221 and C1R cells expressing individual HLA class I alleles of volunteer 415 were utilized to determine the HLA restriction of ‘parental’ peptide HC081.
(PDF)

S7 Fig HC088 GSPAIFQSSMTKILE (Pol)—Definition of CD8^+^ T-cell determinants. (A) The box. 15-mer peptide HC088 was recognized by volunteer 409 of the shown HLA type. Optimal peptides and determined HLA restriction are summarized below. Volunteer’s lymphocytes were expanded by stimulation with peptide HC088 for 10 days to establish an STCL, which was subjected to ICS using serially truncated (B), and overlapping 9-mer (C) peptides monitoring IFN-γ (green) and TNF-α (orange) production and surface expression of CD107a (pink). In (B), arrows next to an amino acid indicate the peptide-terminal amino acid residue required for efficient peptide recognition. (D) 721.221 cells expressing the HLA-B*07:02 and HLA-B*08:01 alleles were used to determine the HLA restriction of peptide SPAIFQSSMTK.
(PDF)

S8 Fig HC088 GSPAIFQSSMTKILE (Pol)—Definition of CD8^+^ T-cell determinants. (A) The box. 15-mer peptide HC088 was recognized by volunteer 421 of the indicated HLA type. Optimal peptides and determined HLA restriction are shown below. Volunteer’s lymphocytes were expanded by stimulation with peptide HC088 for 10 days to establish an STCL, which was subjected to ICS using serially truncated (B), and overlapping 9-mer (C) peptides monitoring IFN-γ (green) and TNF-α (orange) production and surface expression of CD107a (pink). In (B), arrows next to an amino acid indicate the peptide-terminal amino acid residue required for efficient peptide recognition. (D) 721.221 cells expressing the HLA-A*11:01 (top) and HLA-B*35:03 (bottom) alleles were used to determine the HLA restriction of overlapping 9-mer peptides.
(PDF)

S9 Fig HC091 ILEPFRAQNPEIVIY (Pol)—Definition of CD8^+^ T-cell determinants. (A) The box. 15-mer peptide HC091 was recognized by volunteer 410 of the shown HLA type. Optimal peptides and known restricting HLA allele are listed below. Cryopreserved 410 lymphocytes were expanded by stimulation with the 'parental' 15-mer peptide for 10 days to establish STCL effector cells. These were subjected to ICS using serially truncated (B), and overlapping 9-mer or 8-mer (C) peptides monitoring IFN-γ (green) and TNF-α (orange) production and surface expression of CD107a (pink). (D) Peptides were also subjected to titration against the HC091-expanded SCTL.
(PDF)

S10 Fig HC092 FRAQNPEIVIYQYMDKK / HC093 KNPEIVIYQYMDDLYV (Pol)—Definition of CD8^+^ T-cell determinants. (A) The box. Overlapping peptides HC092 and HC093 were recognized by volunteer 410 of the indicated HLA type. Optimal peptides and determined HLA restriction are shown. Volunteer’s lymphocytes were expanded by stimulation with peptide ‘parental peptides for 10 days to establish STCLs, which were subjected to ICS using serially truncated (B), and overlapping 9-mer (C) peptides monitoring IFN-γ (green) and TNF-α (orange) production and surface expression of CD107a (pink). In (B), arrows next to an amino acid indicate the peptide-terminal amino acid residue required for efficient peptide recognition. (D) 721.221 cells expressing the volunteer’s HLA alleles were used to determine the HLA restriction of peptide EIVIYQYMD.
(PDF)

S11 Fig HC093 KNPEIVIYQYMDDLYV (Pol)—Definition of CD8^+^ T-cell determinants. (A) The box. Peptide HC093 was recognized by volunteers 411, 415, 416 and 418 of the indicated HLA types. Stimulatory HLA-A*02:01 peptides are listed below. (B) Volunteers’ lymphocytes were expanded using the HC093 peptide for 10 days to establish STCLs, which were subjected to ICS using serially truncated and overlapping 9-mer or 8-mer peptides monitoring IFN-γ (green) and TNF-α (orange) production and surface expression of CD107a (pink). Arrows next to an amino acid indicate the peptide-terminal amino acid residue required for efficient peptide recognition. (C) 418’s STCL were tested for recognition of epitope variants. (D) 721.221 cells expressing HLA alleles of volunteer 416 were used to determine the HLA restriction of peptide QYMDDLYV. (E) STCLs of volunteer 416 were expanded using one of the four PV11, IV10, YV9 or QV8 peptides and tested against decreasing concentrations of all four peptides.
(PDF)

S12 Fig HC093 KNPEIVIYQYMDDLYV (Pol)—Definition of CD8^+^ T-cell determinants. (A) The box. Peptide HC093 was recognized by volunteer 417 of the shown HLA type (HLA-A*02:01-negative). (B) Cryopreserved lymphocytes were expanded with peptide HC093 for 10 days to establish STCL effector cells, which were subjected to ICS using serially truncated peptides monitoring IFN-γ (green) and TNF-α (orange) production and surface expression of CD107a (pink).
(PDF)

S13 Fig HC102 KQVDRMRIRTWKSLVK (Vif)—Definition of CD8^+^ T-cell determinants. (A) The box. Peptide HC102 was recognized by volunteer 403 of the indicated HLA type and the two optimal peptides and HLA restriction are shown. Cryopreserved lymphocytes from the vaccine recipient were expanded by stimulation with 'parental' responder peptide for 10 days to establish STCL, which was subjected to ICS using serially truncated (B), and overlapping 9-mer (C) peptides. In (B), IFN-γ (green) and TNF-α (orange) production and surface expression of CD107a (pink) served as the read-out. (D) 721.221 and C1R cells expressing all HLA alleles of volunteer 416 were used to determine the HLA restriction of the HC102-derived peptide. (E) HC102-expanded SCTL stimulated with either 721.221 cells transfected with HLA-A*30:01 (top) or just the peptide (bottom) and analysed using flow cytometry.
(PDF)

S14 Fig HC103 MRIRTWKSLVKHH-LT (Vif)—Definition of CD8^+^ T-cell determinants. (A) The box. Peptide HC103 was recognized by volunteer 418 of the indicated HLA type and the optimal peptide and its HLA restriction are shown. '-' indicates junction between two adjacent HIVconsv regions. (B) Cryopreserved lymphocytes from the vaccine recipient were expanded by stimulation with 'parental' peptide for 10 days to establish STCL, which was subjected to ICS using serially truncated peptides. IFN-γ (green) and TNF-α (orange) production and surface expression of CD107a (pink) served as the read-out. Arrows next to an amino acid indicate the peptide-terminal amino acid residue required for efficient peptide recognition. (C) 721.221 and C1R cells expressing all HLA alleles of volunteer 418 were used to determine the HLA restriction of the HC103-derived peptide by restimulation followed by flow cytometry.
(PDF)

S15 Fig HC135 KLVSQGIRKVLFLDG (Pol)—Definition of CD8^+^ T-cell determinants. (A) The box. 15-mer peptide HC135 was recognized by volunteers 416 and 418 of the indicated HLA types. Optimal peptides and determined HLA restriction are shown. (B) Volunteers’ lymphocytes were expanded by stimulation with ‘parental’ peptide for 10 days to establish STCLs, which were subjected to ICS using serially truncated peptides monitoring IFN-γ (green) and TNF-α (orange) production and surface expression of CD107a (pink). Arrows next to an amino acid indicate the peptide-terminal amino acid residue required for efficient peptide recognition. (C) The same SCTLs from volunteers 416 (left) and 418 (right) were tested for recognition of overlapping 9-mer peptides. 721.221 and C1R cells expressing HLA alleles of volunteers 416 (D) and 417 (E) were used to determine the HLA restriction of peptide KLVSQGIRKV.
(PDF)

S16 Fig HC139 DKAQ-AKEIVASCDKC (Pol)- definition of CD8^+^ T-cell determinants. (A) The box. Peptide HC139 was recognized by volunteer 404 of the indicated HLA type and the optimal peptides are shown. '-' indicates junction between two adjacent HIVconsv regions. (B) Cryopreserved lymphocytes from the vaccine recipient were expanded by stimulation with 'parental' peptide for 10 days to establish STCL, which was subjected to ICS using serially truncated peptides. IFN-γ (green) and TNF-α (orange) production and surface expression of CD107a (pink) served as the read-out. Arrows next to an amino acid indicate the peptide-terminal amino acid residue required for efficient peptide recognition. (C) The same SCTLs as in (B) were tested for recognition of overlapping 9-mer peptides.
(PDF)

S17 Fig HC145 GQVDCSPGIWQLDCTH (Pol)—Definition of CD8^+^ T-cell determinants. (A) The box. Peptide HC145 was recognized by volunteer 404 of the indicated HLA type, and the optimal peptide is shown. (B) Cryopreserved lymphocytes from the vaccine recipient were expanded by stimulation with the 'parental' peptide for 10 days to establish STCL, which was subjected to ICS using serially truncated peptides. IFN-γ (green) and TNF-α (orange) production and surface expression of CD107a (pink) served as the read-out. Arrows next to an amino acid indicate the peptide-terminal amino acid residue required for efficient peptide recognition. (C) The same SCTLs were tested for recognition of overlapping 9-mer peptides.
(PDF)

S18 Fig HC164 VQMAVFIHNFKRKGGI (Pol)—Definition of CD8^+^ T-cell determinants. (A) The box. Peptide HC164 was recognized by volunteers 404 and 410 of the indicated HLA types, and the optimal peptide is shown. (B) Cryopreserved lymphocytes from vaccine recipients 404 (left) and 410 (right) were expanded by stimulation with the 'parental' peptide for 10 days to establish STCLs, which were tested for recognition of overlapping 9-mer peptides.
(PDF)

S19 Fig HC176 VVPRRKAKIIRDYGK (Pol)—Definition of CD8^+^ T-cell determinants. (A) The box. Peptide HC176 was recognized by volunteer 413 of the indicated HLA type. Optimal peptides and their HLA restriction are shown. (B) Cryopreserved lymphocytes from the vaccine recipient were expanded by stimulation with 'parental' peptide for 10 days to establish STCL, which was subjected to ICS using serially truncated peptides. IFN-γ (green) and TNF-α (orange) production and surface expression of CD107a (pink) served as the read-out. Arrows next to an amino acid indicate the peptide-terminal amino acid residue required for efficient peptide recognition. (C) The same SCTLs as in (B) were tested for recognition of overlapping 9-mer peptides. (D) 721.221 cells expressing HLA-B*08:01 were used to confirm binding peptides.
(PDF)

S1 Table List of studied vaccine recipients and their HLA class I alleles and supertypes. (PDF)

S2 Table Parental 15-mer peptides and responders used in the mapping studies. (PDF)

S3 Table Epitope prediction. (PDF)
