# Supplementary material for: Novel, in-natural-infection subdominant HIV-1 CD8+ T-cell epitopes revealed in human recipients of conserved-region T-cell vaccines
Source: PLoS One. 2017 Apr 27;12(4):e0176418. doi: 10.1371/journal.pone.0176418 (PMC5407754; doi:10.1371/journal.pone.0176418)
Supplement: S1 Fig — (A) The box. 15-mer peptide HC031 was recognized by volunteer 421 of the indicated HLA type, and the optimal peptides and determined HLA restriction are summarized. Cryopreserved lymphocytes from the vaccine recipient were expanded by stimulation with 'parental' 15-mer responder peptide for 10 days to establish STCL effector cells. These were subjected to ICS using serially truncated (B), and overlapping 9-mer (C) peptides. In (B), IFN-γ (green) and TNF-α (orange) production and surface expression of CD107a (pink) served as the read-out. Arrows next to an amino acid indicate the peptide-terminal amino acid residue required for efficient peptide recognition. (PDF) [file pone.0176418.s001.pdf]

A

HC031 CTERQANFLGKIWPS (Gag)

VID 421 - A*02:01 (A02) A*11:01 (A03) B*35:03 (B07) B*40:02 (B44) C*02:02 C*12:03

TERQANFL/HLA-B*40:02	Predicted, reported, already on 'A-list'
TERQANFLGK	Not predicted, not reported
CTERQANFLGK	Predicted A*11:01, not reported

B

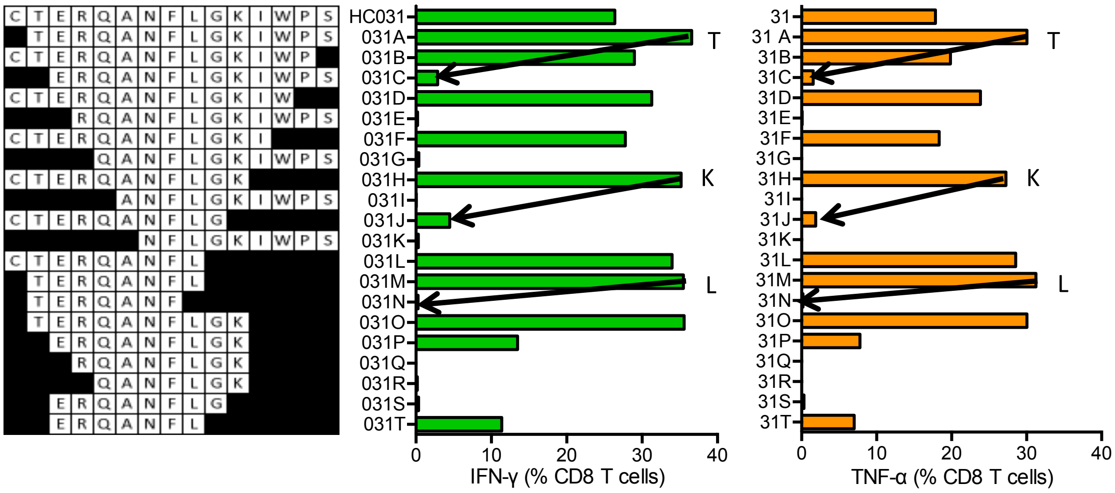

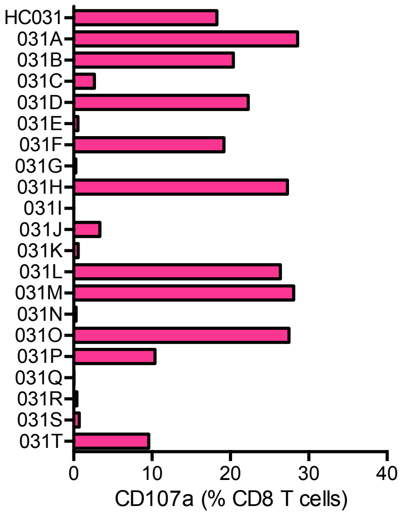

C

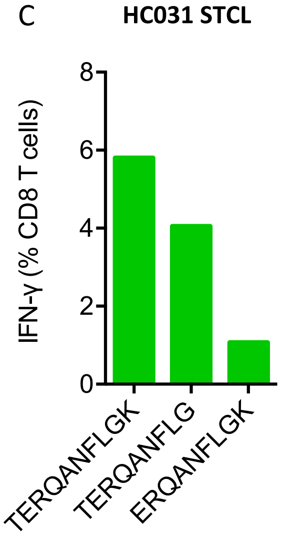

S1 Fig. HC031 CTERQANFLGKIWPS (Gag) - Definition of CD8⁺ T-cell determinants. (A) The box. 15-mer peptide HC031 was recognized by volunteer 421 of the indicated HLA type, and the optimal peptides and determined HLA restriction are summarized. Cryopreserved lymphocytes from the vaccine recipient were expanded by stimulation with 'parental' 15-mer responder peptide for 10 days to establish STCL effector cells. These were subjected to ICS using serially truncated (B), and overlapping 9-mer (C) peptides. In (B), IFN-γ (green) and TNF-α (orange) production and surface expression of CD107a (pink) served as the read-out. Arrows next to an amino acid indicate the peptide-terminal amino acid residue required for efficient peptide recognition.
