# Supplementary material for: Novel, in-natural-infection subdominant HIV-1 CD8+ T-cell epitopes revealed in human recipients of conserved-region T-cell vaccines
Source: PLoS One. 2017 Apr 27;12(4):e0176418. doi: 10.1371/journal.pone.0176418 (PMC5407754; doi:10.1371/journal.pone.0176418)
Supplement: S2 Fig — (A) The box. Overlapping 'parental' peptides HC049 and HC050 were recognized by volunteer 406 of the indicated HLA type. Optimal peptide and its HLA restriction are shown. (B) The subject’s cryopreserved lymphocytes were expanded by stimulation with the 'parental' responder peptide for 10 days to establish STCL effector cells, which were tested in ICS using serially truncated peptides, whereby IFN-γ (green) and TNF-α (orange) production and surface expression of CD107a (pink) were detected. Arrows with an amino acid on the horizontal-bar graphs indicate the peptide-terminal amino acid residue required for efficient peptide recognition. (PDF) [file pone.0176418.s002.pdf]

A

HC049 KNFPISPIETVPVKLK / HC050 SPIETVPVKLKPGMD (Pol)

VID 406 - A*03:01 (A03) A*31:01 (A03) B*40:01 (B44) B*44:03 (B44) C*03:04 C*04:01

IETVPVKL/HLA-B*40:01 Predicted, reported, already on 'A-list'

B

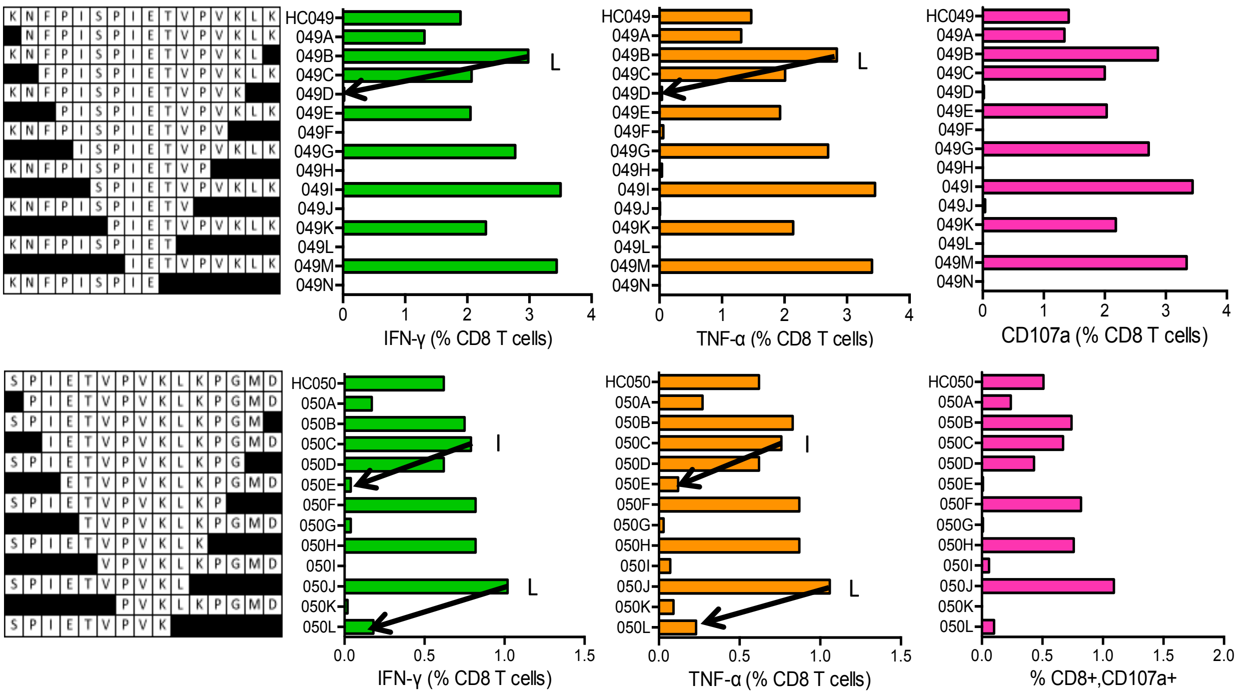

S2 Fig. HC049 KNFPISPIETVPVKLK / HC050 SPIETVPVKLKPGMD (Pol) - Definition of CD8[+] T-cell determinants. (A) The box. Overlapping 'parental' peptides HC049 and HC050 were recognized by volunteer 406 of the indicated HLA type. Optimal peptide and its HLA restriction are shown. (B) The subject’s cryopreserved lymphocytes were expanded by stimulation with the 'parental' responder peptide for 10 days to establish STCL effector cells, which were tested in ICS using serially truncated peptides, whereby IFN-γ (green) and TNF-α (orange) production and surface expression of CD107a (pink) were detected. Arrows with an amino acid on the horizontal-bar graphs indicate the peptide-terminal amino acid residue required for efficient peptide recognition.
