# Supplementary material for: Novel, in-natural-infection subdominant HIV-1 CD8+ T-cell epitopes revealed in human recipients of conserved-region T-cell vaccines
Source: PLoS One. 2017 Apr 27;12(4):e0176418. doi: 10.1371/journal.pone.0176418 (PMC5407754; doi:10.1371/journal.pone.0176418)
Supplement: S3 Fig — (A) The box. 15-mer peptide HC078 was recognized by volunteer 421 of the indicated HLA type. Optimal peptides and their HLA restriction are summarized. (B) Cryopreserved lymphocytes were expanded by stimulation with 'parental' 15-mer responder peptide for 10 days to establish STCL effector cells. These were subjected to ICS using serially truncated peptides monitoring IFN-γ (green) and TNF-α (orange) production and surface expression of CD107a (pink). Arrows next to an amino acid indicate the peptide-terminal amino acid residue required for efficient peptide recognition. (C) The same SCTLs were stimulated using overlapping 9-mer peptide-pulsed 721.221 cells transfected with either HLA-A*11:01 (left) or HLA-C*12:03 (right). (PDF) [file pone.0176418.s003.pdf]

A

HC0078 YFSVPLDEGFRKYTA (Pol)

VID 421 - A*02:01 (A02) A*11:01 (A03) B*35:03 (B07) B*40:02 (B44) C*02:02 C*12:03

SVPLDEGFRK/HLA-A*11:01 Predicted, reported as A11, confirmed as A*11:01**, 'A-list' candidate**

VPLDEGFRK/HLA-C*12:03 Not predicted, not reported, confirmed as C*12:03, **'A-list' candidate**

B

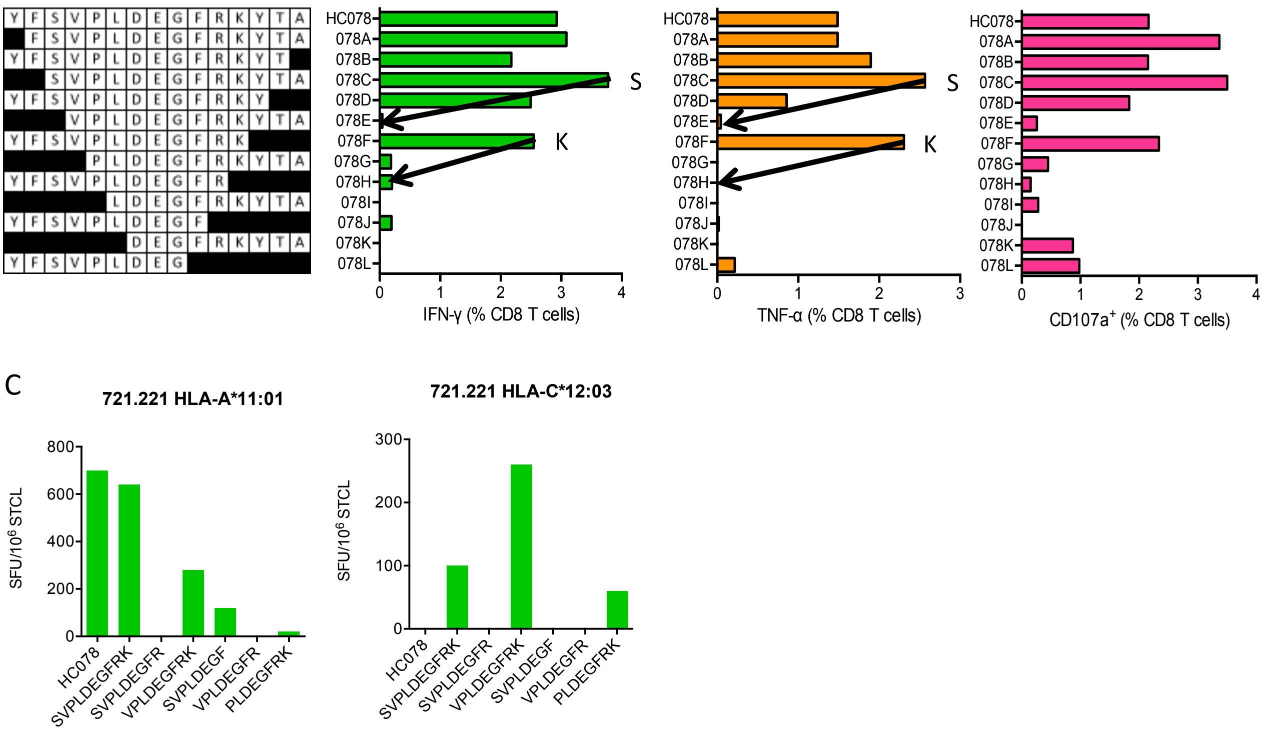

S3 Fig. HC0078 YFSVPLDEGFRKYTA (Pol) - Definition of CD8^{+} T-cell determinants. (A) The box. 15-mer peptide HC078 was recognized by volunteer 421 of the indicated HLA type. Optimal peptides and their HLA restriction are summarized. (B) Cryopreserved lymphocytes were expanded by stimulation with 'parental' 15-mer responder peptide for 10 days to establish STCL effector cells. These were subjected to ICS using serially truncated peptides monitoring IFN-γ (green) and TNF-α (orange) production and surface expression of CD107a (pink). Arrows next to an amino acid indicate the peptide-terminal amino acid residue required for efficient peptide recognition. (C) The same SCTLs were stimulated using overlapping 9-mer peptide-pulsed 721.221 cells transfected with either HLA-A*11:01 (left) or HLA-C*12:03 (right).
