# Supplementary material for: Novel, in-natural-infection subdominant HIV-1 CD8+ T-cell epitopes revealed in human recipients of conserved-region T-cell vaccines
Source: PLoS One. 2017 Apr 27;12(4):e0176418. doi: 10.1371/journal.pone.0176418 (PMC5407754; doi:10.1371/journal.pone.0176418)
Supplement: S4 Fig — (A) The box. 15-mer peptides HC078 and HC079 were recognized by volunteer 417 of the indicated HLA type. Optimal peptide and its HLA restriction are shown. Cryopreserved lymphocytes were expanded by stimulation with 'parental' 15-mer responder peptide for 10 days to establish an STCL, which was subjected to ICS using serially truncated (B), and overlapping 9-mer or 8-mer (C) peptides. IFN-γ (green) and TNF-α (orange) production and surface expression of CD107a (pink) served as the read-out. Arrows next to an amino acid indicate the peptide-terminal amino acid residue required for efficient peptide recognition. (D) 721.221 cells expressing individual HLA class I alleles of VID 417 were utilized for determination of HLA restriction for peptides PLDEGFRKY. (E) Peptides PLDEGFRKY and VPLDEGFRKY were tested side-by-side on HLA-B*35:01-transfected 721.221 cells for optimal stimulation of HC078-expanded SCTL. (PDF) [file pone.0176418.s004.pdf]

A

HC078 YFSVPLDEGFRKYTA / HC079 PLDEGFRKYTAFTIP (Pol)

VID 417 - A*03:01 (A03) A*30:04 (A01) B*35:01 (B07) B*50:01 (B44) C*04:01 C*06:02

VPLDEGFRKY/HLA-B*35:01 Predicted, reported, confirmed as B*35:01, already on 'A-list'

B

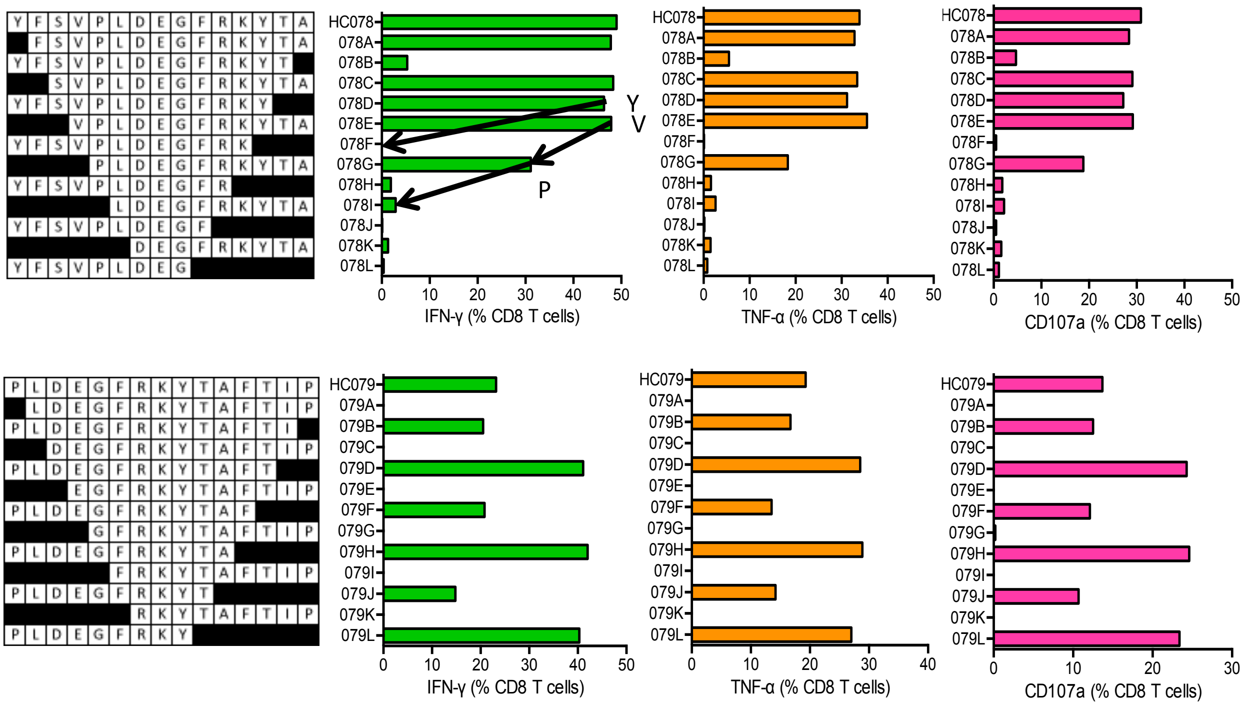

C

HC078 STCL response to 9-mers

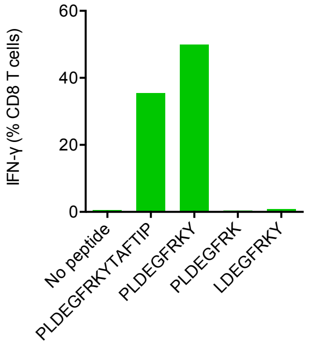

There was no HC078 STCL response to 8-mers

D VID 417 - HLA-B*35:01 restriction for PY9 by HC078 STCL on HLA-transfected 721.221 cells

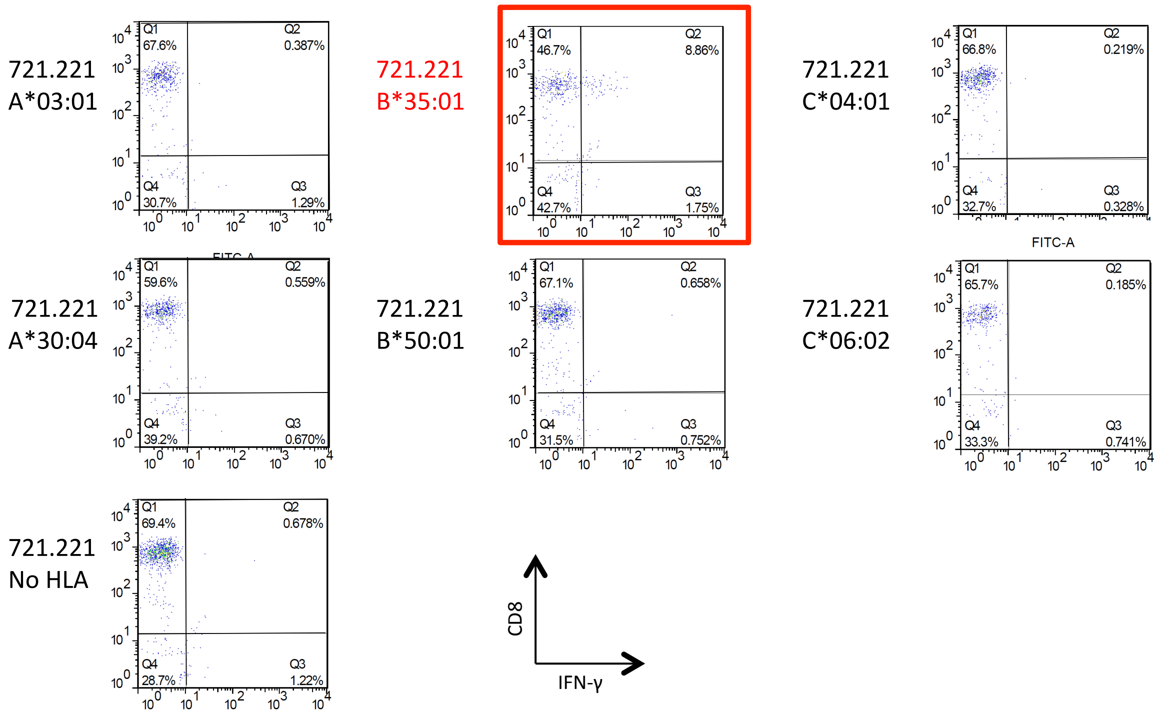

E VID 417 – VY10 is the optimal epitope recognized by HC078 STCL on HLA-B*35:01-transfected 721.221 cells

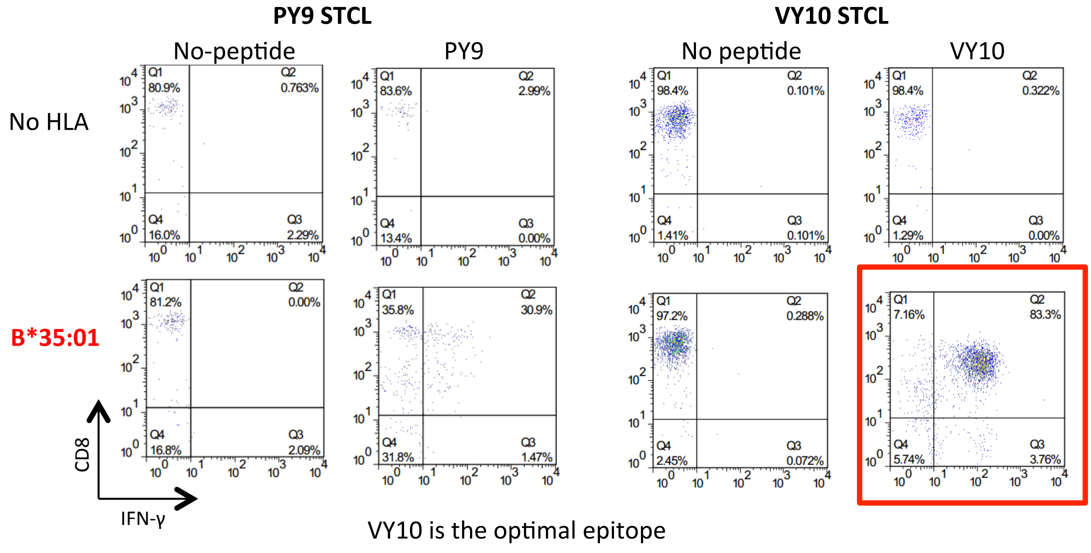

S4 Fig. HC078 YFSVPLDEGFRKYTA / HC079 PLDEGFRKYTAFTIP (Pol) - Definition of $CD8^+$ T-cell determinants. (A) The box. 15-mer peptides HC078 and HC079 were recognized by volunteer 417 of the indicated HLA type. Optimal peptide and its HLA restriction are shown. Cryopreserved lymphocytes were expanded by stimulation with 'parental' 15-mer responder peptide for 10 days to establish an STCL, which was subjected to ICS using serially truncated (B), and overlapping 9-mer or 8-mer (C) peptides. IFN-γ (green) and TNF-α (orange) production and surface expression of CD107a (pink) served as the read-out. Arrows next to an amino acid indicate the peptide-terminal amino acid residue required for efficient peptide recognition. (D) 721.221 cells expressing individual HLA class I alleles of VID 417 were utilized for determination of HLA restriction for peptides PLDEGFRKY. (E) Peptides PLDEGFRKY and VPLDEGFRKY were tested side-by-side on HLA-B*35:01-transfected 721.221 cells for optimal stimulation of HC078-expanded SCTL.
