# Supplementary material for: Novel, in-natural-infection subdominant HIV-1 CD8+ T-cell epitopes revealed in human recipients of conserved-region T-cell vaccines
Source: PLoS One. 2017 Apr 27;12(4):e0176418. doi: 10.1371/journal.pone.0176418 (PMC5407754; doi:10.1371/journal.pone.0176418)
Supplement: S5 Fig — (A) The box. 15-mer peptide HC080 was recognized by volunteer 411 of the indicated HLA type. Optimal peptides are listed below. Cryopreserved 411 lymphocytes were expanded by stimulation with 'parental' 15-mer peptide for 10 days to establish STCL effector cells. These were subjected to ICS using serially truncated (B), and overlapping 9-mer or 8-mer (C) peptides monitoring IFN-γ (green) and TNF-α (orange) production and surface expression of CD107a (pink). In (B), arrows next to an amino acid indicate the peptide-terminal amino acid residue required for efficient peptide recognition. (D) Three strong stimulatory peptides were subjected to titration against the HC080-expanded SCTL. (PDF) [file pone.0176418.s005.pdf]

A

HC080 GFRKYTAFTIPSINN (Pol)

VID 411 - A*02:01 (A02) A*02:01 (A02) B*08:01 (B08) B*51:01 (B07) C*03:03 C*07:01

**TAFTIPSI**	Predicted B*51:01 and C*03:03, reported A*02:01 and B*51:01
**YTAFTIPSI**	Predicted A*02:01 and B*51:01, reported A*02:01
**KYTAFTIPSI**	Predicted C*07:01, reported A2

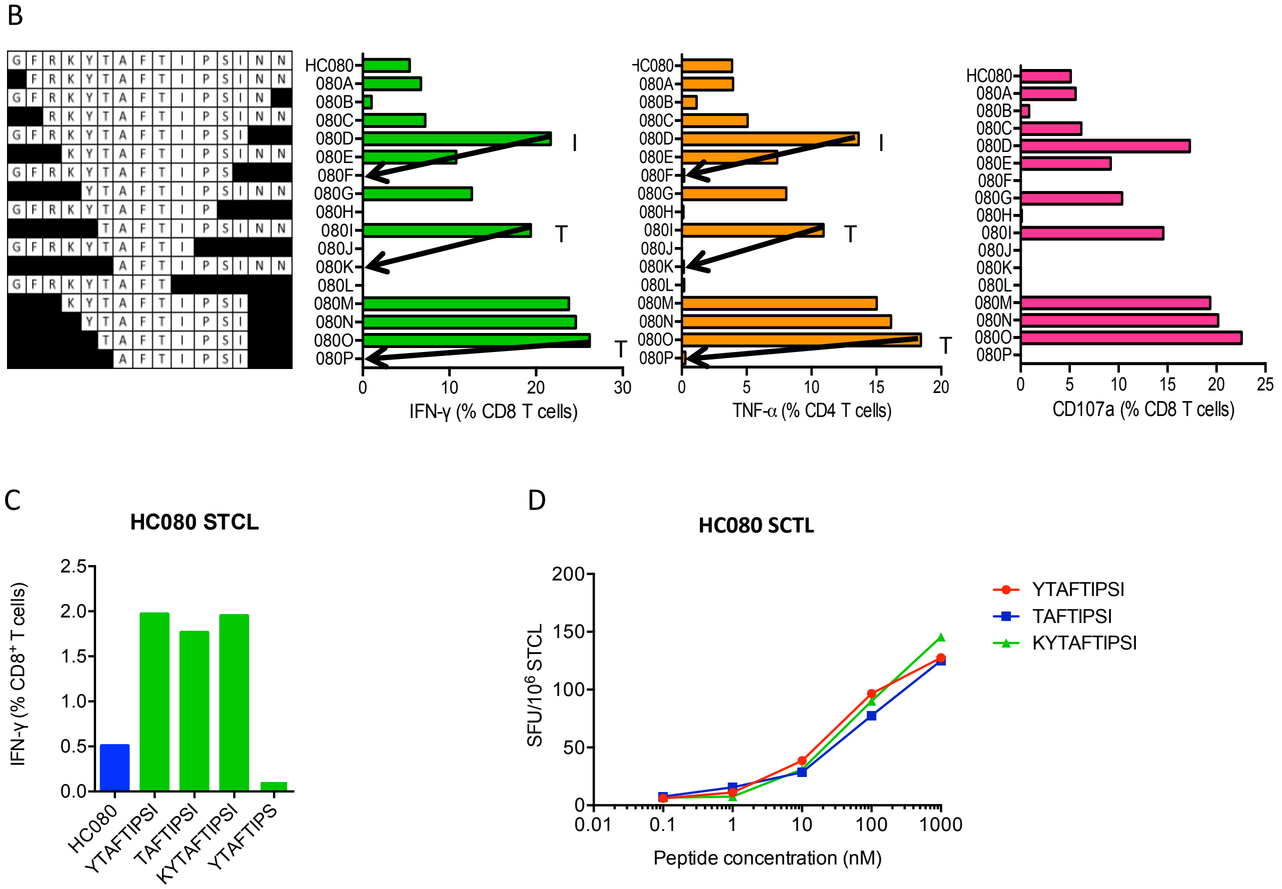

S5 Fig. HC080 GFRKYTAFTIPSINN (Pol) - Definition of CD8^{+} T-cell determinants. (A) The box. 15-mer peptide HC080 was recognized by volunteer 411 of the indicated HLA type. Optimal peptides are listed below. Cryopreserved 411 lymphocytes were expanded by stimulation with 'parental' 15-mer peptide for 10 days to establish STCL effector cells. These were subjected to ICS using serially truncated (B), and overlapping 9-mer or 8-mer (C) peptides monitoring IFN-γ (green) and TNF-α (orange) production and surface expression of CD107a (pink). In (B), arrows next to an amino acid indicate the peptide-terminal amino acid residue required for efficient peptide recognition. (D) Three strong stimulatory peptides were subjected to titration against the HC080-expanded SCTL.
