# Supplementary material for: Novel, in-natural-infection subdominant HIV-1 CD8+ T-cell epitopes revealed in human recipients of conserved-region T-cell vaccines
Source: PLoS One. 2017 Apr 27;12(4):e0176418. doi: 10.1371/journal.pone.0176418 (PMC5407754; doi:10.1371/journal.pone.0176418)
Supplement: S6 Fig — (A) The box. 15-mer peptide HC081 was recognized by volunteer 415 of the indicated HLA type. While restricting HLAs were not determined for the three stimulatory peptides, the HLA-A*02:01 restriction for the ‘parental’-derived peptide was confirmed. Volunteer’s lymphocytes were expanded by stimulation with peptide HC081 for 10 days to establish an STCL, which was subjected to ICS using serially truncated (B), and overlapping 9-mer (C) peptides. IFN-γ (green) and TNF-α (orange) production and surface expression of CD107a (pink) served as the read-out. Arrows next to an amino acid indicate the peptide-terminal amino acid residue required for efficient peptide recognition. (D) 721.221 and C1R cells expressing individual HLA class I alleles of volunteer 415 were utilized to determine the HLA restriction of ‘parental’ peptide HC081. (PDF) [file pone.0176418.s006.pdf]

A

HC081 YTAFTIPSINNETPG (Pol)

VID 415 - A*02:01 (A02) A*03:01 (A03) B*07:02 (B07) B*44:02 (B44) C*07:02 C*07:02

TIPSINNETPG	Not predicted, not reported
PSINNETPG	Not predicted, not reported
YTAFTIPSI	Predicted A*02:01 and C*07:02, reported A2
YTAFTIPSINNETPG	Confirmed A*02:01

B

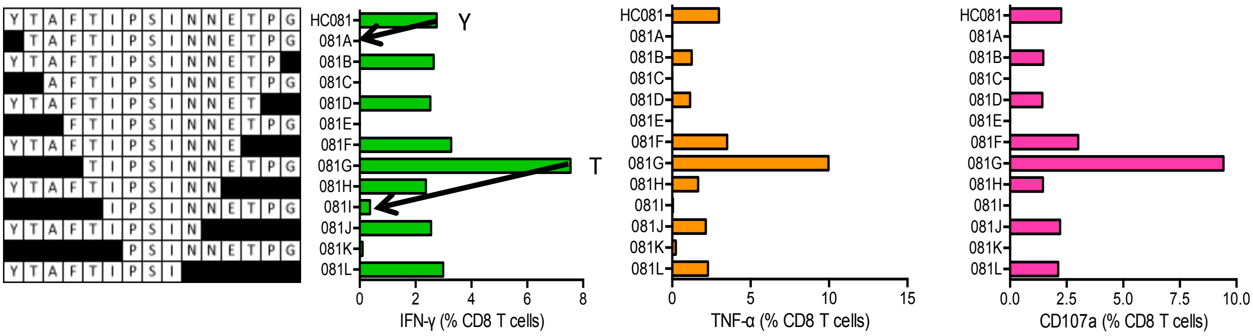

C

HC081 STCL

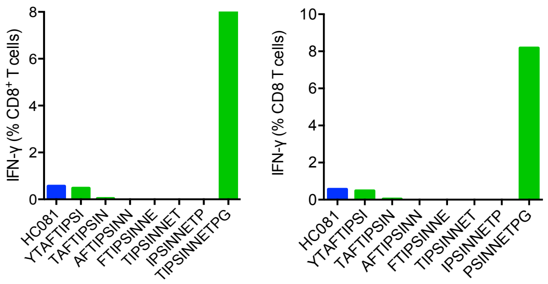

D **VID 415 - HLA-A*02:01 is the restriction for HC081 by HC081 STCL on HLA-transfected 721.221 or C1R cells**

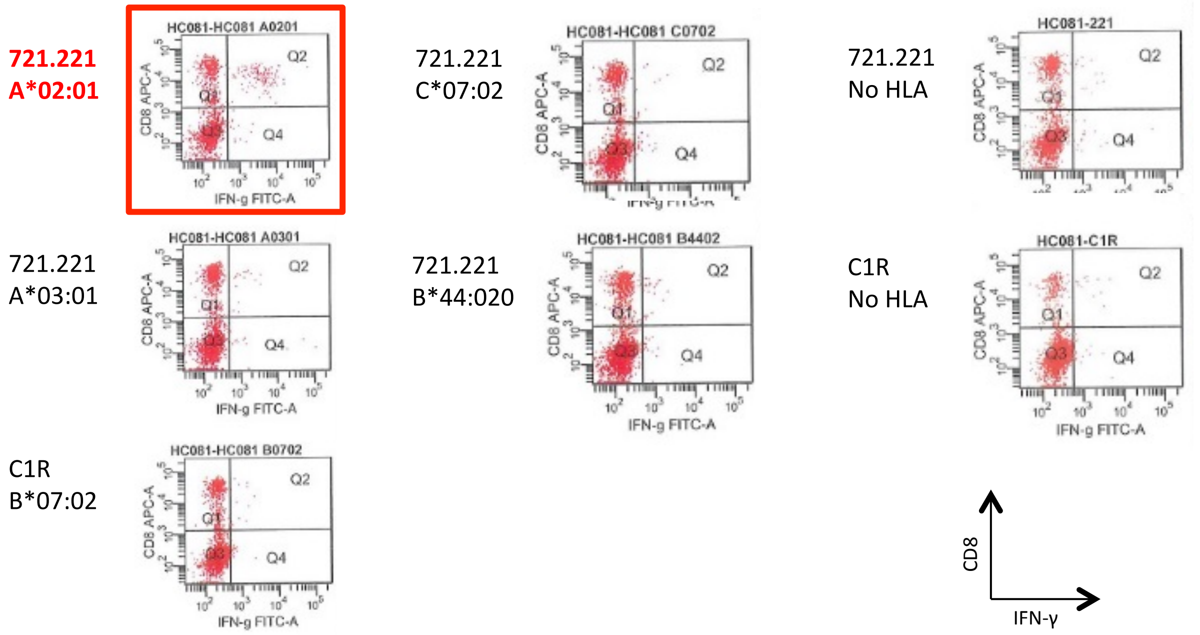

S6 Fig. HC081 YTAFTIPSINNETPG (Pol) - Definition of CD8⁺ T-cell determinants. (A) The box. 15-mer peptide HC081 was recognized by volunteer 415 of the indicated HLA type. While restricting HLAs were not determined for the three stimulatory peptides, the HLA-A*02:01 restriction for the 'parental'-derived peptide was confirmed. Volunteer's lymphocytes were expanded by stimulation with peptide HC081 for 10 days to establish an STCL, which was subjected to ICS using serially truncated (B), and overlapping 9-mer (C) peptides. IFN-γ (green) and TNF-α (orange) production and surface expression of CD107a (pink) served as the read-out. Arrows next to an amino acid indicate the peptide-terminal amino acid residue required for efficient peptide recognition. (D) 721.221 and C1R cells expressing individual HLA class I alleles of volunteer 415 were utilized to determine the HLA restriction of 'parental' peptide HC081.
