# Supplementary material for: Novel, in-natural-infection subdominant HIV-1 CD8+ T-cell epitopes revealed in human recipients of conserved-region T-cell vaccines
Source: PLoS One. 2017 Apr 27;12(4):e0176418. doi: 10.1371/journal.pone.0176418 (PMC5407754; doi:10.1371/journal.pone.0176418)
Supplement: S7 Fig — (A) The box. 15-mer peptide HC088 was recognized by volunteer 409 of the shown HLA type. Optimal peptides and determined HLA restriction are summarized below. Volunteer’s lymphocytes were expanded by stimulation with peptide HC088 for 10 days to establish an STCL, which was subjected to ICS using serially truncated (B), and overlapping 9-mer (C) peptides monitoring IFN-γ (green) and TNF-α (orange) production and surface expression of CD107a (pink). In (B), arrows next to an amino acid indicate the peptide-terminal amino acid residue required for efficient peptide recognition. (D) 721.221 cells expressing the HLA-B*07:02 and HLA-B*08:01 alleles were used to determine the HLA restriction of peptide SPAIFQSSMTK. (PDF) [file pone.0176418.s007.pdf]

A

HC088 GSPAIFQSSMTKILE (Pol)

VID 409 - A*01:01 (A01) A*03:01 (A03) <u>B*07:02</u> (B07) B*08:01 (B08) C*07:01 C*07:02

**SPAIFQSSMTK/HLA-B*07:02**	Not predicted, reported B7, confirmed B*07:02, **'A-list' candidate**
**IFQSSMTKIL**	Not predicted, not present

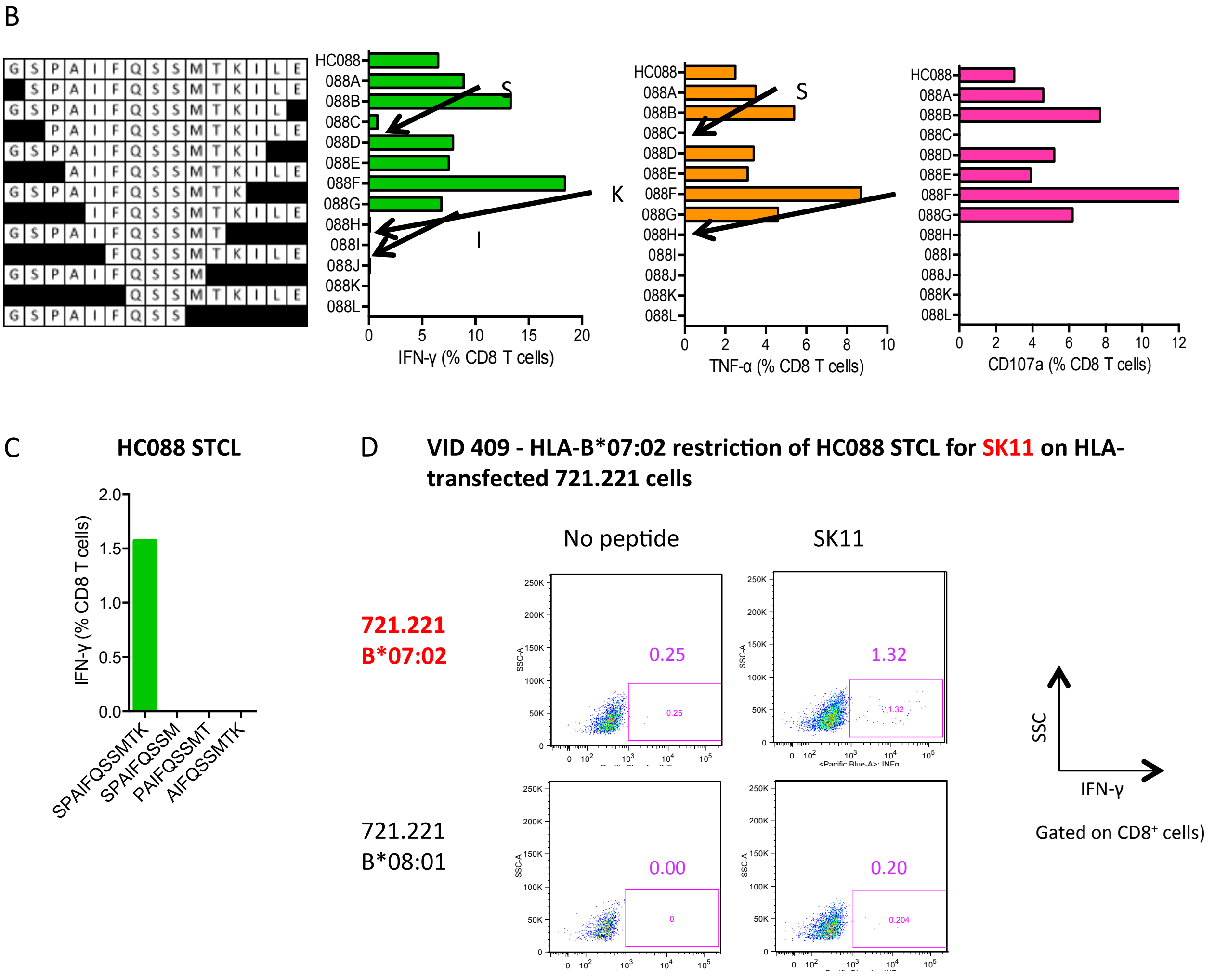

S7 Fig. HC088 GSPAIFQSSMTKILE (Pol) - Definition of CD8⁺ T-cell determinants. (A) The box. 15-mer peptide HC088 was recognized by volunteer 409 of the shown HLA type. Optimal peptides and determined HLA restriction are summarized below. Volunteer's lymphocytes were expanded by stimulation with peptide HC088 for 10 days to establish an STCL, which was subjected to ICS using serially truncated (B), and overlapping 9-mer (C) peptides monitoring IFN-γ (green) and TNF-α (orange) production and surface expression of CD107a (pink). In (B), arrows next to an amino acid indicate the peptide-terminal amino acid residue required for efficient peptide recognition. (D) 721.221 cells expressing the HLA-B*07:02 and HLA-B*08:01 alleles were used to determine the HLA restriction of peptide SPAIFQSSMTK.
