# Supplementary material for: Novel, in-natural-infection subdominant HIV-1 CD8+ T-cell epitopes revealed in human recipients of conserved-region T-cell vaccines
Source: PLoS One. 2017 Apr 27;12(4):e0176418. doi: 10.1371/journal.pone.0176418 (PMC5407754; doi:10.1371/journal.pone.0176418)
Supplement: S9 Fig — (A) The box. 15-mer peptide HC091 was recognized by volunteer 410 of the shown HLA type. Optimal peptides and known restricting HLA allele are listed below. Cryopreserved 410 lymphocytes were expanded by stimulation with the 'parental' 15-mer peptide for 10 days to establish STCL effector cells. These were subjected to ICS using serially truncated (B), and overlapping 9-mer or 8-mer (C) peptides monitoring IFN-γ (green) and TNF-α (orange) production and surface expression of CD107a (pink). (D) Peptides were also subjected to titration against the HC091-expanded SCTL. (PDF) [file pone.0176418.s009.pdf]

A

HC091 ILEPFRAQNPEIVIY (Pol)

VID 410 - A*30:02 (A01) <u>A*30:02</u> (A01) B*18:02 (B27) B*57:03 (B58) C*07:01 C*18:01

**FRAQNPEIVIY**	Not predicted, not reported
**RAQNPEIVIY**	Predicted A*30:02, not reported
**AQNPEIVIY/HLA-A*30:02**	Predicted A*30:02, reported A*30:02:01

B

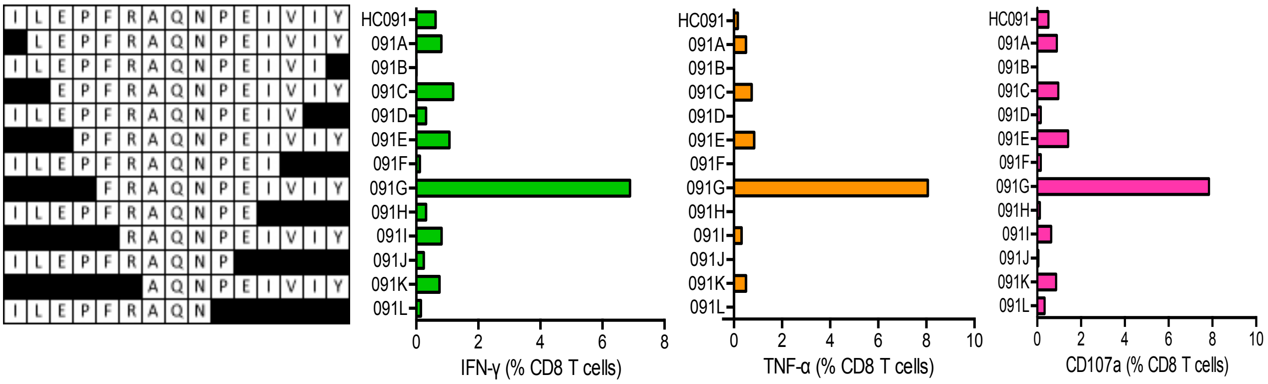

C

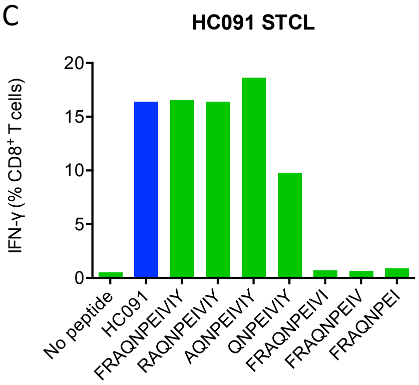

D

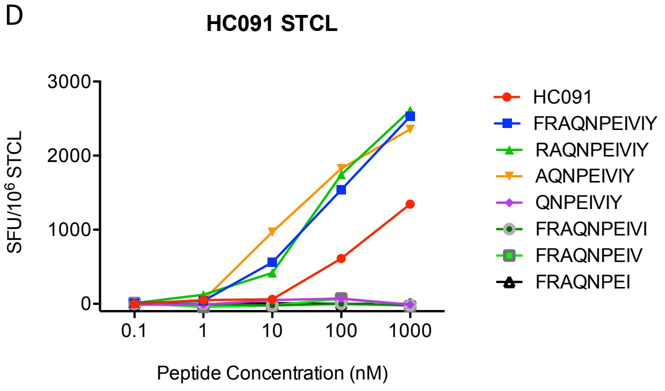

S9 Fig. HC091 ILEPFRAQNPEIVIY (Pol) - Definition of CD8⁺ T-cell determinants. (A) The box. 15-mer peptide HC091 was recognized by volunteer 410 of the shown HLA type. Optimal peptides and known restricting HLA allele are listed below. Cryopreserved 410 lymphocytes were expanded by stimulation with the 'parental' 15-mer peptide for 10 days to establish STCL effector cells. These were subjected to ICS using serially truncated (B), and overlapping 9-mer or 8-mer (C) peptides monitoring IFN-γ (green) and TNF-α (orange) production and surface expression of CD107a (pink). (D) Peptides were also subjected to titration against the HC091-expanded SCTL.
