# Supplementary material for: Novel, in-natural-infection subdominant HIV-1 CD8+ T-cell epitopes revealed in human recipients of conserved-region T-cell vaccines
Source: PLoS One. 2017 Apr 27;12(4):e0176418. doi: 10.1371/journal.pone.0176418 (PMC5407754; doi:10.1371/journal.pone.0176418)
Supplement: S10 Fig — (A) The box. Overlapping peptides HC092 and HC093 were recognized by volunteer 410 of the indicated HLA type. Optimal peptides and determined HLA restriction are shown. Volunteer’s lymphocytes were expanded by stimulation with peptide ‘parental peptides for 10 days to establish STCLs, which were subjected to ICS using serially truncated (B), and overlapping 9-mer (C) peptides monitoring IFN-γ (green) and TNF-α (orange) production and surface expression of CD107a (pink). In (B), arrows next to an amino acid indicate the peptide-terminal amino acid residue required for efficient peptide recognition. (D) 721.221 cells expressing the volunteer’s HLA alleles were used to determine the HLA restriction of peptide EIVIYQYMD. (PDF) [file pone.0176418.s010.pdf]

A

HC092 FRAQNPEIVIYQYMDKK / HC093 KNPEIVIYQYMDDLYV (Pol) (K added for solubility)

VID 410 - A*30:02 (A01) A*30:02 (A01) B*18:01 (B27) B*57:03 (B58) C*07:01 C*18:01

NPEIVIYQY Predicted B*18:02, reported B18, not confirmed

EIVIYQYMD/HLA-B*18:01 Not predicted, not reported, confirmed B*18:01, **'A-list' candidate**

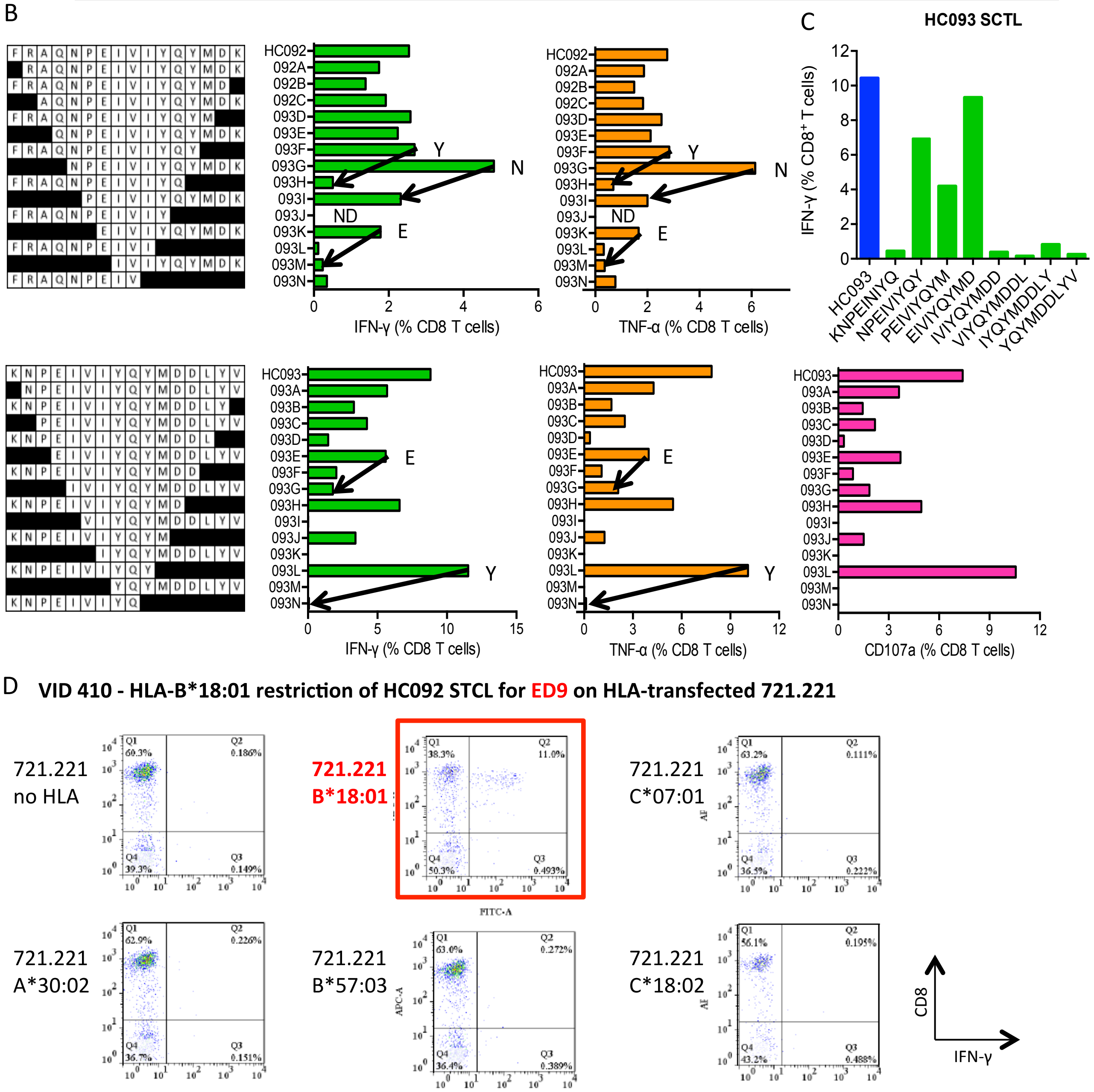

S10 Fig. HC092 FRAQNPEIVIYQYMDKK / HC093 KNPEIVIYQYMDDLYV (Pol) - Definition of CD8⁺ T-cell determinants. (A) The box. Overlapping peptides HC092 and HC093 were recognized by volunteer 410 of the indicated HLA type. Optimal peptides and determined HLA restriction are shown. Volunteer's lymphocytes were expanded by stimulation with peptide 'parental peptides for 10 days to establish STCLs, which were subjected to ICS using serially truncated (B), and overlapping 9-mer (C) peptides monitoring IFN-γ (green) and TNF-α (orange) production and surface expression of CD107a (pink). In (B), arrows next to an amino acid indicate the peptide-terminal amino acid residue required for efficient peptide recognition. (D) 721.221 cells expressing the volunteer's HLA alleles were used to determine the HLA restriction of peptide EIVIYQYMD.
