# Supplementary material for: Novel, in-natural-infection subdominant HIV-1 CD8+ T-cell epitopes revealed in human recipients of conserved-region T-cell vaccines
Source: PLoS One. 2017 Apr 27;12(4):e0176418. doi: 10.1371/journal.pone.0176418 (PMC5407754; doi:10.1371/journal.pone.0176418)
Supplement: S11 Fig — (A) The box. Peptide HC093 was recognized by volunteers 411, 415, 416 and 418 of the indicated HLA types. Stimulatory HLA-A*02:01 peptides are listed below. (B) Volunteers’ lymphocytes were expanded using the HC093 peptide for 10 days to establish STCLs, which were subjected to ICS using serially truncated and overlapping 9-mer or 8-mer peptides monitoring IFN-γ (green) and TNF-α (orange) production and surface expression of CD107a (pink). Arrows next to an amino acid indicate the peptide-terminal amino acid residue required for efficient peptide recognition. (C) 418’s STCL were tested for recognition of epitope variants. (D) 721.221 cells expressing HLA alleles of volunteer 416 were used to determine the HLA restriction of peptide QYMDDLYV. (E) STCLs of volunteer 416 were expanded using one of the four PV11, IV10, YV9 or QV8 peptides and tested against decreasing concentrations of all four peptides. (PDF) [file pone.0176418.s011.pdf]

A

HC093 KNPEIVIYQYMDDLYV (Pol) (K added for solubility)

VID 411 - A*02:01 (A02) A*02:01 (A02) B*08:01 (B08) B*51:01 (B07) C*03:03 C*07:01

VID 415 - A*02:01 (A02) A*03:01 (A03) B*07:02 (B07) B*44:02 (B44) C*07:02 C*07:02

VID 416 - A*02:01 (A02) A*02:01 (A02) B*08:01 (B08) B*44:02 (B44) C*05:01 C*07:01

VID 418 - A*02:01 (A02) A*24:02 (A24) B*07:02 (B07) B*27:05 (B27) C*01:02 C*07:02

VIYQYMDDLYV/HLA-A*02:01 Predicted A*02:01, reported A*02:01, **'A-list' candidate**

IYQYMDDLYV/HLA-A*02:01 Not predicted A*02:01, reported A*02:01

YQYMDDLYV/HLA-A*02:01 Predicted A*02:01, reported A*02:01, A-list

B

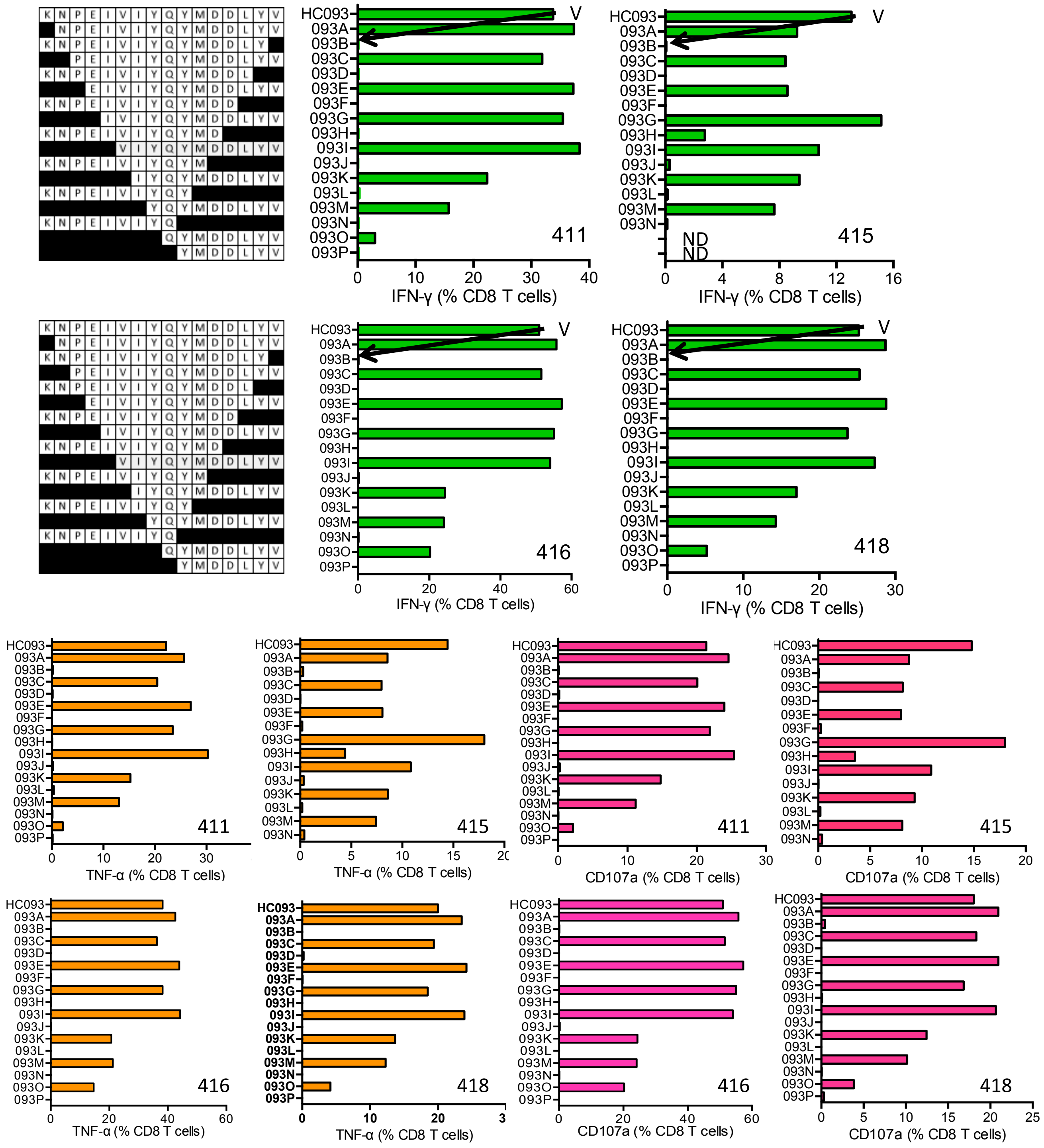

C VID 418 – Depth of peptide recognition by HC093 STCL

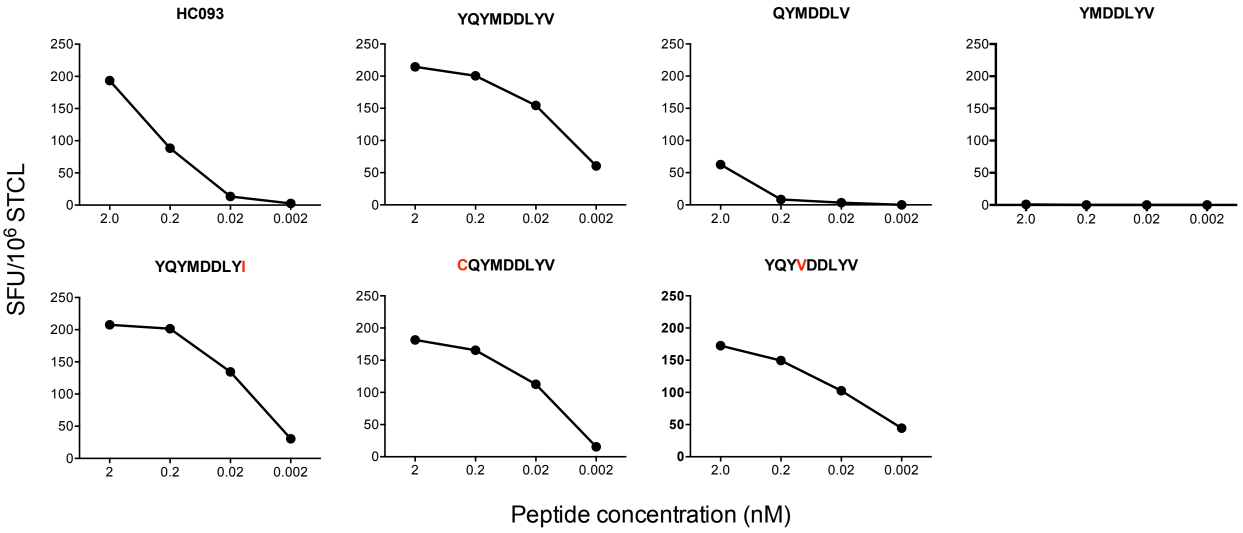

D VID 416 - HLA-A*02:01 restriction of HC093 STCL for QV8 on HLA-transfected 721.221

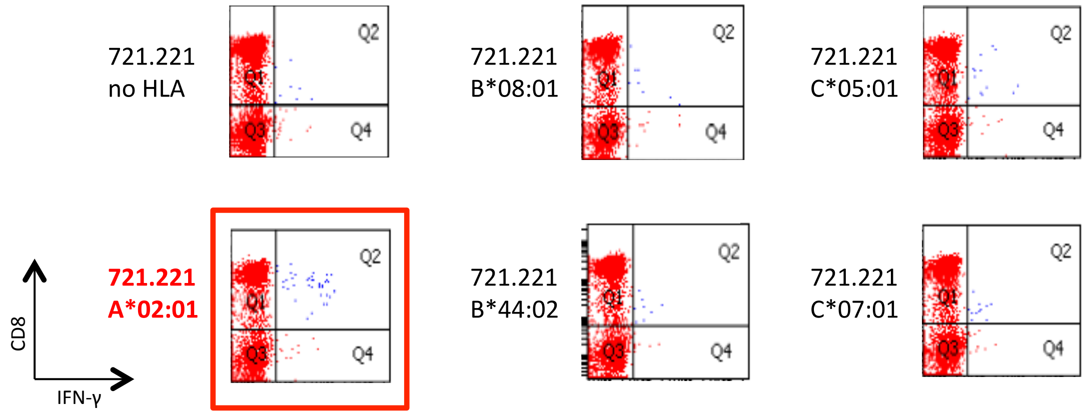

E VID 416 - HLA-A*02:01 restricted VV11- and YV9-specific CD8 T cells were predominant

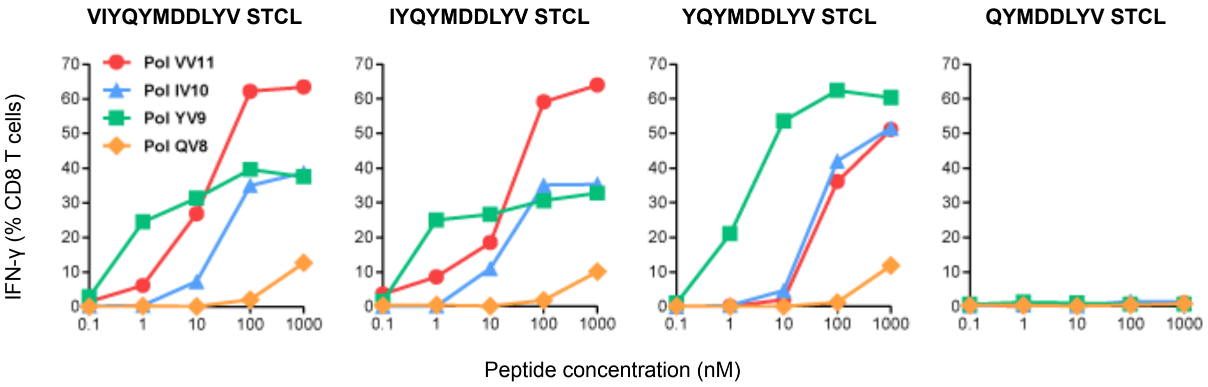

S11 Fig. HC093 KNPEIVIYQYMDDLYV (Pol) - Definition of CD8⁺ T-cell determinants. (A) The box. Peptide HC093 was recognized by volunteers 411, 415, 416 and 418 of the indicated HLA types. Stimulatory HLA-A*02:01 peptides are listed below. (B) Volunteers' lymphocytes were expanded using the HC093 peptide for 10 days to establish STCLs, which were subjected to ICS using serially truncated and overlapping 9-mer or 8-mer peptides monitoring IFN-γ (green) and TNF-α (orange) production and surface expression of CD107a (pink). Arrows next to an amino acid indicate the peptide-terminal amino acid residue required for efficient peptide recognition. (C) 418's STCL were tested for recognition of epitope variants. (D) 721.221 cells expressing HLA alleles of volunteer 416 were used to determine the HLA restriction of peptide QYMDDLYV. (E) STCLs of volunteer 416 were expanded using one of the four PV11, IV10, YV9 or QV8 peptides and tested against decreasing concentrations of all four peptides.
