# Supplementary material for: Novel, in-natural-infection subdominant HIV-1 CD8+ T-cell epitopes revealed in human recipients of conserved-region T-cell vaccines
Source: PLoS One. 2017 Apr 27;12(4):e0176418. doi: 10.1371/journal.pone.0176418 (PMC5407754; doi:10.1371/journal.pone.0176418)
Supplement: S12 Fig — (A) The box. Peptide HC093 was recognized by volunteer 417 of the shown HLA type (HLA-A*02:01-negative). (B) Cryopreserved lymphocytes were expanded with peptide HC093 for 10 days to establish STCL effector cells, which were subjected to ICS using serially truncated peptides monitoring IFN-γ (green) and TNF-α (orange) production and surface expression of CD107a (pink). (PDF) [file pone.0176418.s012.pdf]

A

HC093 KNPEIVIYQYMDDLYV (Pol) (K added for solubility)
VID 417 - A*03:01 (A03) A*30:04 (A01) B*35:01 (B07) B*50:01 (B44) C*04:01 C*06:02
Overlapping epitopes

B

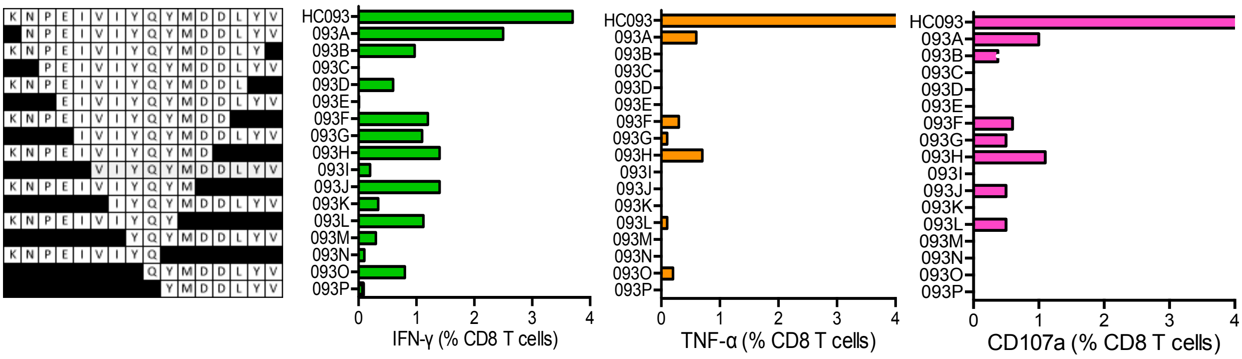

S12 Fig. HC093 KNPEIVIYQYMDDLYV (Pol) - Definition of CD8⁺ T-cell determinants. (A) The box. Peptide HC093 was recognized by volunteer 417 of the shown HLA type (HLA-A*02:01-negative). (B) Cryopreserved lymphocytes were expanded with peptide HC093 for 10 days to establish STCL effector cells, which were subjected to ICS using serially truncated peptides monitoring IFN-γ (green) and TNF-α (orange) production and surface expression of CD107a (pink).
