# Supplementary material for: Novel, in-natural-infection subdominant HIV-1 CD8+ T-cell epitopes revealed in human recipients of conserved-region T-cell vaccines
Source: PLoS One. 2017 Apr 27;12(4):e0176418. doi: 10.1371/journal.pone.0176418 (PMC5407754; doi:10.1371/journal.pone.0176418)

A

HC102 KQVDRMRIRTWKSLVK (Vif) (K added for solubility)

VID 403 - A*01:01 (A01) A*30:01 (A01/03) B*13:02 (ND) B*39:01 (B27) C*06:02 C*07:01

RIRTWKSLVK/HLA-A*30:01 Predicted A*30:01, not reported, confirmed A*30:01, **'A-list' candidate**

IRTWKSLVK/HLA-A*30:01 Not predicted, not reported, confirmed A*30:01, **'A-list' candidate**

B

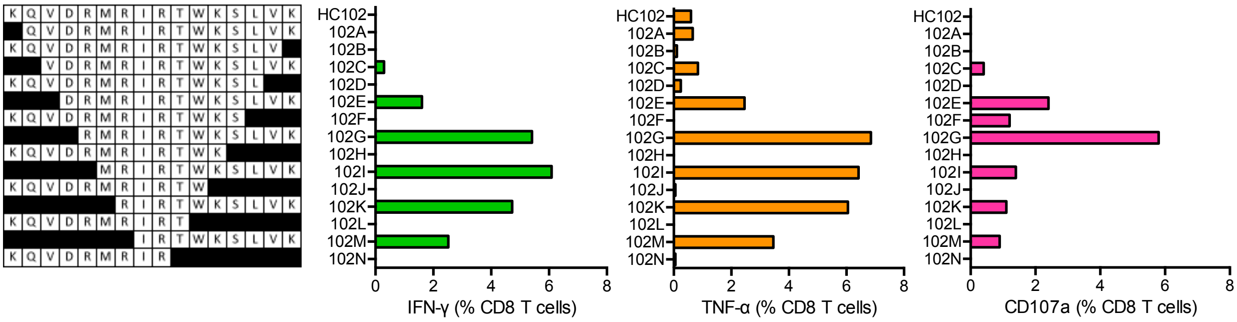

C

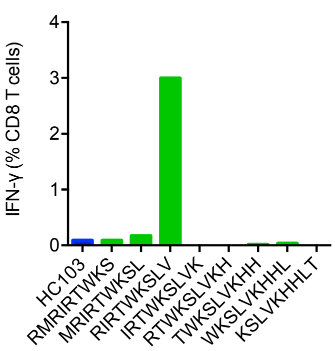

Please see (D) and (E) on the following page.

S13 Fig. HC102 KQVDRMRIRTWKSLVK (Vif) - Definition of $CD8^+$ T-cell determinants. (A) The box. Peptide HC102 was recognized by volunteer 403 of the indicated HLA type and the two optimal peptides and HLA restriction are shown. Cryopreserved lymphocytes from the vaccine recipient were expanded by stimulation with 'parental' responder peptide for 10 days to establish STCL, which was subjected to ICS using serially truncated (B), and overlapping 9-mer (C) peptides. In (B), IFN-γ (green) and TNF-α (orange) production and surface expression of CD107a (pink) served as the read-out. (D) 721.221 and C1R cells expressing all HLA alleles of volunteer 416 were used to determine the HLA restriction of the HC102-derived peptide. (E) HC102-expanded SCTL stimulated with either 721.221 cells transfected with HLA-A*30:01 (top) or just the peptide (bottom) and analysed using flow cytometry.

D VID 403 - HLA-A*30:01 restriction of HC102 STCL for HC102 on HLA-transfected 721.221 or C1R cells

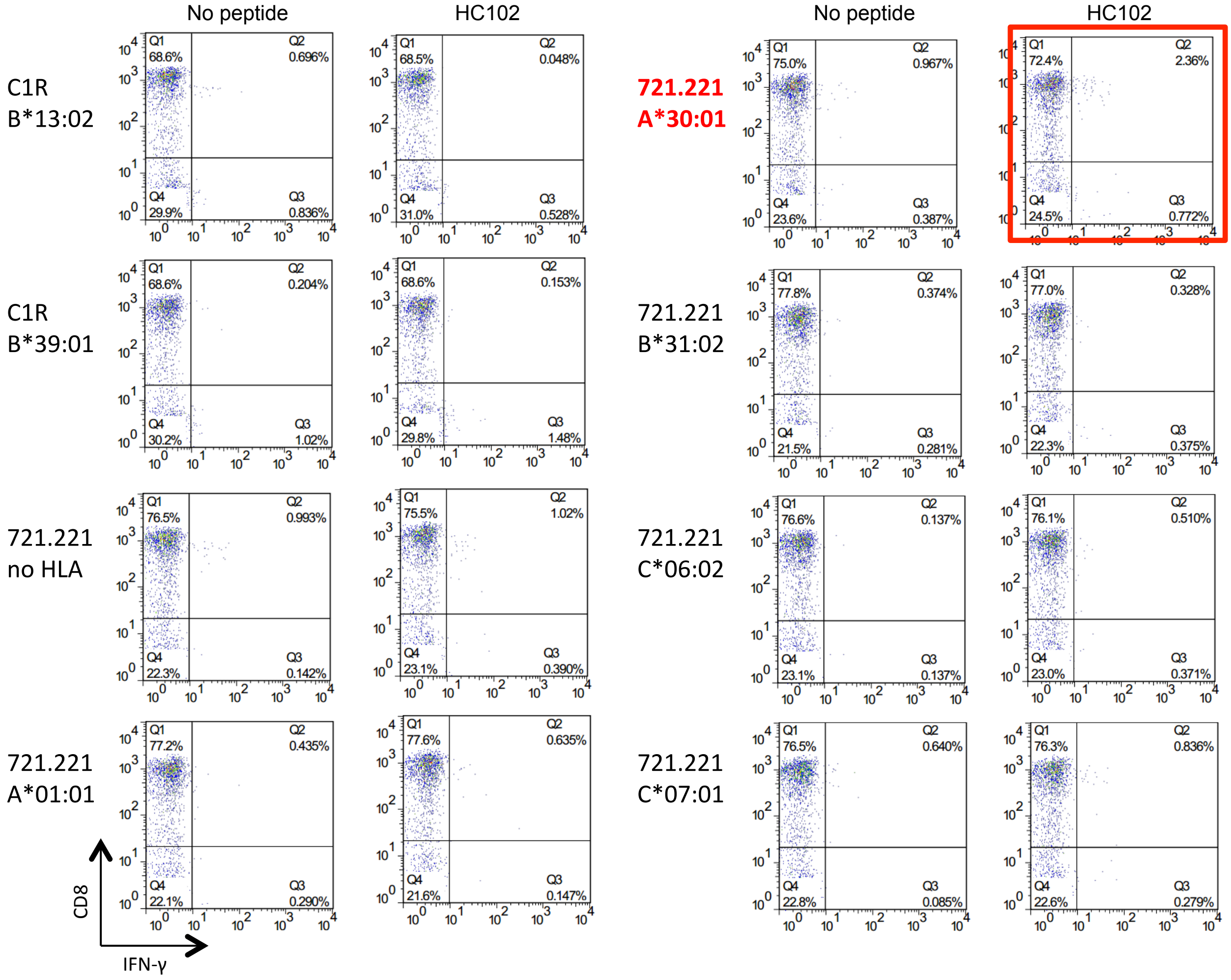

E VID 403 - HLA-A*30:01 restriction of HC102 STCL for RK10 and IK9 on HLA-transfected 721.221 cells

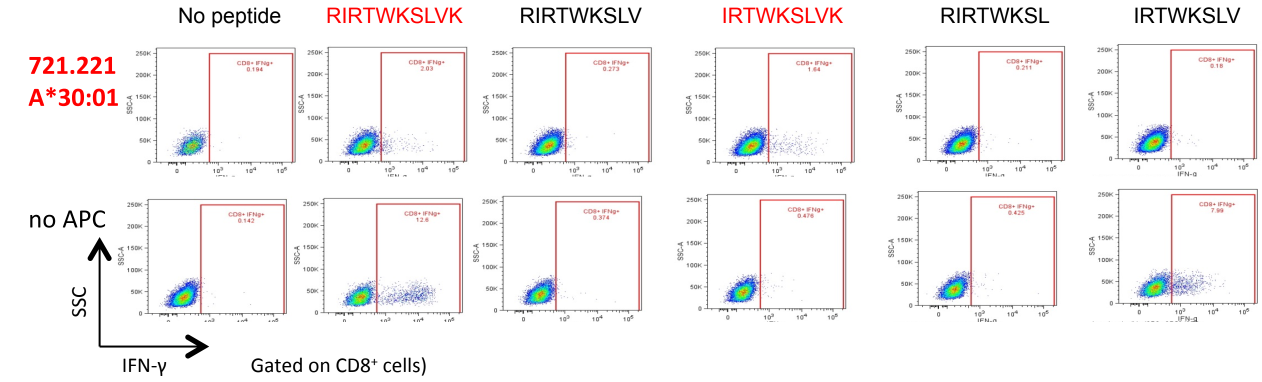

Supplement: S13 Fig — (A) The box. Peptide HC102 was recognized by volunteer 403 of the indicated HLA type and the two optimal peptides and HLA restriction are shown. Cryopreserved lymphocytes from the vaccine recipient were expanded by stimulation with 'parental' responder peptide for 10 days to establish STCL, which was subjected to ICS using serially truncated (B), and overlapping 9-mer (C) peptides. In (B), IFN-γ (green) and TNF-α (orange) production and surface expression of CD107a (pink) served as the read-out. (D) 721.221 and C1R cells expressing all HLA alleles of volunteer 416 were used to determine the HLA restriction of the HC102-derived peptide. (E) HC102-expanded SCTL stimulated with either 721.221 cells transfected with HLA-A*30:01 (top) or just the peptide (bottom) and analysed using flow cytometry. (PDF) [file pone.0176418.s013.pdf]
