# Supplementary material for: Novel, in-natural-infection subdominant HIV-1 CD8+ T-cell epitopes revealed in human recipients of conserved-region T-cell vaccines
Source: PLoS One. 2017 Apr 27;12(4):e0176418. doi: 10.1371/journal.pone.0176418 (PMC5407754; doi:10.1371/journal.pone.0176418)
Supplement: S14 Fig — (A) The box. Peptide HC103 was recognized by volunteer 418 of the indicated HLA type and the optimal peptide and its HLA restriction are shown. '-' indicates junction between two adjacent HIVconsv regions. (B) Cryopreserved lymphocytes from the vaccine recipient were expanded by stimulation with 'parental' peptide for 10 days to establish STCL, which was subjected to ICS using serially truncated peptides. IFN-γ (green) and TNF-α (orange) production and surface expression of CD107a (pink) served as the read-out. Arrows next to an amino acid indicate the peptide-terminal amino acid residue required for efficient peptide recognition. (C) 721.221 and C1R cells expressing all HLA alleles of volunteer 418 were used to determine the HLA restriction of the HC103-derived peptide by restimulation followed by flow cytometry. (PDF) [file pone.0176418.s014.pdf]

A

HC103 MRIRTWKSLVKHH-LT (Vif)

VID 418 - A*02:01 (A02) A*24:02 (A24) B*07:02 (B07) B*27:05 (B27) C*01:02 C*07:02

IRTWKSLVKH/HLA-B*27:05 Predicted B*27:05, not reported, confirmed B*27:05, **'A-list' candidate**

B

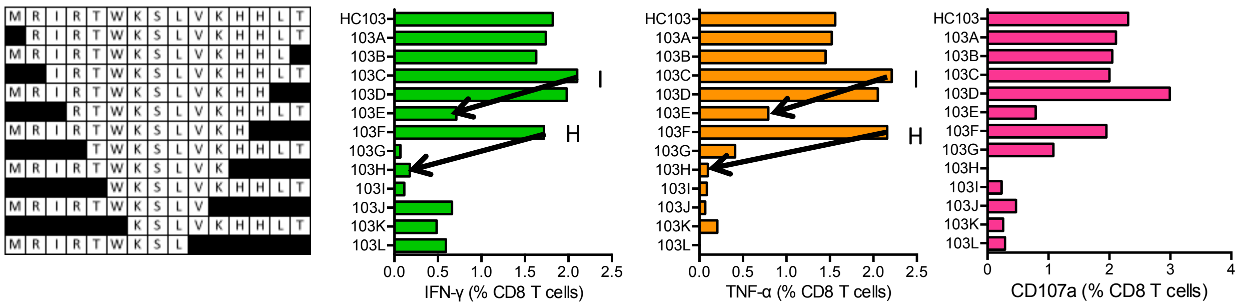

C **VID 418 - HLA-B*27:05 restriction of HC102 STCL for IH10 on HLA-transfected 721.221 cells**

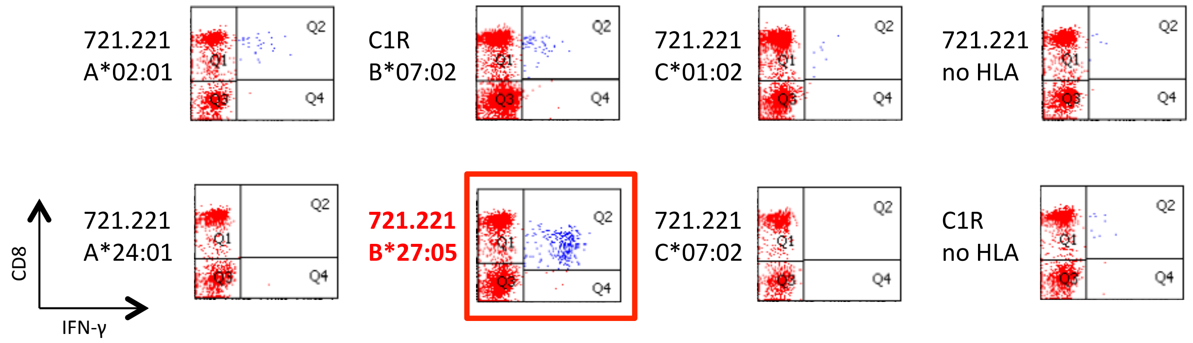

S14 Fig. HC103 MRIRTWKSLVKHH-LT (Vif) - Definition of CD8⁺ T-cell determinants. (A) The box. Peptide HC103 was recognized by volunteer 418 of the indicated HLA type and the optimal peptide and its HLA restriction are shown. '-' indicates junction between two adjacent HIVconsv regions. (B) Cryopreserved lymphocytes from the vaccine recipient were expanded by stimulation with 'parental' responder peptide for 10 days to establish STCL, which was subjected to ICS using serially truncated. IFN-γ (green) and TNF-α (orange) production and surface expression of CD107a (pink) served as the read-out. Arrows next to an amino acid indicate the peptide-terminal amino acid residue required for efficient peptide recognition. (C) 721.221 and C1R cells expressing all HLA alleles of volunteer 418 were used to determine the HLA restriction of the HC103-derived peptide by restimulation followed by flow cytometry.
