# Supplementary material for: Novel, in-natural-infection subdominant HIV-1 CD8+ T-cell epitopes revealed in human recipients of conserved-region T-cell vaccines
Source: PLoS One. 2017 Apr 27;12(4):e0176418. doi: 10.1371/journal.pone.0176418 (PMC5407754; doi:10.1371/journal.pone.0176418)
Supplement: S15 Fig — (A) The box. 15-mer peptide HC135 was recognized by volunteers 416 and 418 of the indicated HLA types. Optimal peptides and determined HLA restriction are shown. (B) Volunteers’ lymphocytes were expanded by stimulation with ‘parental’ peptide for 10 days to establish STCLs, which were subjected to ICS using serially truncated peptides monitoring IFN-γ (green) and TNF-α (orange) production and surface expression of CD107a (pink). Arrows next to an amino acid indicate the peptide-terminal amino acid residue required for efficient peptide recognition. (C) The same SCTLs from volunteers 416 (left) and 418 (right) were tested for recognition of overlapping 9-mer peptides. 721.221 and C1R cells expressing HLA alleles of volunteers 416 (D) and 417 (E) were used to determine the HLA restriction of peptide KLVSQGIRKV. (PDF) [file pone.0176418.s015.pdf]

A

**HC135 KLVSQGIRKVLFLDG (Pol)**

VID 416 - A*02:01 (A02) A*02:01 (A02) B*08:01 (B08) B*44:02 (B44) C*05:01 C*07:01

VID 418 - A*02:01 (A02) A*24:02 (A24) B*07:02 (B07) B*27:05 (B27) C*01:02 C*07:02

| | |
|---|---|
| **KLVSQGIRKV/HLA-A*02:01** | Predicted A*02:01, not reported, confirmed A*02:01, **'A-list' candidate** |
| **KLVSQGIRKVLF** | Not predicted, not reported |

B

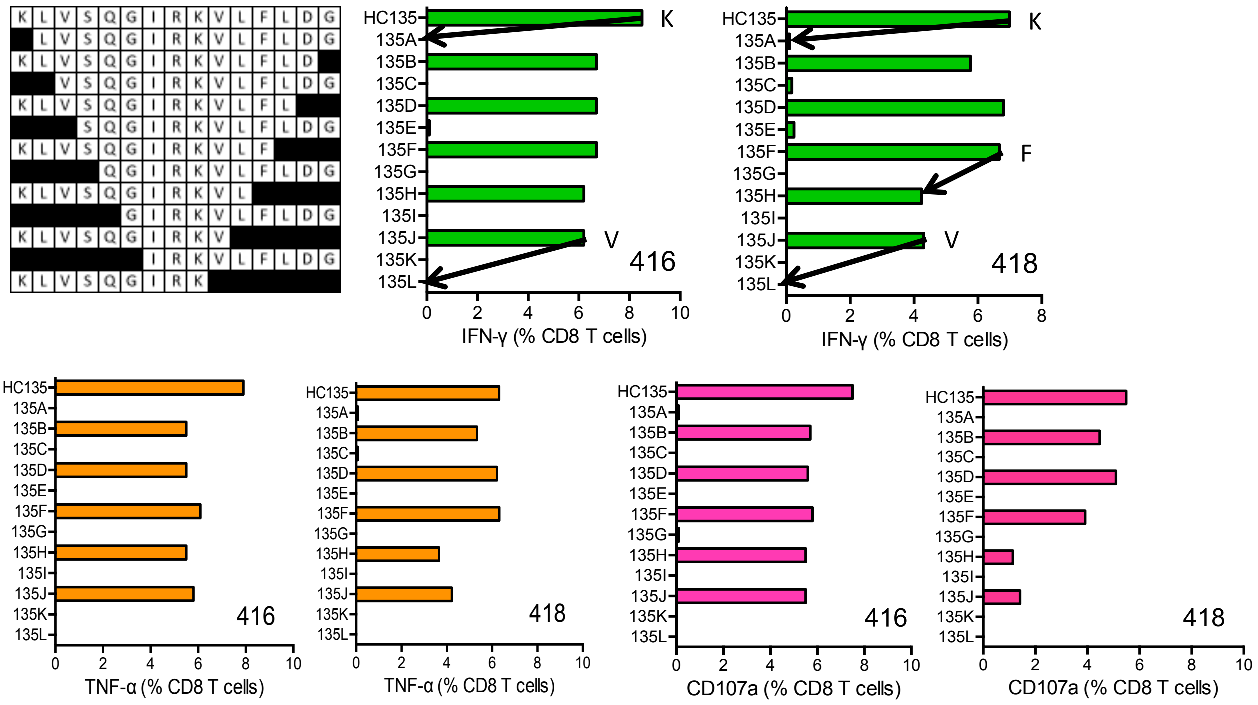


C

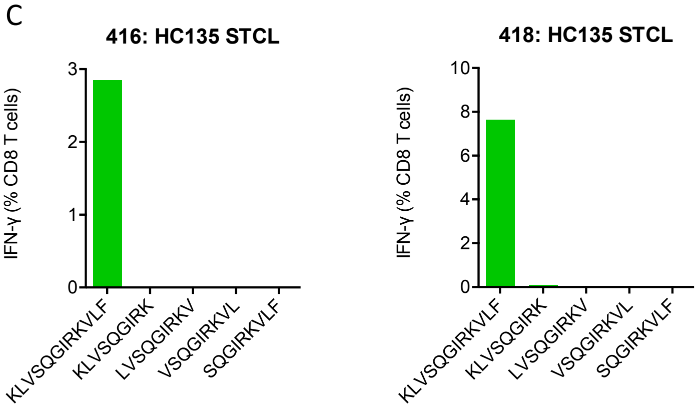

D VID 416 - HLA-A*02:01 restriction for KV10 by HC135 STCL on HLA-transfected 721.221 cells

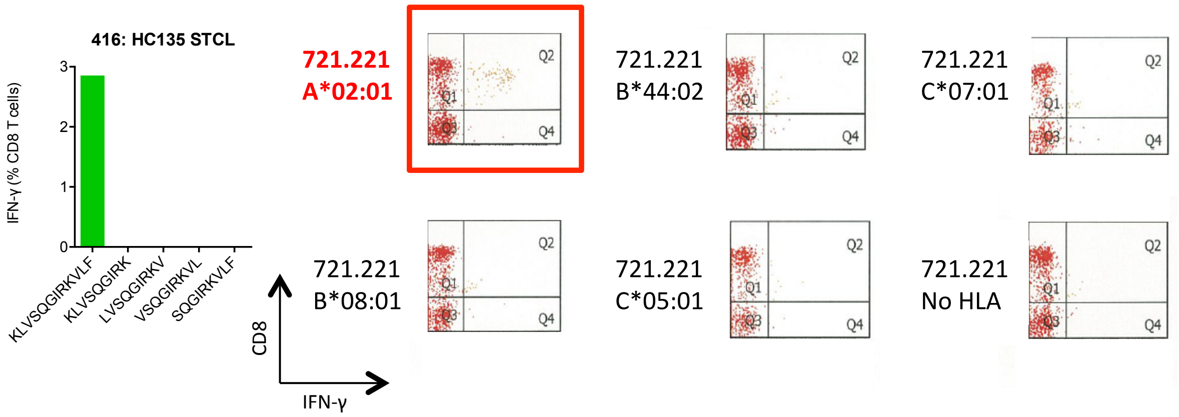


E VID 418 - HLA-A*02:01 restriction for KV10 by 418 HC135 STCL on HLA-transfected 721.221 cells

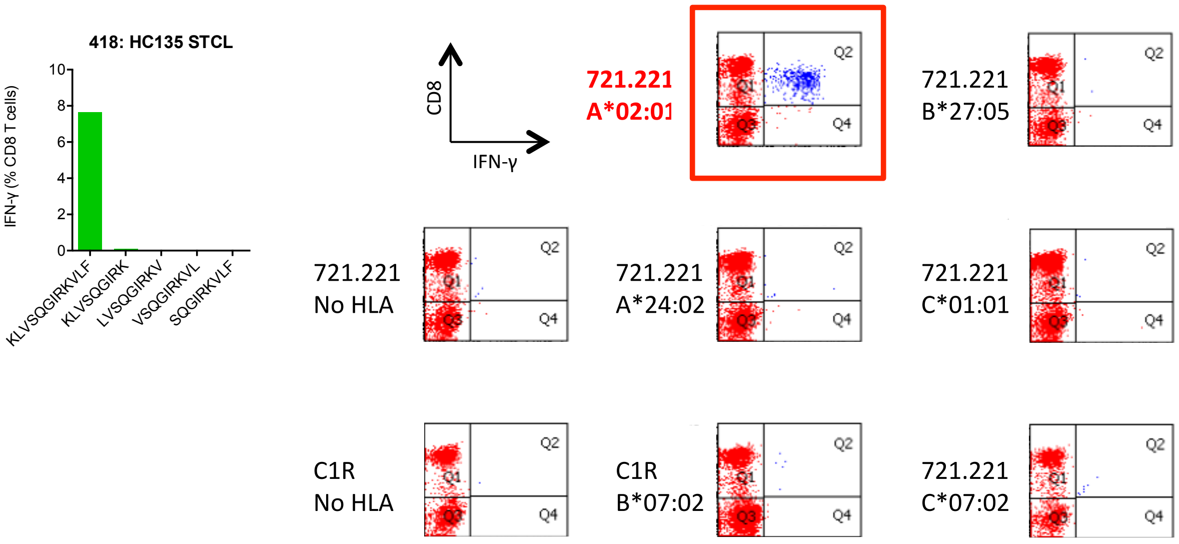


**S15 Fig. HC135 KLVSQGIRKVLFLDG (Pol) - Definition of CD8⁺ T-cell determinants.** (A) The box. 15-mer peptide HC135 was recognized by volunteers 416 and 418 of the indicated HLA types. Optimal peptides and determined HLA restriction are shown. (B) Volunteers' lymphocytes were expanded by stimulation with 'parental' peptide for 10 days to establish STCLs, which were subjected to ICS using serially truncated peptides monitoring IFN-γ (green) and TNF-α (orange) production and surface expression of CD107a (pink). Arrows next to an amino acid indicate the peptide-terminal amino acid residue required for efficient peptide recognition. (C) The same SCTLs from volunteers 416 (left) and 418 (right) were tested for recognition of overlapping 9-mer peptides. 721.221 and C1R cells expressing HLA alleles of volunteers 416 (D) and 417 (E) were used to determine the HLA restriction of peptide KLVSQGIRKV.
