# Supplementary material for: Novel, in-natural-infection subdominant HIV-1 CD8+ T-cell epitopes revealed in human recipients of conserved-region T-cell vaccines
Source: PLoS One. 2017 Apr 27;12(4):e0176418. doi: 10.1371/journal.pone.0176418 (PMC5407754; doi:10.1371/journal.pone.0176418)
Supplement: S16 Fig — (A) The box. Peptide HC139 was recognized by volunteer 404 of the indicated HLA type and the optimal peptides are shown. '-' indicates junction between two adjacent HIVconsv regions. (B) Cryopreserved lymphocytes from the vaccine recipient were expanded by stimulation with 'parental' peptide for 10 days to establish STCL, which was subjected to ICS using serially truncated peptides. IFN-γ (green) and TNF-α (orange) production and surface expression of CD107a (pink) served as the read-out. Arrows next to an amino acid indicate the peptide-terminal amino acid residue required for efficient peptide recognition. (C) The same SCTLs as in (B) were tested for recognition of overlapping 9-mer peptides. (PDF) [file pone.0176418.s016.pdf]

A

HC139 DKAQ-AKEIVASCDKC (Pol)

VID 404 - A*68:01 (A03) A*68:01 (A03) B*44:02 (B44) B*51:01 (B07) C*07:04 C*14:02

AKEIVASCDKC Not predicted, not reported

AKEIVASCD Not predicted, not reported

B

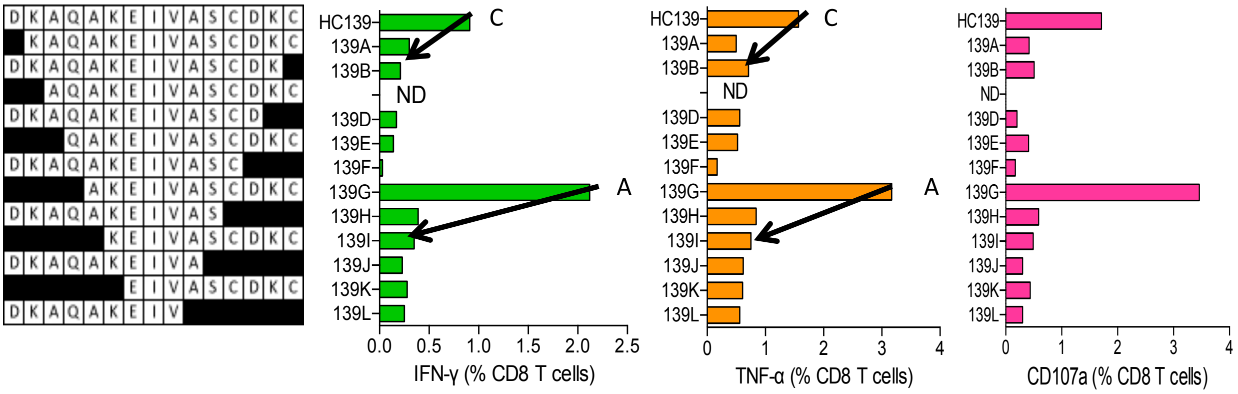

C

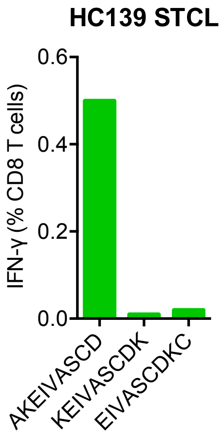

S16 Fig. HC139 DKAQ-AKEIVASCDKC (Pol)- Definition of CD8⁺ T-cell determinants. (A) The box. Peptide HC139 was recognized by volunteer 404 of the indicated HLA type and the optimal peptides are shown. '-' indicates junction between two adjacent HIVconsv regions. (B) Cryopreserved lymphocytes from the vaccine recipient were expanded by stimulation with 'parental' peptide for 10 days to establish STCL, which was subjected to ICS using serially truncated peptides. IFN-γ (green) and TNF-α (orange) production and surface expression of CD107a (pink) served as the read-out. Arrows next to an amino acid indicate the peptide-terminal amino acid residue required for efficient peptide recognition. (C) The same SCTLs as in (B) were tested for recognition of overlapping 9-mer peptides.
