# Supplementary material for: Novel, in-natural-infection subdominant HIV-1 CD8+ T-cell epitopes revealed in human recipients of conserved-region T-cell vaccines
Source: PLoS One. 2017 Apr 27;12(4):e0176418. doi: 10.1371/journal.pone.0176418 (PMC5407754; doi:10.1371/journal.pone.0176418)
Supplement: S17 Fig — (A) The box. Peptide HC145 was recognized by volunteer 404 of the indicated HLA type, and the optimal peptide is shown. (B) Cryopreserved lymphocytes from the vaccine recipient were expanded by stimulation with the 'parental' peptide for 10 days to establish STCL, which was subjected to ICS using serially truncated peptides. IFN-γ (green) and TNF-α (orange) production and surface expression of CD107a (pink) served as the read-out. Arrows next to an amino acid indicate the peptide-terminal amino acid residue required for efficient peptide recognition. (C) The same SCTLs were tested for recognition of overlapping 9-mer peptides. (PDF) [file pone.0176418.s017.pdf]

A

HC145 GQVDCSPGIWQLDCTH (Pol)

VID 404 - A*68:01 (A03) A*68:01 (A03) B*44:02 (B44) B*51:01 (B07) C*07:04 C*14:02

GIWQLDCTH   Not predicted, not reported

B

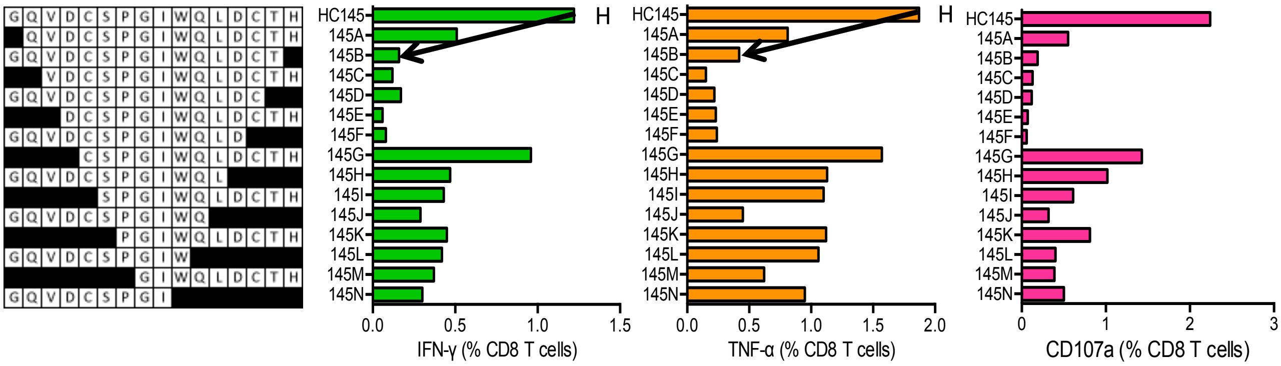

C

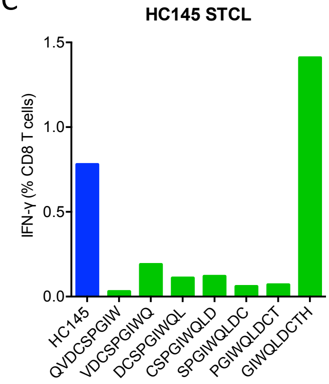

S17 Fig. HC145 GQVDCSPGIWQLDCTH (Pol) - Definition of CD8^{+} T-cell determinants. (A) The box. Peptide HC145 was recognized by volunteer 404 of the indicated HLA type, and the optimal peptide is shown. (B) Cryopreserved lymphocytes from the vaccine recipient were expanded by stimulation with the 'parental' peptide for 10 days to establish STCL, which was subjected to ICS using serially truncated peptides. IFN-γ (green) and TNF-α (orange) production and surface expression of CD107a (pink) served as the read-out. Arrows next to an amino acid indicate the peptide-terminal amino acid residue required for efficient peptide recognition. (C) The same SCTLs were tested for recognition of overlapping 9-mer peptides.
