# Supplementary material for: Novel, in-natural-infection subdominant HIV-1 CD8+ T-cell epitopes revealed in human recipients of conserved-region T-cell vaccines
Source: PLoS One. 2017 Apr 27;12(4):e0176418. doi: 10.1371/journal.pone.0176418 (PMC5407754; doi:10.1371/journal.pone.0176418)
Supplement: S18 Fig — (A) The box. Peptide HC164 was recognized by volunteers 404 and 410 of the indicated HLA types, and the optimal peptide is shown. (B) Cryopreserved lymphocytes from vaccine recipients 404 (left) and 410 (right) were expanded by stimulation with the 'parental' peptide for 10 days to establish STCLs, which were tested for recognition of overlapping 9-mer peptides. (PDF) [file pone.0176418.s018.pdf]

A

HC164 VQMAVFIHNFKRKGGI (Pol)
VID 404 - A*68:01 (A03) A*68:01 (A03) B*44:02 (B44) B*51:01 (B07) C*07:04 C*14:02
VID 410 - A*30:02 (A01) A*30:02 (A01) B*18:01 (B27) B*57:03 (B58) C*07:01 C*18:01
IHNFKRKGG Not predicted, not reported

B

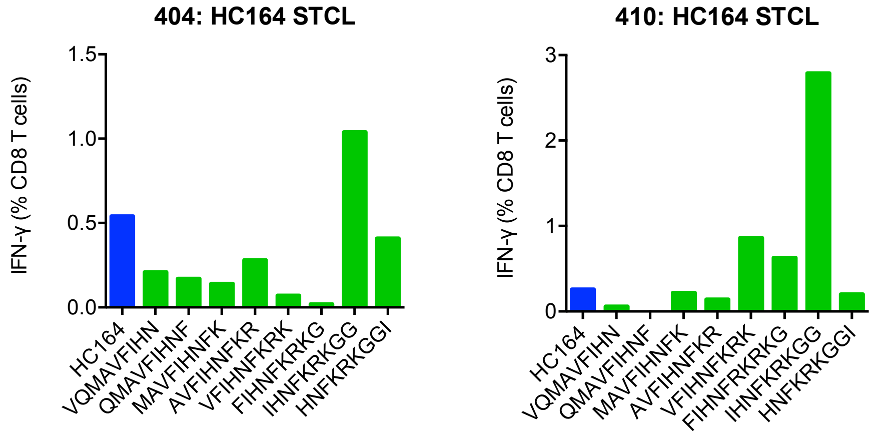

S18 Fig. HC164 VQMAVFIHNFKRKGGI (Pol) - Definition of CD8⁺ T-cell determinants. (A) The box. Peptide HC164 was recognized by volunteers 404 and 410 of the indicated HLA types, and the optimal peptide is shown. (B) Cryopreserved lymphocytes from vaccine recipients 404 (left) and 410 (right) were expanded by stimulation with the 'parental' peptide for 10 days to establish STCLs, which were tested for recognition of overlapping 9-mer peptides.
