# Supplementary material for: Novel, in-natural-infection subdominant HIV-1 CD8+ T-cell epitopes revealed in human recipients of conserved-region T-cell vaccines
Source: PLoS One. 2017 Apr 27;12(4):e0176418. doi: 10.1371/journal.pone.0176418 (PMC5407754; doi:10.1371/journal.pone.0176418)
Supplement: S19 Fig — (A) The box. Peptide HC176 was recognized by volunteer 413 of the indicated HLA type. Optimal peptides and their HLA restriction are shown. (B) Cryopreserved lymphocytes from the vaccine recipient were expanded by stimulation with 'parental' peptide for 10 days to establish STCL, which was subjected to ICS using serially truncated peptides. IFN-γ (green) and TNF-α (orange) production and surface expression of CD107a (pink) served as the read-out. Arrows next to an amino acid indicate the peptide-terminal amino acid residue required for efficient peptide recognition. (C) The same SCTLs as in (B) were tested for recognition of overlapping 9-mer peptides. (D) 721.221 cells expressing HLA-B*08:01 were used to confirm binding peptides. (PDF) [file pone.0176418.s019.pdf]

A

HC176 VVPRRKAKIIRDYGK (Pol)

VID 413 - A*01:01 (A01) A*03:01 (A03) B*08:01 (B08) B*44:02 (B44) C*05:01 C*07:01

VVPRRKAKII/HLA-B*08:01 Not predicted, not reported, confirmed B*08:01, **'A-list' candidate**

RKAKIIRDYGK/HLA-B*08:01 Not predicted, not reported, confirmed B*08:01, **'A-list' candidate**

KIIRDYGK/HLA-B*08:01 Predicted A*03:01, not reported, confirmed B*08:01, **'A-list' candidate**

B

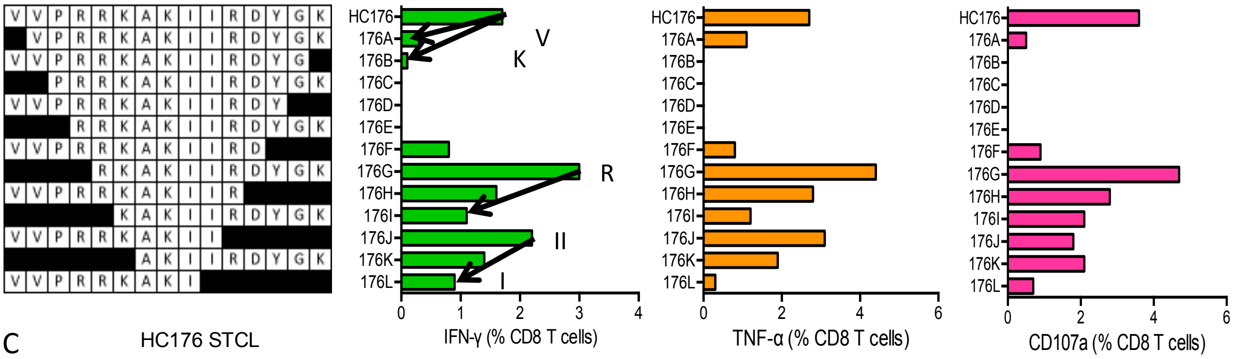

C

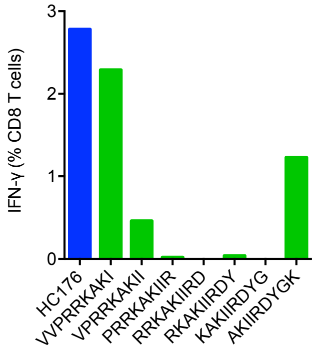

D **VID 413 - HLA-B*08:01 restriction for KK8 by HC176 STCL on HLA-B*08:01-transfected 721.221 cells**

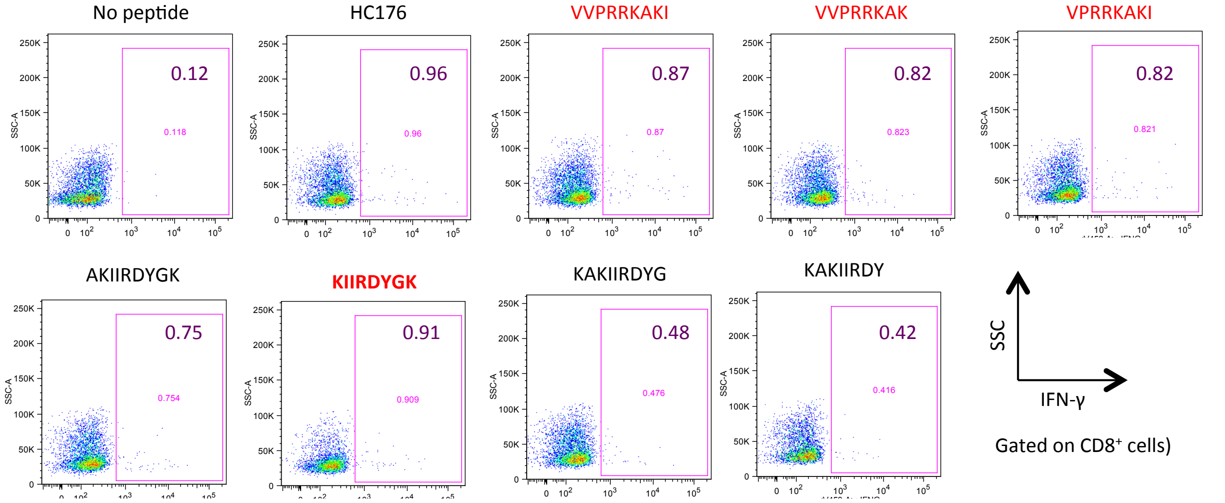

S19 Fig. HC176 VVPRRKAKIIRDYGK (Pol) - Definition of CD8$^+$ T-cell determinants. (A) The box. Peptide HC176 was recognized by volunteer 413 of the indicated HLA type. Optimal peptides and their HLA restriction are shown. (B) Cryopreserved lymphocytes from the vaccine recipient were expanded by stimulation with 'parental' peptide for 10 days to establish STCL, which was subjected to ICS using serially truncated peptides. IFN-γ (green) and TNF-α (orange) production and surface expression of CD107a (pink) served as the read-out. Arrows next to an amino acid indicate the peptide-terminal amino acid residue required for efficient peptide recognition. (C) The same SCTLs as in (B) were tested for recognition of overlapping 9-mer peptides. (D) 721.221 cells expressing HLA-B*08:01 were used to confirm binding peptides.
