# Supplementary material for: Novel, in-natural-infection subdominant HIV-1 CD8+ T-cell epitopes revealed in human recipients of conserved-region T-cell vaccines
Source: PLoS One. 2017 Apr 27;12(4):e0176418. doi: 10.1371/journal.pone.0176418 (PMC5407754; doi:10.1371/journal.pone.0176418)
Supplement: S1 Table — (PDF) [file pone.0176418.s020.pdf]

S1 Table. List of studied vaccine recipients and their HLA class I alleles and supertypes.

VID	HLA-A[a]		HLA-B[a]		HLA-C[a]	
403	A*01:01 (A01)	A*30:01 (A01/03)	B*13:02 (UC)	B*39:01 (B27)	C*06:02	C*07:01
404	A*68:01 (A03)	A*68:01 (A03)	B*44:02 (B44)	B*51:01 (B07)	C*07:04	C*14:02
406	A*03:01 (A03)	A*31:01 (A03)	B*40:01 (B44)	B*44:03 (B44)	C*03:04	C*04:01
409	A*01:01 (A01)	A*03:01 (A03)	B*07:02 (B07)	B*08:01 (B08)	C*07:01	C*07:02
410	A*30:02 (A01)	A*30:02 (A01)	B*18:01 (B27)	B*57:03 (B58)	C*07:01	C*18:01
411	A*02:01 (A02)	A*02:01 (A02)	B*08:01 (B08)	B*51:01 (B07)	C*03:03	C*07:01
413	A*01:01 (A01)	A*03:01 (A03)	B*08:01 (B08)	B*44:02 (B44)	C*05:01	C*07:01
415	A*02:01 (A02)	A*03:01 (A03)	B*07:02 (B07)	B*44:02 (B44)	C*07:02	C*07:02
416	A*02:01 (A02)	A*02:01 (A02)	B*08:01 (B08)	B*44:02 (B44)	C*05:01	C*07:01
417	A*03:01 (A03)	A*30:04 (A01)	B*35:01 (B07)	B*50:01 (B44)	C*04:01	C*06:02
418	A*02:01 (A02)	A*24:02 (A24)	B*07:02 (B07)	B*27:05 (B27)	C*01:02	C*07:02
421	A*02:01 (A02)	A*11:01 (A03)	B*35:03 (B07)	B*40:02 (B44)	C*02:02	C*12:03

a – HLA types are given as the allele names, and for HLA-A and -B alleles followed by its HLA supertype in brackets. UC – unclassified.
