# Supplementary material for: Novel, in-natural-infection subdominant HIV-1 CD8+ T-cell epitopes revealed in human recipients of conserved-region T-cell vaccines
Source: PLoS One. 2017 Apr 27;12(4):e0176418. doi: 10.1371/journal.pone.0176418 (PMC5407754; doi:10.1371/journal.pone.0176418)
Supplement: S2 Table — (PDF) [file pone.0176418.s021.pdf]

S2 Table. Parental 15-mer peptides and responders used in the mapping studies

Peptide	Sequence	Protein/Regions	HXB2 aa Positions	Responders
HC031	CTERQANFLGKIWPS	Gag/p2p7p1p6	426-440/63-77	421
HC049	**K**NFPISPIETVPVKLK	Pol/Protease, RT	153-168/97-99, 1-13	406
HC050	SPIETVPVKLKPGMD	Pol/RT	158-172/3-17	406
HC078	YFSVPLDEGFRKYTA	Pol/RT	270-284/115-129	417, 421
HC079	PLDEGFRKYTAFTIP	Pol/RT	274-288/119-133	417
HC080	GFRKYTAFTIPSINN	Pol/RT	278-292/123-137	411
HC081	YTAFTIPSINNETPG	Pol/RT	282-296/127-141	415
HC088	GSPAIFQSSMTKILE	Pol/RT	310-324/155-169	409, 421
HC091	ILEPFRAQNPEIVIY	Pol/RT	322-336/167-181	410
HC092	FRAQNPEIVIYQYMD**KK**	Pol/RT	326-342/171-187	410
HC093	**K**NPEIVIYQYMDDLYV	Pol/RT	329-344/174-189	411, 415, 416, 418
HC102	**K**QVDRMRIRTWKSLVK	Vif	11-26	403
HC103	MRIRTWKSLVKHHLT	Vif	16-30	418
HC135	KLVSQGIRKVLFLDG	Pol/RNase, Int	705-719/110-120, 1-4	416, 418
HC139	DKAQAKEIVASCDKC	Pol/Integrase	744-758/29-43	404
HC145	GQVDCSPGIWQLDCTH	Pol/Integrase	767-782/52-67	404
HC164	VQMAVFIHNFKRKGGI	Pol/Integrase	891-906/176-191	404, 410
HC176	VVPRRKAKIIRDYGK	Pol/Integrase	974-988/259-273	413

For some peptides, terminal lysine(s), shown in bold, were added to improve solubility
RT- reverse transcriptase
