# Supplementary material for: Novel, in-natural-infection subdominant HIV-1 CD8+ T-cell epitopes revealed in human recipients of conserved-region T-cell vaccines
Source: PLoS One. 2017 Apr 27;12(4):e0176418. doi: 10.1371/journal.pone.0176418 (PMC5407754; doi:10.1371/journal.pone.0176418)
Supplement: S3 Table — (PDF) [file pone.0176418.s022.pdf]

S3 Table. Epitope prediction

Volunteer ID: 403
HLA-A*01:01, HLA-A*30:01, HLA-B*13:02, HLA-B*39:01

AA	Length (aa)	Peptide	HLA	1-log50k (aff)	Affinity (nM)	% Rank	Label	Estimated Accuracy
**Peptide Sequence: KQVDRMRIRTWKSLVK HC102**								
7	10	RIRTWKSLVK	HLA-A*30:01	0.891	3	0.03	Strong binder	0.853
5	8	RMRIRTWK	HLA-A*30:01	0.913	3	0.01	Strong binder	0.853
5	11	RMRIRTWKSLV	HLA-A*30:01	0.845	5	0.1	Strong binder	0.853
7	9	RIRTWKSLV	HLA-A*30:01	0.827	7	0.12	Strong binder	0.853
9	8	RTWKSLVK	HLA-A*30:01	0.794	9	0.25	Strong binder	0.853
5	10	RMRIRTWKSL	HLA-A*30:01	0.786	10	0.25	Strong binder	0.853
7	8	RIRTWKSL	HLA-A*30:01	0.753	14	0.4	Strong binder	0.853
5	9	RMRIRTWKS	HLA-A*30:01	0.718	21	0.8	Weak binder	0.853
1	9	KQVDRMRIR	HLA-A*30:01	0.473	300	5.0	Combined binder	0.853
3	10	VDRMRIRTWK	HLA-A*30:01	0.42	530	6.0	Non-binder	0.853
2	11	QVDRMRIRTWK	HLA-A*30:01	0.419	538	6.0	Non-binder	0.853
1	8	KQVDRMRI	HLA-A*30:01	0.37	914	8.0	Non-binder	0.853
1	8	KQVDRMRI	HLA-B*13:02	0.098	NA	3.0	Non-binder	0.600
6	9	MRIRTWKSL	HLA-B*39:01	0.675	34	0.4	Strong binder	0.853
4	11	DRMRIRTWKSL	HLA-B*39:01	0.553	126	1.5	Weak binder	0.853
6	10	MRIRTWKSLV	HLA-B*39:01	0.458	354	3.0	Combined binder	0.853
**Peptide Sequence: MRIRTWKSLVKHHLT HC103**								
2	10	RIRTWKSLVK	HLA-A*30:01	0.891	3	0.03	Strong binder	0.853
2	9	RIRTWKSLV	HLA-A*30:01	0.827	7	0.12	Strong binder	0.853
4	8	RTWKSLVK	HLA-A*30:01	0.794	9	0.25	Strong binder	0.853
2	8	RIRTWKSL	HLA-A*30:01	0.753	14	0.4	Strong binder	0.853
2	11	RIRTWKSLVKH	HLA-A*30:01	0.665	38	1.0	Weak binder	0.853
4	11	RTWKSLVKHHL	HLA-A*30:01	0.623	59	1.5	Weak binder	0.853
7	8	KSLVKHHL	HLA-A*30:01	0.586	88	2.0	Weak binder	0.853
7	9	KSLVKHHLT	HLA-A*30:01	0.517	187	4.0	Combined binder	0.853
4	10	RTWKSLVKHH	HLA-A*30:01	0.421	524	6.0	Non-binder	0.853
4	9	RTWKSLVKH	HLA-A*30:01	0.399	664	7.0	Non-binder	0.853
5	10	TWKSLVKHHL	HLA-A*30:01	0.391	725	7.0	Non-binder	0.853
1	9	MRIRTWKSL	HLA-B*39:01	0.675	34	0.4	Strong binder	0.853
6	9	WKSLVKHHL	HLA-B*39:01	0.53	161	1.5	Weak binder	0.853
1	10	MRIRTWKSLV	HLA-B*39:01	0.458	354	3.0	Combined binder	0.853
**Peptide Sequence: IIRDYGKQMAGADCV HC178**								
1	10	IIRDYGKQMA	HLA-A*30:01	0.428	488	6.0	Combined binder	0.853
1	9	IIRDYGKQM	HLA-A*30:01	0.372	895	8.0	Non-binder	0.853
7	9	KQMAGADCV	HLA-B*13:02	0.163	NA	0.8	Weak binder	0.600
2	8	IRDYGKQM	HLA-B*39:01	0.378	833	4.0	Non-binder	0.853

Volunteer ID: 404
HLA-A*68:01, HLA-B*44:02, HLA-B*51:01, HLA-C*07:04, HLA-C14:02

Pos	Length (aa)	Peptide	HLA	1-log50k (aff)	Affinity (nM)	% Rank	Label	Estimated Accuracy
**Peptide Sequence: KLVSQGIRKVLFLDG HC135**								
2	8	LVSQGIRK	HLA-A*68:01	0.452	376	4.0	Combined binder	0.853

Pos	Length (aa)	Peptide	HLA	1-log50k (aff)	Affinity (nM)	% Rank	Label	Estimated Accuracy
4	10	SQGIRKVLFL	HLA-C*07:04	0.087	NA	4.0	Non-binder	0.503
**Peptide Sequence: DKAQAKEIVASCDKC HC139**								
7	8	EIVASCDK	HLA-A*68:01	0.566	109	2.0	Weak binder	0.853
4	11	QAKEIVASCDK	HLA-A*68:01	0.413	574	4.0	Non-binder	0.853
6	10	KEIVASCDKC	HLA-B*44:02	0.235	3943	4.0	Non-binder	0.853
6	9	KEIVASCDK	HLA-B*44:02	0.208	5282	4.0	Non-binder	0.853
**Peptide Sequence: GQVDCSPGIWQLDCTH HC145**								
1	10	GQVDCSPGIW	HLA-B*44:02	0.256	3126	3.0	Non-binder	0.853
5	8	CSPGIWQL	HLA-C*14:02	0.376	853	9.0	Non-binder	0.853
**Peptide Sequence: VQMAVFIHNFKRKGGI HC164**								
3	9	MAVFIHNFK	HLA-A*68:01	0.888	3	0.01	Strong binder	0.853
3	10	MAVFIHNFKR	HLA-A*68:01	0.861	5	0.03	Strong binder	0.853
4	9	AVFIHNFKR	HLA-A*68:01	0.746	16	0.3	Strong binder	0.853
4	8	AVFIHNFK	HLA-A*68:01	0.722	20	0.5	Strong binder	0.853
3	11	MAVFIHNFKRK	HLA-A*68:01	0.722	20	0.5	Strong binder	0.853
2	10	QMAVFIHNFK	HLA-A*68:01	0.718	21	0.5	Strong binder	0.853
2	11	QMAVFIHNFKR	HLA-A*68:01	0.67	35	0.8	Weak binder	0.853
5	8	VFIHNFKR	HLA-A*68:01	0.503	217	3.0	Combined binder	0.853
4	10	AVFIHNFKRK	HLA-A*68:01	0.499	225	3.0	Combined binder	0.853
6	8	FIHNFKRK	HLA-A*68:01	0.474	297	3.0	Combined binder	0.853
1	11	VQMAVFIHNFK	HLA-A*68:01	0.467	319	3.0	Combined binder	0.853
3	8	MAVFIHNF	HLA-A*68:01	0.372	890	5.0	Non-binder	0.853
1	10	VQMAVFIHNF	HLA-B*44:02	0.337	1306	1.5	Weak binder	0.853
3	8	MAVFIHNF	HLA-B*51:01	0.24	NA	2.0	Weak binder	0.853
1	10	VQMAVFIHNF	HLA-C*07:04	0.115	NA	2.0	Weak binder	0.503
1	10	VQMAVFIHNF	HLA-C*14:02	0.549	132	3.0	Combined binder	0.853
2	9	QMAVFIHNF	HLA-C*14:02	0.442	421	6.0	Combined binder	0.853
3	8	MAVFIHNF	HLA-C*14:02	0.398	673	7.0	Non-binder	0.853

Volunteer ID: 406

HLA-A*03:01, HLA-A*31:01, HLA-B*40:01, HLA-B*44:03, HLA-C*03:04, HLA-C*04:01

Pos	Length (aa)	Peptide	HLA	1-log50k (aff)	Affinity (nM)	% Rank	Label	Estimated Accuracy
**Peptide Sequence: KNFPISPIETVPVKLK HC049**								
4	11	PISPIETVPVK	HLA-A*03:01	0.252	3260	4.0	Non-binder	0.853
8	8	IETVPVKL	HLA-B*40:01	0.585	89	1.0	Weak binder	0.853
8	8	IETVPVKL	HLA-B*44:03	0.369	923	1.5	Weak binder	0.853
3	11	FPISPIETVPV	HLA-C*03:04	0.545	138	1.5	Weak binder	0.694
5	9	ISPIETVPV	HLA-C*03:04	0.532	158	2.0	Weak binder	0.694
5	11	ISPIETVPVKL	HLA-C*03:04	0.439	433	5.0	Combined binder	0.694
6	8	SPIETVPV	HLA-C*03:04	0.417	548	6.0	Non-binder	0.694
3	9	FPISPIETV	HLA-C*03:04	0.411	587	6.0	Non-binder	0.694
1	8	KNFPISPI	HLA-C*03:04	0.404	629	7.0	Non-binder	0.694
6	10	SPIETVPVKL	HLA-C*03:04	0.367	944	9.0	Non-binder	0.694

Pos	Length (aa)	Peptide	HLA	1-log50k (aff)	Affinity (nM)	% Rank	Label	Estimated Accuracy
5	9	ISPIETVPV	HLA-C*04:01	0.138	NA	1.5	Weak binder	0.762
5	11	ISPIETVPVKL	HLA-C*04:01	0.123	NA	2.0	Weak binder	0.762
2	10	NFPISPIETV	HLA-C*04:01	0.085	NA	4.0	Non-binder	0.762
**Peptide Sequence: SPIETVPVKLKPGMD HC050**								
3	8	IETVPVKL	HLA-B*40:01	0.585	89	1.0	Weak binder	0.853
3	8	IETVPVKL	HLA-B*44:03	0.369	923	1.5	Weak binder	0.853
1	8	SPIETVPV	HLA-C*03:04	0.417	548	6.0	Non-binder	0.694
1	10	SPIETVPVKL	HLA-C*03:04	0.367	944	9.0	Non-binder	0.694
**Peptide Sequence: MRIRTWKSLVKHHLT HC103**								
4	8	RTWKSLVK	HLA-A*03:01	0.702	25	0.08	Strong binder	0.853
2	10	RIRTWKSLVK	HLA-A*03:01	0.642	48	0.17	Strong binder	0.853
4	9	RTWKSLVKH	HLA-A*03:01	0.39	738	2.0	Weak binder	0.853
2	11	RIRTWKSLVKH	HLA-A*03:01	0.339	1279	3.0	Non-binder	0.853
4	10	RTWKSLVKHH	HLA-A*03:01	0.322	1527	3.0	Non-binder	0.853
4	8	RTWKSLVK	HLA-A*31:01	0.641	48	0.8	Weak binder	0.853
2	10	RIRTWKSLVK	HLA-A*31:01	0.568	108	1.5	Weak binder	0.853
4	9	RTWKSLVKH	HLA-A*31:01	0.382	803	4.0	Non-binder	0.853
4	11	RTWKSLVKHHL	HLA-A*31:01	0.373	887	4.0	Non-binder	0.853
4	11	RTWKSLVKHHL	HLA-C*03:04	0.473	300	4.0	Combined binder	0.694
7	8	KSLVKHHL	HLA-C*03:04	0.397	680	7.0	Non-binder	0.694

Volunteer ID: 409

HLA-A*01:01, HLA-A*03:01, HLA-B*07:02, HLA-B*08:01, HLA-C*07:01, HLA-C*07:02

Pos	Length (aa)	Peptide	HLA	1-log50k (aff)	Affinity (nM)	% Rank	Label	Estimated Accuracy
**Peptide Sequence: GSPAIFQSSMTKILE HC088**								
4	9	AIFQSSMTK	HLA-A*03:01	0.75	15	0.03	Strong binder	0.853
5	8	IFQSSMTK	HLA-A*03:01	0.381	809	2.0	Weak binder	0.853
2	9	SPAIFQSSM	HLA-B*07:02	0.799	9	0.08	Strong binder	0.853
2	10	SPAIFQSSMT	HLA-B*07:02	0.394	701	2.0	Weak binder	0.853
2	8	SPAIFQSS	HLA-B*07:02	0.33	1402	3.0	Non-binder	0.853
2	9	SPAIFQSSM	HLA-B*08:01	0.313	1698	4.0	Non-binder	0.853
**Peptide Sequence: MRIRTWKSLVKHHLT HC103**								
4	8	RTWKSLVK	HLA-A*03:01	0.702	25	0.08	Strong binder	0.853
2	10	RIRTWKSLVK	HLA-A*03:01	0.642	48	0.17	Strong binder	0.853
4	9	RTWKSLVKH	HLA-A*03:01	0.39	738	2.0	Weak binder	0.853
2	11	RIRTWKSLVKH	HLA-A*03:01	0.339	1279	3.0	Non-binder	0.853
4	10	RTWKSLVKHH	HLA-A*03:01	0.322	1527	3.0	Non-binder	0.853
2	8	RIRTWKSL	HLA-B*07:02	0.572	103	0.8	Weak binder	0.853
4	11	RTWKSLVKHHL	HLA-B*07:02	0.279	2450	4.0	Non-binder	0.853
1	9	MRIRTWKSL	HLA-B*07:02	0.276	2527	4.0	Non-binder	0.853
2	8	RIRTWKSL	HLA-B*08:01	0.52	181	0.8	Weak binder	0.853
1	9	MRIRTWKSL	HLA-B*08:01	0.433	459	1.5	Weak binder	0.853
2	9	RIRTWKSLV	HLA-B*08:01	0.356	1059	3.0	Non-binder	0.853
4	11	RTWKSLVKHHL	HLA-C*07:01	0.182	NA	1.0	Weak binder	0.556

Pos	Length (aa)	Peptide	HLA	1-log50k (aff)	Affinity (nM)	% Rank	Label	Estimated Accuracy
1	9	MRIRTWKSL	HLA-C*07:01	0.121	NA	3.0	Non-binder	0.556
4	11	RTWKSLVKHHL	HLA-C*07:02	0.171	NA	3.0	Non-binder	0.541
**Peptide Sequence: RKAKIIRDYGKQMAG HC177**								
4	8	KIIRDYGK	HLA-A*03:01	0.519	183	0.8	Weak binder	0.853
2	10	KAKIIRDYGK	HLA-A*03:01	0.309	1768	3.0	Non-binder	0.853
5	9	IIRDYGKQM	HLA-B*07:02	0.321	1551	3.0	Non-binder	0.853

Volunteer ID: 410

HLA-A*30:02, HLA-B*18:01, HLA-B*57:02, HLA-C*07:01, HLA-C*18:01

Pos	Length (aa)	Peptide	HLA	1-log50k (aff)	Affinity (nM)	% Rank	Label	Estimated Accuracy
**Peptide Sequence: ILEPFRAQNPEIVIY HC091**								
7	9	AQNPEIVIY	HLA-A*30:02	0.598	77	1.0	Weak binder	0.853
6	10	RAQNPEIVIY	HLA-A*30:02	0.562	114	1.5	Weak binder	0.853
6	9	RAQNPEIVI	HLA-B*57:02	0.23	NA	2.0	Weak binder	0.739
6	9	RAQNPEIVI	HLA-C*07:01	0.137	NA	3.0	Non-binder	0.556
5	10	FRAQNPEIVI	HLA-C*07:01	0.108	NA	4.0	Non-binder	0.556
5	10	FRAQNPEIVI	HLA-C*18:01	0.077	NA	4.0	Non-binder	0.733
5	9	FRAQNPEIV	HLA-C*18:01	0.077	NA	4.0	Non-binder	0.733
5	8	FRAQNPEI	HLA-C*18:01	0.076	NA	4.0	Non-binder	0.733
**Peptide Sequence: FRAQNPEIVIYQYMDKK HC092**								
3	11	AQNPEIVIYQY	HLA-A*30:02	0.665	37	0.8	Weak binder	0.853
3	9	AQNPEIVIY	HLA-A*30:02	0.598	77	1.0	Weak binder	0.853
2	10	RAQNPEIVIY	HLA-A*30:02	0.562	114	1.5	Weak binder	0.853
6	8	PEIVIYQY	HLA-B*18:01	0.546	136	0.8	Weak binder	0.853
5	9	NPEIVIYQY	HLA-B*18:01	0.515	189	0.8	Weak binder	0.853
6	9	PEIVIYQYM	HLA-B*18:01	0.35	1138	3.0	Non-binder	0.853
2	9	RAQNPEIVI	HLA-B*57:02	0.23	NA	2.0	Weak binder	0.739
2	9	RAQNPEIVI	HLA-C*07:01	0.137	NA	3.0	Non-binder	0.556
1	10	FRAQNPEIVI	HLA-C*07:01	0.108	NA	4.0	Non-binder	0.556
1	10	FRAQNPEIVI	HLA-C*18:01	0.077	NA	4.0	Non-binder	0.733
1	9	FRAQNPEIV	HLA-C*18:01	0.077	NA	4.0	Non-binder	0.733
1	8	FRAQNPEI	HLA-C*18:01	0.076	NA	4.0	Non-binder	0.733
**Peptide Sequence: KNPEIVIYQYMDDLYV HC093**								
5	11	IVIYQYMDDLY	HLA-A*30:02	0.693	28	0.5	Strong binder	0.853
6	10	VIYQYMDDLY	HLA-A*30:02	0.686	30	0.5	Strong binder	0.853
8	8	YQYMDDLY	HLA-A*30:02	0.679	32	0.8	Weak binder	0.853
7	9	IYQYMDDLY	HLA-A*30:02	0.539	147	1.5	Weak binder	0.853
1	10	KNPEIVIYQY	HLA-A*30:02	0.45	383	3.0	Combined binder	0.853
1	8	KNPEIVIY	HLA-A*30:02	0.376	858	4.0	Non-binder	0.853
3	8	PEIVIYQY	HLA-B*18:01	0.546	136	0.8	Weak binder	0.853
2	9	NPEIVIYQY	HLA-B*18:01	0.515	189	0.8	Weak binder	0.853
8	8	YQYMDDLY	HLA-B*18:01	0.368	933	2.0	Weak binder	0.853
3	9	PEIVIYQYM	HLA-B*18:01	0.35	1138	3.0	Non-binder	0.853

Pos	Length (aa)	Peptide	HLA	1-log50k (aff)	Affinity (nM)	% Rank	Label	Estimated Accuracy
8	9	YQYMDDLYV	HLA-C*07:01	0.135	NA	3.0	Non-binder	0.556
9	8	QYMDDLYV	HLA-C*18:01	0.148	NA	0.5	Strong binder	0.733
7	10	IYQYMDDLYV	HLA-C*18:01	0.136	NA	0.8	Weak binder	0.733
8	9	YQYMDDLYV	HLA-C*18:01	0.088	NA	3.0	Non-binder	0.733
7	8	IYQYMDDL	HLA-C*18:01	0.084	NA	3.0	Non-binder	0.733
1	11	KNPEIVIYQYM	HLA-C*18:01	0.081	NA	3.0	Non-binder	0.733
**Peptide Sequence: VQMAVFIHNFKRKGGI HC164**								
2	9	QMAVFIHNF	HLA-A*30:02	0.403	638	3.0	Non-binder	0.853
1	10	VQMAVFIHNF	HLA-A*30:02	0.402	644	3.0	Non-binder	0.853
1	11	VQMAVFIHNFK	HLA-A*30:02	0.351	1117	4.0	Non-binder	0.853
1	10	VQMAVFIHNF	HLA-B*18:01	0.39	738	2.0	Weak binder	0.853
3	8	MAVFIHNF	HLA-B*18:01	0.324	1509	3.0	Non-binder	0.853
3	8	MAVFIHNF	HLA-B*57:02	0.292	NA	0.8	Weak binder	0.739
2	9	QMAVFIHNF	HLA-B*57:02	0.156	NA	4.0	Non-binder	0.739
1	10	VQMAVFIHNF	HLA-C*07:01	0.179	NA	1.0	Weak binder	0.556
3	8	MAVFIHNF	HLA-C*07:01	0.114	NA	4.0	Non-binder	0.556
1	10	VQMAVFIHNF	HLA-C*18:01	0.079	NA	4.0	Non-binder	0.733

Volunteer ID: 411

HLA-A*02:01, HLA-B*08:01, HLA-B*51:01, HLA-C*03:03, HLA-C*07:01

Pos	Length (aa)	Peptide	HLA	1-log50k (aff)	Affinity (nM)	% Rank	Label	Estimated Accuracy
**Peptide Sequence: GFRKYTAFTIPSINN HC080**								
5	9	YTAFTIPSI	HLA-A*02:01	0.611	68	2.0	Weak binder	0.853
1	8	GFRKYTAF	HLA-B*08:01	0.35	1139	3.0	Non-binder	0.853
2	9	FRKYTAFTI	HLA-B*08:01	0.313	1689	4.0	Non-binder	0.853
6	8	TAFTIPSI	HLA-B*51:01	0.345	NA	0.8	Weak binder	0.853
5	9	YTAFTIPSI	HLA-B*51:01	0.206	NA	3.0	Non-binder	0.853
6	8	TAFTIPSI	HLA-C*03:03	0.433	459	5.0	Combined binder	0.694
5	9	YTAFTIPSI	HLA-C*03:03	0.522	176	2.0	Weak binder	0.694
3	8	RKYTAFTI	HLA-C*07:01	0.17	NA	1.5	Weak binder	0.556
3	11	RKYTAFTIPSI	HLA-C*07:01	0.155	NA	1.5	Weak binder	0.556
4	10	KYTAFTIPSI	HLA-C*07:01	0.13	NA	3.0	Non-binder	0.556
**Peptide Sequence: GSPAIFQSSMTKILE HC088**								
6	8	FQSSMTKI	HLA-A*02:01	0.443	412	4.0	Combined binder	0.853
4	10	AIFQSSMTKI	HLA-A*02:01	0.398	671	5.0	Non-binder	0.853
2	9	SPAIFQSSM	HLA-B*08:01	0.313	1698	4.0	Non-binder	0.853
2	9	SPAIFQSSM	HLA-B*51:01	0.268	NA	1.5	Weak binder	0.853
6	8	FQSSMTKI	HLA-B*51:01	0.198	NA	3.0	Non-binder	0.853
4	10	AIFQSSMTKI	HLA-C*03:03	0.472	304	4.0	Combined binder	0.694
4	11	AIFQSSMTKIL	HLA-C*03:03	0.439	432	5.0	Combined binder	0.694

Pos	Length (aa)	Peptide	HLA	1-log50k (aff)	Affinity (nM)	% Rank	Label	Estimated Accuracy
6	9	FQSSMTKIL	HLA-C*03:03	0.403	638	7.0	Non-binder	0.694
6	8	FQSSMTKI	HLA-C*03:03	0.392	720	7.0	Non-binder	0.694
**Peptide Sequence: KNPEIVIYQYMDDLYV HC093**								
8	9	YQYMDDLYV	HLA-A*02:01	0.788	10	0.4	Strong binder	0.853
6	11	VIYQYMDDLYV	HLA-A*02:01	0.682	31	1.0	Weak binder	0.853
2	10	NPEIVIYQYM	HLA-B*51:01	0.242	NA	2.0	Weak binder	0.853
2	9	NPEIVIYQY	HLA-B*51:01	0.164	NA	4.0	Non-binder	0.853
8	9	YQYMDDLYV	HLA-C*03:03	0.484	266	4.0	Combined binder	0.694
6	11	VIYQYMDDLYV	HLA-C*03:03	0.395	697	7.0	Non-binder	0.694
8	9	YQYMDDLYV	HLA-C*07:01	0.135	NA	3.0	Non-binder	0.556
**Peptide Sequence: PEWEFVNTPPLVKLW HC117**								
5	8	FVNTPPLV	HLA-A*02:01	0.663	38	1.5	Weak binder	0.853
5	10	FVNTPPLVKL	HLA-A*02:01	0.408	604	5.0	Non-binder	0.853
8	8	TPPLVKLW	HLA-B*51:01	0.238	NA	2.0	Weak binder	0.853
5	11	FVNTPPLVKLW	HLA-B*51:01	0.167	NA	4.0	Non-binder	0.853
5	10	FVNTPPLVKL	HLA-C*03:03	0.568	108	1.5	Weak binder	0.694
5	8	FVNTPPLV	HLA-C*03:03	0.565	111	1.5	Weak binder	0.694
3	9	WEFVNTPPL	HLA-C*07:01	0.272	NA	0.12	Strong binder	0.556
3	10	WEFVNTPPLV	HLA-C*07:01	0.122	NA	3.0	Non-binder	0.556
**Peptide Sequence: LLRAIEAQQHLLQLT HC190**								
1	11	LLRAIEAQQHL	HLA-B*08:01	0.471	307	1.0	Weak binder	0.853
6	9	EAQQHLLQL	HLA-B*08:01	0.372	892	3.0	Non-binder	0.853
3	9	RAIEAQQHL	HLA-C*03:03	0.611	67	0.8	Weak binder	0.694
3	10	RAIEAQQHLL	HLA-C*03:03	0.589	85	0.8	Weak binder	0.694
7	8	AQQHLLQL	HLA-C*03:03	0.383	790	8.0	Non-binder	0.694
3	10	RAIEAQQHLL	HLA-C*07:01	0.173	NA	1.0	Weak binder	0.556
3	9	RAIEAQQHL	HLA-C*07:01	0.147	NA	2.0	Weak binder	0.556
7	8	AQQHLLQL	HLA-C*07:01	0.112	NA	4.0	Non-binder	0.556

Volunteer ID: 413

HLA-A*01:01, HLA-A*03:01, HLA-B*08:01, HLA-B*44:02, HLA-C*05:01, HLA-C*07:01

Pos	Length (aa)	Peptide	HLA	1-log50k (aff)	Affinity (nM)	% Rank	Label	Estimated Accuracy
**Peptide Sequence: VQMAVFIHNFKRKGGI HC164**								
4	8	AVFIHNFK	HLA-A*03:01	0.713	22	0.05	Strong binder	0.853
2	10	QMAVFIHNFK	HLA-A*03:01	0.623	59	0.25	Strong binder	0.853
4	10	AVFIHNFKRK	HLA-A*03:01	0.601	75	0.3	Strong binder	0.853
1	11	VQMAVFIHNFK	HLA-A*03:01	0.598	78	0.3	Strong binder	0.853
3	9	MAVFIHNFK	HLA-A*03:01	0.515	191	0.8	Weak binder	0.853
4								
6	8	FIHNFKRK	HLA-A*03:01	0.453	372	1.5	Weak binder	0.853
3	11	MAVFIHNFKRK	HLA-A*03:01	0.37	908	2.0	Weak binder	0.853
2	11	QMAVFIHNFKR	HLA-A*03:01	0.293	2100	4.0	Non-binder	0.853
3	10	MAVFIHNFKR	HLA-A*03:01	0.252	3282	4.0	Non-binder	0.853
9	8	NFKRKGGI	HLA-B*08:01	0.476	289	1.0	Weak binder	0.853

Pos	Length (aa)	Peptide	HLA	1-log50k (aff)	Affinity (nM)	% Rank	Label	Estimated Accuracy
6	11	FIHNFKRKGGI	HLA-B*08:01	0.304	1867	4.0	Non-binder	0.853
1	10	VQMAVFIHNF	HLA-B*44:02	0.337	1306	1.5	Weak binder	0.853
1	10	VQMAVFIHNF	HLA-C*07:01	0.179	NA	1.0	Weak binder	0.556
3	8	MAVFIHNF	HLA-C*07:01	0.114	NA	4.0	Non-binder	0.556
**Peptide Sequence: VVPRRKAKIIRDYGK HC176**								
8	8	KIIRDYGK	HLA-A*03:01	0.519	183	0.8	Weak binder	0.853
6	10	KAKIIRDYGK	HLA-A*03:01	0.309	1768	3.0	Non-binder	0.853
2	9	VPRRKAKII	HLA-B*08:01	0.537	149	0.5	Strong binder	0.853
2	8	VPRRKAKI	HLA-B*08:01	0.482	273	0.8	Weak binder	0.853
**Peptide Sequence: IIRDYGKQMAGADCV HC178**								
1	10	IIRDYGKQMA	HLA-A*30:01	0.428	488	6.0	Combined binder	0.853
1	9	IIRDYGKQM	HLA-A*30:01	0.372	895	8.0	Non-binder	0.853
7	9	KQMAGADCV	HLA-B*13:02	0.163	NA	0.8	Weak binder	0.600
2	8	IRDYGKQM	HLA-B*39:01	0.378	833	4.0	Non-binder	0.853

Volunteer ID: 414

HLA-A*24:02, HLA-A*33:01, HLA-B*14:01, HLA-B*44:02, HLA-C*05:01, HLA-C*08:02

Pos	Length (aa)	Peptide	HLA	1-log50k (aff)	Affinity (nM)	% Rank	Label	Estimated Accuracy
**Peptide Sequence: KQYWQATWIPEWEFVN HC115**								
3	10	YWQATWIPEW	HLA-A*24:02	0.812	8	0.08	Strong binder	0.853
7	8	TWIPEWEF	HLA-A*24:02	0.77	12	0.1	Strong binder	0.853
2	11	QYWQATWIPEW	HLA-A*24:02	0.739	17	0.15	Strong binder	0.853
2	8	QYWQATWI	HLA-A*24:02	0.686	30	0.3	Strong binder	0.853
7	9	TWIPEWEFV	HLA-A*24:02	0.545	138	1.0	Weak binder	0.853
4	11	WQATWIPEWEF	HLA-A*24:02	0.515	190	1.0	Weak binder	0.853
2	9	QYWQATWIP	HLA-A*24:02	0.42	529	2.0	Weak binder	0.853
6	9	ATWIPEWEF	HLA-A*24:02	0.401	650	2.0	Weak binder	0.853
4	9	WQATWIPEW	HLA-A*24:02	0.401	650	2.0	Weak binder	0.853
1	8	KQYWQATW	HLA-A*24:02	0.362	997	3.0	Non-binder	0.853
3	8	YWQATWIP	HLA-A*24:02	0.358	1041	3.0	Non-binder	0.853
5	10	QATWIPEWEF	HLA-A*24:02	0.352	1104	3.0	Non-binder	0.853
4	9	WQATWIPEW	HLA-B*44:02	0.416	556	0.8	Weak binder	0.853
1	8	KQYWQATW	HLA-B*44:02	0.353	1103	1.5	Weak binder	0.853
4	11	WQATWIPEWEF	HLA-B*44:02	0.324	1498	2.0	Weak binder	0.853
1	9	KQYWQATWI	HLA-B*44:02	0.209	5203	4.0	Non-binder	0.853
**Peptide Sequence: LLRAIEAQQHLLQLT HC190**								
6	9	EAQQHLLQL	HLA-B*14:01	0.29	2171	4.0	Non-binder	0.853
5	8	IEAQQHLL	HLA-B*44:02	0.436	449	0.8	Weak binder	0.853
5	10	IEAQQHLLQL	HLA-B*44:02	0.429	481	0.8	Weak binder	0.853
5	11	IEAQQHLLQLT	HLA-B*44:02	0.323	1524	2.0	Weak binder	0.853
3	10	RAIEAQQHLL	HLA-C*05:01	0.364	975	4.0	Non-binder	0.853
3	10	RAIEAQQHLL	HLA-C*08:02	0.396	688	3.0	Non-binder	0.797

Volunteer ID: 415

HLA-A*02:01, HLA-A*03:01, HLA-B*07:02, HLA-B*44:02, HLA-C*07:02

Pos	Length (aa)	Peptide	HLA	1-log50k (aff)	Affinity (nM)	% Rank	Label	Estimated Accuracy
**Peptide Sequence: YTAFTIPSINNETPG HC081**								
1	9	YTAFTIPSI	HLA-A*02:01	0.611	68	2.0	Weak binder	0.853
6	8	IPSINNET	HLA-B*07:02	0.289	2192	4.0	Non-binder	0.853
1	9	YTAFTIPSI	HLA-C*07:02	0.14	NA	4.0	Non-binder	0.541
**Peptide Sequence: KNPEIVIYQYMDDLYV HC093**								
8	9	YQYMDDLYV	HLA-A*02:01	0.788	10	0.4	Strong binder	0.853
6	11	VIYQYMDDLYV	HLA-A*02:01	0.682	31	1.0	Weak binder	0.853
6	10	VIYQYMDDLY	HLA-A*03:01	0.36	1021	3.0	Non-binder	0.853
5	11	IVIYQYMDDLY	HLA-A*03:01	0.264	2887	4.0	Non-binder	0.853
2	10	NPEIVIYQYM	HLA-B*07:02	0.27	2690	4.0	Non-binder	0.853
3	8	PEIVIYQY	HLA-B*44:02	0.412	582	0.8	Weak binder	0.853
3	9	PEIVIYQYM	HLA-B*44:02	0.297	2004	2.0	Weak binder	0.853
8	8	YQYMDDLY	HLA-B*44:02	0.239	3759	3.0	Non-binder	0.853
8	9	YQYMDDLYV	HLA-C*07:02	0.197	NA	1.5	Weak binder	0.541
9	8	QYMDDLYV	HLA-C*07:02	0.169	NA	3.0	Non-binder	0.541
**Peptide Sequence: MIVWQVDRMRIRTWK HC101**								
1	11	MIVWQVDRMRI	HLA-A*02:01	0.371	902	6.0	Non-binder	0.853
8	8	RMRIRTWK	HLA-A*03:01	0.681	31	0.1	Strong binder	0.853
5	11	QVDRMRIRTWK	HLA-A*03:01	0.367	943	2.0	Weak binder	0.853
4	11	WQVDRMRIRTW	HLA-B*44:02	0.267	2779	3.0	Non-binder	0.853

Volunteer ID: 416

HLA-A*02:01, HLA-B*08:01, HLA-B*44:02, HLA-C*05:01, HLA-C*07:01

Pos	Length (aa)	Peptide	HLA	1-$\log_{50}$k (aff)	Affinity (nM)	% Rank	Label	Estimated Accuracy
**Peptide Sequence: KNPEIVIYQYMDDLYV HC093**								
8	9	YQYMDDLYV	HLA-A*02:01	0.788	10	0.4	Strong binder	0.853
6	11	VIYQYMDDLYV	HLA-A*02:01	0.682	31	1.0	Weak binder	0.853
3	8	PEIVIYQY	HLA-B*44:02	0.412	582	0.8	Weak binder	0.853
3	9	PEIVIYQYM	HLA-B*44:02	0.297	2004	2.0	Weak binder	0.853
8	8	YQYMDDLY	HLA-B*44:02	0.239	3759	3.0	Non-binder	0.853
8	9	YQYMDDLYV	HLA-C*07:01	0.135	NA	3.0	Non-binder	0.556
**Peptide Sequence: EQVDKLVSQGIRKVL HC134**								
5	10	KLVSQGIRKV	HLA-A*02:01	0.506	210	3.0	Combined binder	0.853
8	8	SQGIRKVL	HLA-B*08:01	0.343	1229	3.0	Non-binder	0.853
2	10	QVDKLVSQGI	HLA-C*05:01	0.452	378	2.0	Weak binder	0.853
**Peptide Sequence: KLVSQGIRKVLFLDG HC135**								
1	10	KLVSQGIRKV	HLA-A*02:01	0.506	210	3.0	Combined binder	0.853
6	8	GIRKVLFL	HLA-B*08:01	0.362	991	3.0	Non-binder	0.853

Pos	Length (aa)	Peptide	HLA	1-log$_{50}$k (aff)	Affinity (nM)	% Rank	Label	Estimated Accuracy
4	10	SQGIRKVLFL	HLA-B*08:01	0.356	1062	3.0	Non-binder	0.853
5	9	QGIRKVLFL	HLA-B*08:01	0.349	1147	3.0	Non-binder	0.853
4	8	SQGIRKVL	HLA-B*08:01	0.343	1229	3.0	Non-binder	0.853
**Peptide Sequence: VHVASGYIEAEVIPA HC151**								
7	9	YIEAEVIPA	HLA-A*02:01	0.465	328	4.0	Combined binder	0.853
2	11	HVASGYIEAEV	HLA-A*02:01	0.426	498	4.0	Combined binder	0.853
3	10	VASGYIEAEV	HLA-A*02:01	0.376	853	5.0	Non-binder	0.853
8	8	IEAEVIPA	HLA-B*44:02	0.324	1497	2.0	Weak binder	0.853
4	9	ASGYIEAEV	HLA-C*05:01	0.347	1169	4.0	Non-binder	0.853

Volunteer ID: 417

HLA-A*03:01, HLA-A*30:04, HLA-B*35:01, HLA-B*50:01, HLA-C*04:01, HLA-C*06:02

Pos	Length (aa)	Peptide	HLA	1-log$_{50}$k (aff)	Affinity (nM)	% Rank	Label	Estimated Accuracy
**Peptide Sequence: YFSVPLDEGFRKYTA: HC078**								
3	11	SVPLDEGFRKY	HLA-A*30:04	0.354	1085	3.0	Non-binder	0.753
4	10	VPLDEGFRKY	HLA-B*35:01	0.644	47	0.5	Strong binder	0.853
2	9	FSVPLDEGF	HLA-B*35:01	0.406	617	3.0	Non-binder	0.853
1	10	YFSVPLDEGF	HLA-C*04:01	0.12	NA	2.0	Weak binder	0.762
**Peptide Sequence: PLDEGFRKYTAFTIP: HC079**								
5	8	GFRKYTAF	HLA-A*30:04	0.338	1291	3.0	Non-binder	0.753
5	10	GFRKYTAFTI	HLA-A*30:04	0.315	1652	4.0	Non-binder	0.753
7	8	RKYTAFTI	HLA-B*50:01	0.393	714	2.0	Weak binder	0.701
5	8	GFRKYTAF	HLA-C*06:02	0.407	611	1.0	Weak binder	0.853
7	8	RKYTAFTI	HLA-C*06:02	0.357	1046	2.0	Weak binder	0.853
6	9	FRKYTAFTI	HLA-C*06:02	0.346	1187	2.0	Weak binder	0.853
5	10	GFRKYTAFTI	HLA-C*06:02	0.299	1977	4.0	Non-binder	0.853
**Peptide Sequence: KNPEIVIYQYMDDLYV HC093**								
6	10	VIYQYMDDLY	HLA-A*03:01	0.36	1021	3.0	Non-binder	0.853
5	11	IVIYQYMDDLY	HLA-A*03:01	0.264	2887	4.0	Non-binder	0.853
5	11	IVIYQYMDDLY	HLA-A*30:04	0.49	250	0.4	Strong binder	0.753
8	8	YQYMDDLY	HLA-A*30:04	0.473	300	0.5	Strong binder	0.753
7	9	IYQYMDDLY	HLA-A*30:04	0.455	365	0.8	Weak binder	0.753
6	10	VIYQYMDDLY	HLA-A*30:04	0.407	609	1.5	Weak binder	0.753
1	10	KNPEIVIYQY	HLA-A*30:04	0.373	885	2.0	Weak binder	0.753
2	9	NPEIVIYQY	HLA-B*35:01	0.772	12	0.1	Strong binder	0.853
2	10	NPEIVIYQYM	HLA-B*35:01	0.51	200	1.5	Weak binder	0.853
8	8	YQYMDDLY	HLA-B*35:01	0.494	238	1.5	Weak binder	0.853
5	11	IVIYQYMDDLY	HLA-B*35:01	0.405	627	3.0	Non-binder	0.853
8	8	YQYMDDLY	HLA-B*50:01	0.447	395	1.5	Weak binder	0.701
8	9	YQYMDDLYV	HLA-B*50:01	0.36	1023	2.0	Weak binder	0.701
9	8	QYMDDLYV	HLA-C*04:01	0.193	NA	0.5	Strong binder	0.762
7	10	IYQYMDDLYV	HLA-C*04:01	0.189	NA	0.5	Strong binder	0.762
7	9	IYQYMDDLY	HLA-C*04:01	0.115	NA	2.0	Weak binder	0.762

Pos	Length (aa)	Peptide	HLA	1-log_{50}k (aff)	Affinity (nM)	% Rank	Label	Estimated Accuracy
1	11	KNPEIVIYQYM	HLA-C*04:01	0.112	NA	2.0	Weak binder	0.762
7	8	IYQYMDDL	HLA-C*04:01	0.109	NA	3.0	Non-binder	0.762
8	9	YQYMDDLYV	HLA-C*04:01	0.083	NA	4.0	Non-binder	0.762
8	9	YQYMDDLYV	HLA-C*06:02	0.374	874	1.5	Weak binder	0.853
9	8	QYMDDLYV	HLA-C*06:02	0.325	1483	3.0	Non-binder	0.853
8	8	YQYMDDLY	HLA-C*06:02	0.309	1757	3.0	Non-binder	0.853
1	11	KNPEIVIYQYM	HLA-C*06:02	0.287	2239	4.0	Non-binder	0.853

Volunteer ID: 418

HLA-A*02:01, HLA-A*24:02, HLA-B*07:02, HLA-B*27:05, HLA-C*01:02, HLA-C*07:02

Pos	Length (aa)	Peptide	HLA	1-log50k (aff)	Affinity (nM)	% Rank	Label	Estimated Accuracy
**Peptide Sequence: EVIPMFTALSEGATP HC003**								
3	11	IPMFTALSEGA	HLA-B*07:02	0.44	430	1.5	Weak binder	0.853
3	10	IPMFTALSEG	HLA-B*07:02	0.343	1229	3.0	Non-binder	0.853
1	9	EVIPMFTAL	HLA-B*07:02	0.33	1402	3.0	Non-binder	0.853
3	9	IPMFTALSE	HLA-B*07:02	0.327	1446	3.0	Non-binder	0.853
3	8	IPMFTALS	HLA-B*07:02	0.264	2888	4.0	Non-binder	0.853
2	8	VIPMFTAL	HLA-C*01:02	0.491	247	0.12	Strong binder	0.698
2	8	VIPMFTAL	HLA-C*07:02	0.17	NA	3.0	Non-binder	0.541
1	9	EVIPMFTAL	HLA-C*07:02	0.158	NA	3.0	Non-binder	0.541
**Peptide Sequence: GLNKIVRMYSPVSIL HC017**								
7	8	RMYSPVSI	HLA-A*02:01	0.622	60	1.5	Weak binder	0.853
7	9	RMYSPVSIL	HLA-A*02:01	0.555	124	3.0	Combined binder	0.853
4	9	KIVRMYSPV	HLA-A*02:01	0.546	136	3.0	Combined binder	0.853
4	11	KIVRMYSPVSI	HLA-A*02:01	0.378	838	5.0	Non-binder	0.853
8	8	MYSPVSIL	HLA-A*24:02	0.694	27	0.25	Strong binder	0.853
7	8	RMYSPVSI	HLA-A*24:02	0.393	708	3.0	Non-binder	0.853
7	9	RMYSPVSIL	HLA-A*24:02	0.292	2124	4.0	Non-binder	0.853
5	11	IVRMYSPVSIL	HLA-B*07:02	0.539	147	0.8	Weak binder	0.853
5	10	IVRMYSPVSI	HLA-B*07:02	0.475	294	1.5	Weak binder	0.853
5	8	IVRMYSPV	HLA-B*07:02	0.423	512	1.5	Weak binder	0.853
7	9	RMYSPVSIL	HLA-B*07:02	0.4	661	2.0	Weak binder	0.853
7	8	RMYSPVSI	HLA-B*07:02	0.327	1452	3.0	Non-binder	0.853
6	10	VRMYSPVSIL	HLA-B*27:05	0.571	103	0.8	Weak binder	0.853
6	9	VRMYSPVSI	HLA-B*27:05	0.558	119	0.8	Weak binder	0.853
7	8	RMYSPVSI	HLA-B*27:05	0.386	771	3.0	Non-binder	0.853
7	9	RMYSPVSIL	HLA-B*27:05	0.377	844	3.0	Non-binder	0.853
6	8	VRMYSPVS	HLA-B*27:05	0.368	932	3.0	Non-binder	0.853
7	9	RMYSPVSIL	HLA-C*01:02	0.529	163	0.08	Strong binder	0.698
7	8	RMYSPVSI	HLA-C*01:02	0.463	335	0.25	Strong binder	0.698
8	8	MYSPVSIL	HLA-C*01:02	0.333	1369	2.0	Weak binder	0.698
5	11	IVRMYSPVSIL	HLA-C*01:02	0.316	1633	2.0	Weak binder	0.698
5	10	IVRMYSPVSI	HLA-C*01:02	0.277	2493	4.0	Non-binder	0.698
5	8	IVRMYSPV	HLA-C*01:02	0.254	3190	4.0	Non-binder	0.698
7	9	RMYSPVSIL	HLA-C*07:02	0.322	NA	0.17	Strong binder	0.541

Pos	Length (aa)	Peptide	HLA	1-log50k (aff)	Affinity (nM)	% Rank	Label	Estimated Accuracy
7	8	RMYSPVSI	HLA-C*07:02	0.276	NA	0.4	Strong binder	0.541
8	8	MYSPVSIL	HLA-C*07:02	0.255	NA	0.8	Weak binder	0.541
6	10	VRMYSPVSIL	HLA-C*07:02	0.151	NA	4.0	Non-binder	0.541
**Peptide Sequence: KNFPISPIETVPVKLK HC049**								
5	11	ISPIETVPVKL	HLA-A*24:02	0.311	1723	4.0	Non-binder	0.853
2	10	NFPISPIETV	HLA-A*24:02	0.307	1812	4.0	Non-binder	0.853
6	8	SPIETVPV	HLA-B*07:02	0.583	91	0.8	Weak binder	0.853
3	11	FPISPIETVPV	HLA-B*07:02	0.555	124	0.8	Weak binder	0.853
6	10	SPIETVPVKL	HLA-B*07:02	0.542	142	0.8	Weak binder	0.853
3	9	FPISPIETV	HLA-B*07:02	0.414	565	1.5	Weak binder	0.853
3	8	FPISPIET	HLA-B*07:02	0.311	1736	3.0	Non-binder	0.853
5	9	ISPIETVPV	HLA-C*01:02	0.403	638	0.8	Weak binder	0.698
5	11	ISPIETVPVKL	HLA-C*01:02	0.349	1143	1.5	Weak binder	0.698
3	11	FPISPIETVPV	HLA-C*07:02	0.225	NA	1.0	Weak binder	0.541
5	9	ISPIETVPV	HLA-C*07:02	0.164	NA	3.0	Non-binder	0.541
6	8	SPIETVPV	HLA-C*07:02	0.147	NA	4.0	Non-binder	0.541
**Peptide Sequence: NPEIVIYQYMDDLYV HC093**								
7	9	YQYMDDLYV	HLA-A*02:01	0.788	10	0.4	Strong binder	0.853
5	11	VIYQYMDDLYV	HLA-A*02:01	0.682	31	1.0	Weak binder	0.853
6	10	IYQYMDDLYV	HLA-A*24:02	0.565	110	0.8	Weak binder	0.853
6	8	IYQYMDDL	HLA-A*24:02	0.537	150	1.0	Weak binder	0.853
6	9	IYQYMDDLY	HLA-A*24:02	0.51	201	1.0	Weak binder	0.853
8	8	QYMDDLYV	HLA-A*24:02	0.507	207	1.5	Weak binder	0.853
1	10	NPEIVIYQYM	HLA-B*07:02	0.27	2690	4.0	Non-binder	0.853
7	8	YQYMDDLY	HLA-B*27:05	0.295	2062	4.0	Non-binder	0.853
7	9	YQYMDDLYV	HLA-C*07:02	0.197	NA	1.5	Weak binder	0.541
8	8	QYMDDLYV	HLA-C*07:02	0.169	NA	3.0	Non-binder	0.541
**Peptide Sequence: VIYQYMDDLYVGSDL HC094**								
3	9	YQYMDDLYV	HLA-A*02:01	0.788	10	0.4	Strong binder	0.853
5	11	YMDDLYVGSDL	HLA-A*02:01	0.766	13	0.5	Strong binder	0.853
1	11	VIYQYMDDLYV	HLA-A*02:01	0.682	31	1.0	Weak binder	0.853
5	9	YMDDLYVGS	HLA-A*02:01	0.653	43	1.5	Weak binder	0.853
5	8	YMDDLYVG	HLA-A*02:01	0.531	159	3.0	Combined binder	0.853
2	10	IYQYMDDLYV	HLA-A*24:02	0.565	110	0.8	Weak binder	0.853
2	8	IYQYMDDL	HLA-A*24:02	0.537	150	1.0	Weak binder	0.853
2	9	IYQYMDDLY	HLA-A*24:02	0.51	201	1.0	Weak binder	0.853
4	8	QYMDDLYV	HLA-A*24:02	0.507	207	1.5	Weak binder	0.853
2	11	IYQYMDDLYVG	HLA-A*24:02	0.307	1798	4.0	Non-binder	0.853
4	9	QYMDDLYVG	HLA-A*24:02	0.275	2545	4.0	Non-binder	0.853
3	8	YQYMDDLY	HLA-B*27:05	0.295	2062	4.0	Non-binder	0.853
5	11	YMDDLYVGSDL	HLA-C*01:02	0.422	520	0.5	Strong binder	0.698
5	11	YMDDLYVGSDL	HLA-C*07:02	0.206	NA	1.5	Weak binder	0.541
3	9	YQYMDDLYV	HLA-C*07:02	0.197	NA	1.5	Weak binder	0.541
4	8	QYMDDLYV	HLA-C*07:02	0.169	NA	3.0	Non-binder	0.541

Volunteer ID: 420

HLA-A*02:01, HLA-A*29:02, HLA-B*44:03, HLA-B*55:01, HLA-C*03:03, HLA-C*16:01

Pos	Length (aa)	Peptide	HLA	1-log50k (aff)	Affinity (nM)	% Rank	Label	Estimated Accuracy
**Peptide Sequence: KNPEIVIYQYMDDLYV HC093**								
8	9	YQYMDDLYV	HLA-A*02:01	0.788	10	0.4	Strong binder	0.853
6	11	VIYQYMDDLYV	HLA-A*02:01	0.682	31	1.0	Weak binder	0.853
8	8	YQYMDDLY	HLA-A*29:02	0.748	15	0.2	Strong binder	0.853
6	10	VIYQYMDDLY	HLA-A*29:02	0.739	17	0.2	Strong binder	0.853
5	11	IVIYQYMDDLY	HLA-A*29:02	0.728	19	0.25	Strong binder	0.853
7	9	IYQYMDDLY	HLA-A*29:02	0.6	76	0.8	Weak binder	0.853
1	10	KNPEIVIYQY	HLA-A*29:02	0.357	1048	3.0	Non-binder	0.853
3	8	PEIVIYQY	HLA-B*44:03	0.492	244	0.8	Weak binder	0.853
3	9	PEIVIYQYM	HLA-B*44:03	0.351	1119	2.0	Weak binder	0.853
8	8	YQYMDDLY	HLA-B*44:03	0.316	1637	2.0	Weak binder	0.853
8	9	YQYMDDLYV	HLA-C*03:03	0.484	266	4.0	Combined binder	0.694
6	11	VIYQYMDDLYV	HLA-C*03:03	0.395	697	7.0	Non-binder	0.694
8	9	YQYMDDLYV	HLA-C*16:01	0.531	160	4.0	Combined binder	0.734
8	8	YQYMDDLY	HLA-C*16:01	0.482	271	6.0	Combined binder	0.734
6	11	VIYQYMDDLYV	HLA-C*16:01	0.48	278	6.0	Combined binder	0.734
9	8	QYMDDLYV	HLA-C*16:01	0.452	378	7.0	Combined binder	0.734
6	9	VIYQYMDDL	HLA-C*16:01	0.448	391	7.0	Combined binder	0.734
6	10	VIYQYMDDLY	HLA-C*16:01	0.412	580	9.0	Non-binder	0.734
1	11	KNPEIVIYQYM	HLA-C*16:01	0.402	645	9.0	Non-binder	0.734
7	10	IYQYMDDLYV	HLA-C*16:01	0.386	772	10.0	Non-binder	0.734
**Peptide Sequence: VIYQYMDDLYVGSDL HC094**								
3	9	YQYMDDLYV	HLA-A*02:01	0.788	10	0.4	Strong binder	0.853
5	11	YMDDLYVGSDL	HLA-A*02:01	0.766	13	0.5	Strong binder	0.853
1	11	VIYQYMDDLYV	HLA-A*02:01	0.682	31	1.0	Weak binder	0.853
5	9	YMDDLYVGS	HLA-A*02:01	0.653	43	1.5	Weak binder	0.853
5	8	YMDDLYVG	HLA-A*02:01	0.531	159	3.0	Combined binder	0.853
3	8	YQYMDDLY	HLA-A*29:02	0.748	15	0.2	Strong binder	0.853
1	10	VIYQYMDDLY	HLA-A*29:02	0.739	17	0.2	Strong binder	0.853
2	9	IYQYMDDLY	HLA-A*29:02	0.6	76	0.8	Weak binder	0.853
3	8	YQYMDDLY	HLA-B*44:03	0.316	1637	2.0	Weak binder	0.853
5	11	YMDDLYVGSDL	HLA-C*03:03	0.695	27	0.1	Strong binder	0.694
3	9	YQYMDDLYV	HLA-C*03:03	0.484	266	4.0	Combined binder	0.694
1	11	VIYQYMDDLYV	HLA-C*03:03	0.395	697	7.0	Non-binder	0.694
5	11	YMDDLYVGSDL	HLA-C*16:01	0.691	28	0.5	Strong binder	0.734
3	9	YQYMDDLYV	HLA-C*16:01	0.531	160	4.0	Combined binder	0.734
3	8	YQYMDDLY	HLA-C*16:01	0.482	271	6.0	Combined binder	0.734
1	11	VIYQYMDDLYV	HLA-C*16:01	0.48	278	6.0	Combined binder	0.734
4	8	QYMDDLYV	HLA-C*16:01	0.452	378	7.0	Combined binder	0.734
1	9	VIYQYMDDL	HLA-C*16:01	0.448	391	7.0	Combined binder	0.734
5	8	YMDDLYVG	HLA-C*16:01	0.421	525	8.0	Non-binder	0.734
1	10	VIYQYMDDLY	HLA-C*16:01	0.412	580	9.0	Non-binder	0.734
2	10	IYQYMDDLYV	HLA-C*16:01	0.386	772	10.0	Non-binder	0.734

Pos	Length (aa)	Peptide	HLA	1-log50k (aff)	Affinity (nM)	% Rank	Label	Estimated Accuracy
**Peptide Sequence: VQMAVFIHNFKRKGGI HC164**								
2	9	QMAVFIHNF	HLA-A*29:02	0.479	280	1.5	Weak binder	0.853
1	10	VQMAVFIHNF	HLA-A*29:02	0.396	685	3.0	Non-binder	0.853
3	9	MAVFIHNFK	HLA-A*29:02	0.378	836	3.0	Non-binder	0.853
3	10	MAVFIHNFKR	HLA-A*29:02	0.375	862	3.0	Non-binder	0.853
3	8	MAVFIHNF	HLA-A*29:02	0.333	1368	4.0	Non-binder	0.853
2	10	QMAVFIHNFK	HLA-A*29:02	0.321	1559	4.0	Non-binder	0.853
2	11	QMAVFIHNFKR	HLA-A*29:02	0.313	1693	4.0	Non-binder	0.853
1	11	VQMAVFIHNFK	HLA-A*29:02	0.312	1715	4.0	Non-binder	0.853
5	8	VFIHNFKR	HLA-A*29:02	0.291	2139	4.0	Non-binder	0.853
1	10	VQMAVFIHNF	HLA-B*44:03	0.38	820	1.5	Weak binder	0.853
3	8	MAVFIHNF	HLA-B*51:01	0.24	NA	2.0	Weak binder	0.853
3	8	MAVFIHNF	HLA-C*03:03	0.412	580	6.0	Non-binder	0.694
1	10	VQMAVFIHNF	HLA-C*03:03	0.373	880	8.0	Non-binder	0.694
1	10	VQMAVFIHNF	HLA-C*16:01	0.598	77	2.0	Weak binder	0.734
3	8	MAVFIHNF	HLA-C*16:01	0.572	103	3.0	Combined binder	0.734
2	9	QMAVFIHNF	HLA-C*16:01	0.544	139	3.0	Combined binder	0.734

Volunteer ID: 421

HLA-A*02:01, HLA-A*11:01, HLA-B*35:03, HLA-B*40:02, HLA-C*02:02, HLA-C*12:03

Pos	Length (aa)	Peptide	HLA	1-log50k (aff)	Affinity (nM)	% Rank	Label	Estimated Accuracy
**Peptide Sequence: CTERQANFLGKIWPS HC031**								
8	8	FLGKIWPS	HLA-A*02:01	0.558	120	3.0	Combined binder	0.853
4	8	RQANFLGK	HLA-A*11:01	0.656	41	0.8	Weak binder	0.853
1	11	CTERQANFLGK	HLA-A*11:01	0.629	55	0.8	Weak binder	0.853
2	8	TERQANFL	HLA-B*40:02	0.551	129	1.5	Weak binder	0.853
2	11	TERQANFLGKI	HLA-B*40:02	0.499	226	2.0	Weak binder	0.853
4	9	RQANFLGKI	HLA-B*40:02	0.499	227	2.0	Weak binder	0.853
4	10	RQANFLGKIW	HLA-B*40:02	0.41	590	4.0	Non-binder	0.853
4	9	RQANFLGKI	HLA-C*02:02	0.379	826	7.0	Non-binder	0.706
1	8	CTERQANF	HLA-C*02:02	0.366	951	7.0	Non-binder	0.706
1	9	CTERQANFL	HLA-C*02:02	0.363	983	8.0	Non-binder	0.706
4	9	RQANFLGKI	HLA-C*12:03	0.411	585	9.0	Non-binder	0.730
**Peptide Sequence: YFSVPLDEGFRKYTA HC078**								
2	11	FSVPLDEGFRK	HLA-A*11:01	0.512	196	2.0	Weak binder	0.853
3	10	SVPLDEGFRK	HLA-A*11:01	0.493	242	3.0	Combined binder	0.853
4	10	VPLDEGFRKY	HLA-B*35:03	0.173	NA	2.0	Weak binder	0.811
2	9	FSVPLDEGF	HLA-C*02:02	0.569	106	0.8	Weak binder	0.706
2	9	FSVPLDEGF	HLA-C*12:03	0.481	274	6.0	Combined binder	0.730
1	10	YFSVPLDEGF	HLA-C*12:03	0.366	953	15.0	Non-binder	0.730
**Peptide Sequence: GSPAIFQSSMTKILE HC088**								
6	8	FQSSMTKI	HLA-A*02:01	0.443	412	4.0	Combined binder	0.853

Pos	Length (aa)	Peptide	HLA	1-log50k (aff)	Affinity (nM)	% Rank	Label	Estimated Accuracy
4	10	AIFQSSMTKI	HLA-A*02:01	0.398	671	5.0	Non-binder	0.853
4	9	AIFQSSMTK	HLA-A*11:01	0.813	8	0.05	Strong binder	0.853
5	8	IFQSSMTK	HLA-A*11:01	0.47	309	3.0	Combined binder	0.853
3	10	PAIFQSSMTK	HLA-A*11:01	0.395	698	4.0	Non-binder	0.853
2	9	SPAIFQSSM	HLA-B*35:03	0.378	NA	0.2	Strong binder	0.811
2	8	SPAIFQSS	HLA-B*35:03	0.156	NA	3.0	Non-binder	0.811
2	10	SPAIFQSSMT	HLA-B*35:03	0.119	NA	4.0	Non-binder	0.811
6	9	FQSSMTKIL	HLA-B*40:02	0.371	899	4.0	Non-binder	0.853
1	10	GSPAIFQSSM	HLA-C*02:02	0.483	270	3.0	Combined binder	0.706
4	10	AIFQSSMTKI	HLA-C*02:02	0.426	500	4.0	Non-binder	0.706
6	8	FQSSMTKI	HLA-C*02:02	0.384	781	6.0	Non-binder	0.706
4	10	AIFQSSMTKI	HLA-C*12:03	0.565	110	3.0	Combined binder	0.730
4	11	AIFQSSMTKIL	HLA-C*12:03	0.537	150	4.0	Combined binder	0.730
1	10	GSPAIFQSSM	HLA-C*12:03	0.523	175	4.0	Combined binder	0.730
6	9	FQSSMTKIL	HLA-C*12:03	0.384	785	15.0	Non-binder	0.730
6	8	FQSSMTKI	HLA-C*12:03	0.38	817	15.0	Non-binder	0.730
**Peptide Sequence: KNPEIVIYQYMDDLYV HC093**								
8	9	YQYMDDLYV	HLA-A*02:01	0.788	10	0.4	Strong binder	0.853
6	11	VIYQYMDDLYV	HLA-A*02:01	0.682	31	1.0	Strong binder	0.853
10	11	YMDDLYVSDIK	HLA-A*11:01	0.453	371	3.0	Weak binder	0.853
15	10	YVSDIKVVPR	HLA-A*11:01	0.44	429	3.0	Weak binder	0.853
6	10	VIYQYMDDLY	HLA-A*11:01	0.429	482	3.0	Weak binder	0.853
5	11	IVIYQYMDDLY	HLA-A*11:01	0.419	539	3.0	Non-binder	0.853
8	9	YQYMDDLYV	HLA-B*40:02	0.438	436	3.0	Weak binder	0.853
3	9	PEIVIYQYM	HLA-B*40:02	0.369	926	5.0	Non-binder	0.853
8	8	YQYMDDLY	HLA-C*02:02	0.57	105	0.8	Weak binder	0.706
8	9	YQYMDDLYV	HLA-C*02:02	0.467	321	3.0	Weak binder	0.706
6	10	VIYQYMDDLY	HLA-C*02:02	0.425	502	4.0	Non-binder	0.706
6	11	VIYQYMDDLYV	HLA-C*02:02	0.415	559	5.0	Non-binder	0.706
8	9	YQYMDDLYV	HLA-C*12:03	0.568	108	3.0	Weak binder	0.730
6	11	VIYQYMDDLYV	HLA-C*12:03	0.511	199	5.0	Weak binder	0.730
6	9	VIYQYMDDL	HLA-C*12:03	0.48	279	6.0	Weak binder	0.730
8	8	YQYMDDLY	HLA-C*12:03	0.473	298	6.0	Weak binder	0.730
9	8	QYMDDLYV	HLA-C*12:03	0.424	509	9.0	Non-binder	0.730
6	10	VIYQYMDDLY	HLA-C*12:03	0.411	585	9.0	Non-binder	0.730
**Peptide Sequence: SDIKVVPRRKAKIIR HC175**								
4	9	KVVPRRKAK	HLA-A*11:01	0.462	338	3.0	Weak binder	0.853

a – Prediction were performed using HLArestrictor with NetMHCpan version 2.4

b - Peptide lengths: **8, 9, 10, 11**

c - Sort-method: OR. Sort-mode: HLA-oriented

d - %rank threshold for strong binding peptides: 0.5% rank and weak binding peptides: 2.0% rank

e - Affinity threshold for strong binding peptides: 50 nM and weak binding peptides: 500 nM

f - Number of predictions per peptide: Not specified

g - Non-binders shown up to a prediction score of 2.0 (weak binding threshold)
